# Identification of protein targets for dyslipidaemia and cardiovascular diseases among people with South Asian ancestry: a mendelian randomisation study

**DOI:** 10.1016/j.lansea.2025.100621

**Published:** 2025-07-04

**Authors:** Siwei Wu, Devendra Meena, Alexander Smith, Jingxian Huang, Georg W. Otto, Yi-Hsuan Ko, James Yarmolinsky, Dipender Gill, Anand Rohatgi, Abbas Dehghan, Ioanna Tzoulaki

**Affiliations:** a Department of Clinical Nutrition, Shanghai Children’s Medical Center, Shanghai Jiao Tong University School of Medicine, China; b Department of Epidemiology and Biostatistics, School of Public Health, Imperial College London, London, UK; c Department of Medicine, Division of Cardiology University of Texas Southwestern Medical Center Dallas TX, USA; d UK Dementia Research Institute at Imperial College London, London, UK; e British Heart Foundation Centre of Research Excellence, Imperial College London, London, UK; f Centre for Systems Biology, Biomedical Research Foundation, Academy of Athens, Athens, Greece

**Keywords:** Dyslipidemia, South Asians, Mendelian randomization, Proteomics, South Asian ancestry, Asian ancestry, Cardiovascular disease risk

## Abstract

**Background:**

South Asians are considered to be at higher risk of dyslipidaemia, a modifiable risk factor for cardiovascular diseases (CVDs). We aimed to identify protein targets for dyslipidaemia and CVDs among people with South Asian ancestry.

**Methods:**

We used a two-sample mendelian randomisation (MR) approach, supplemented with MR-Egger, weighted median, colocalisation, and generalised MR (GMR), to evaluate the effect of 2800 plasma proteins on high/low/non-high-density lipoprotein cholesterol (HDL-C/LDL-C/non-HDL-C), total cholesterol, and triglycerides. Observational analyses were conducted on MR findings with strong colocalisation (posterior probability ≥ 80%) and GMR evidence. Univariate MR assessed lipid-associated proteins' effect on CVDs. Finally, we compared the effects of plasma proteins on lipids between South Asian and European populations.

**Findings:**

We identified 29 genetically proxied proteins potentially causal to at least one lipid measure, 12 of which showed strong colocalisation and GMR evidence, including angiopoietin-related protein 3 (ANGPTL3), proprotein convertase subtilisin/kexin type 9 (PCSK9), and cadherin EGF LAG seven-pass G-type receptor 2 (CELSR2). Notably, PCSK9 demonstrated a stronger association with LDL-C in Europeans compared to South Asians (βEuropean = 0.37; 95% CI 0.36, 0.38, βSouth Asian = 0.16; 95% CI 0.11, 0.21). Observational analysis suggested statistically significant interaction between PCSK9 levels with LDL-C levels in South Asians with South Asians having a significantly lower effect compared to other ethnicities (PCSK9∗South Asian; β = −0.14; 95% CI -0.174, −0.107). Additionally, we showed that CELSR2 is also linked with coronary artery disease in South Asians.

**Interpretation:**

Our study highlighted potential causal links between plasma proteins, dyslipidaemia, and CVDs in South Asians and highlighted protein targets, including CELSR2, PCSK9, ANGPTL3, and Apolipoprotein(a) (LPA). Notably, our study indicated that PCSK9 has a significantly weaker effect on LDL-C in South Asians than Europeans.

**Funding:**

This work is supported by the 10.13039/501100000274British Heart Foundation Research Excellence Award (4) (RE/24/130023). IT and AR are supported by 10.13039/100000002NIHR01 HL162300-02.

**Research in context**

**Evidence before this study**

Previous studies using Mendelian randomisation (MR) and observational analyses have identified potential causal associations between circulating plasma proteins, lipid traits and cardiovascular diseases. However, most genetic studies (including MR analyses) have focused on populations of European ancestry, limiting genetic insight in diverse ancestries. Limited data exist on the causal relevance of these proteins in South Asians, who have a distinct genetic background and a high burden of dyslipidaemia and subsequent cardiovascular diseases. To the best of our knowledge, no large-scale MR study has systematically evaluated the causal effects of thousands of circulating plasma proteins on the five major lipid fractions and cardiovascular outcomes in South Asian populations.

**Added value of this study**

This is the first large-scale MR study to systematically evaluate the potential causal effects of over 2800 circulating plasma proteins on five major lipid fractions and cardiovascular outcomes in South Asian populations. By focusing on a population with a high burden of dyslipidaemia and cardiovascular disease and using robust genetic variants (protein quantitative trait loci) in MR, our study provides ancestry-specific insights into the proteomic architecture of lipid traits and cardiovascular diseases. Notably, our findings highlight proprotein convertase subtilisin/kexin type 9 (PCSK9), which showed a stronger association with low-density lipoprotein cholesterol (LDL-C) in Europeans than in South Asians (β_European = 0.37; 95% CI: 0.36–0.38 vs. β_South Asian = 0.16; 95% CI: 0.11–0.21), with observational analyses also indicating a significant ethnicity-specific interaction. These findings offer a valuable resource for prioritizing protein targets underlying lipid metabolism and cardiovascular risk in South Asians.

**Implications of all the available evidence**

Our findings highlight the importance of ancestry-specific genetic studies in identifying potential causal proteins linked to dyslipidaemia and cardiovascular diseases. This work prioritises protein targets in this high-risk population and supports the need for tailored therapeutic strategies for South Asians.

## Introduction

Routinely measured lipid parameters including high/low/non-high-density lipoprotein cholesterol (HDL-C/LDL-C/non-HDL-C), total cholesterol (TC), and triglycerides (TG), are well-established risk factors for cardiovascular diseases (CVDs), including coronary artery disease (CAD), stroke, and heart failure,^1^, ^2^, ^3^, ^4^ all of which are leading causes of mortality and morbidity worldwide.^5^ HDL-C and TC have been included in several risk equations like SCORE and PCE as important predictors for CVDs.^6^^,^^7^ The role of HDL-C and TG as the target for CVD intervention is inconclusive but under investigation.^8^^,^^9^ LDL-C and non-HDL-C are not only prognostic risk markers but also established therapeutic targets for CVDs.^10^^,^^11^ Building on this, evidence suggests that individuals of South Asian ancestry, compared with non-Hispanic white population, have a higher prevalence of dyslipidaemia^12^ and are more susceptible to cardiometabolic diseases closely related to dyslipidaemia including CAD,^13^^,^^14^ stroke,^15^ and type 2 diabetes.^16^ Therefore, understanding the genomic and proteomic makeup of plasma lipids and identifying causal factors in South Asian ancestral population is crucial for intervening in dyslipidaemia and preventing lipid-related cardiovascular conditions although the residual risk of lipid modifying medications remains.

Circulating plasma proteins are key to disease mechanisms and are promising drug targets. Previous studies have identified proteins associated with lipids, some of which are targeted for dyslipidaemia treatment.^17^ The most prominent example is Apolipoprotein B (APOB), which is one of the most prognostic and best therapeutic targets for CVDs.^18^ Despite recent advances, most plasma proteins linked to lipids were discovered in European population due to the greater availability of proteomic and genomic data.^19^ Proteomic studies, reported in non-European individuals, are limited, especially in South Asians — a rapidly growing but highly underrepresented population in genomic studies, despite their heightened susceptibility to dyslipidaemia.^12^ However, investigating non-European populations holds the potential to identify novel therapeutical targets for disease outcomes. We recently reported that certain plasma proteins may exert ancestry-specific causal effects on certain CVDs.^20^ Therefore, understanding the proteomic features of dyslipidaemia in South Asians is essential for developing more effective disease prevention strategies and drug discovery approaches for this high-risk population.

Recent genome wide association studies (GWAS) involving South Asian populations have made resources on circulating plasma proteins and lipid traits available.^21^^,^^22^ Furthermore, advances in methodologies for causal inference in epidemiology, including the mendelian randomisation (MR) framework, now allow us to investigate the potential causal relationship between an exposure (e.g., plasma proteins) and an outcome (e.g., lipid traits) for which GWAS summary statistics are available.^23^ MR utilises genetic variants as instrumental variables (IVs), and is less susceptible to confounders and reverse causation bias than other study designs, and can be applied for causal inference.^24^ MR is analogous to randomised controlled trials and can be used to assess the potential causal effect between exposures (e.g., protein abundance), and outcomes (e.g., lipid fractions).

In this study, we aimed to: (i) systematically evaluate the bi-directional causal effects of plasma proteins on five commonly measured lipid fractions (HDL-C, LDL-C, non-HDL-C, TC, and TG) in individuals of South Asian ancestry using a two-sample proteome-wide MR approach; (ii) investigate whether lipid-associated plasma proteins affect the risk of CAD and stroke in individuals of South Asian ancestry, and assess the causal effects of lipid fractions on CVDs; and (iii) compare the potential causal effects of plasma proteins on lipid fractions in South Asian and European ancestral populations in order to identify ancestry-specific markers.

## Methods

### Data source

#### Genetic associations for genetically predicted plasma proteins

In the UK Biobank Pharma Proteomics Project (UKBPPP), 2940 probes capturing 2922 unique proteins were made available.^22^ We defined each probe as a protein and extracted genetic associations of the 2940 plasma proteins in individuals of Central/South Asian ancestry (N = 920) and European ancestry (N = 34,557) from the UKBPPP.^22^ We included the 2800 plasma proteins as the primary exposures after excluding those with ambiguous cognate genes (N = 15) and those encoded by genes lying on the X chromosome (due to unavailability of X chromosome data for the outcomes; N = 88) or the major histocompatibility complex (MHC) region (due to complex linkage disequilibrium (LD) in this region; N = 37).

#### Genetic associations for genetically predicted lipid traits and CVDs

Five lipid traits including HDL-C, LDL-C, non-HDL-C, TC, and TG were included as the primary outcomes in MR analysis. Ancestry-specific (NSouth Asian = up to 40,963, NEuropean = 1,320,016) genetic associations of the 5 traits were publicly available from the Global Lipids Genetics Consortium (GLGC).^21^ The GWAS statistics for CAD were obtained from the East London Genes & Health (ELGH) study for South Asians.^25^ The GWAS summary data on stroke for South Asians were sourced from the GIGASTROKE consortium.^26^ A detailed description of the ancestral background of participants in the GWAS for proteomics, lipid fractions and CVDs can be found in the Supplementary Methods. However, all ancestry background in this study refers to genetically determined ancestry. Although adjusting for population structure is not feasible in a summary data–based two-sample MR study, appropriate measures were taken in the original GWAS to address ancestral diversity. For example, the plasma proteomics GWAS by Sun and colleagues adjusted for the first 20 genetic principal components to account for population structure.^22^ The studies used in our analysis were approved by their respective institutional review boards, and informed consent was provided by all participants.

### Proteome-wide MR and colocalisation analysis on lipid fractions in South Asians

#### Instrument selection and MR

We conducted a two-sample proteome-wide MR to evaluate the potential causal effect of circulating plasma proteins on the five lipid fractions mentioned above. Two-Sample MR is an approach that can be leveraged to assess the potential causal effect of an exposure on an outcome using genetic variants as IVs. The method can produce unbiased causal estimates, provided that the three core assumptions hold for the IVs: (i) the relevance assumption: the IV is robustly associated with the exposure, (ii) the independence assumption: the IV is independent of the confounders that confound the association between the exposure and outcome, (iii) the exclusion–restriction assumption: the IV affects the outcome only through the exposure (no horizontal pleiotropy).^24^

In this study, we used strong (*P* < 5 × 10^−8^), independent cis-protein quantitative trait locus (cis-pQTLs) as instruments for the proteome-wide MR. A detailed description of the instrument selection process can be found in Supplementary Methods. Briefly, pQTLs were selected at *P* < 5 × 10^−8^, clumped at r^2^ = 0.001 using ancestry-specific reference panels (1000 genomes phase 3; South Asian)^27^ and excluded if failed in Steiger filtering test.^28^ Following these criteria for IV selection, a total of 708 proteins had at least one genetic instrument and were carried forward for MR analysis (Supplementary Fig. S1). Given that we used strong cis-pQTLs as IVs, the first MR assumption (that the IVs are strongly associated with the exposure (plasma protein levels)) is satisfied. The use of cis-acting variants also reduces the likelihood of horizontal pleiotropy, addressing the third assumption. Additionally, the second assumption is reasonably met, as genetic variants are randomly allocated at conception, minimising confounding. The Wald ratio method was applied to proteins with single SNP as the instrument while the inverse-variance weighted (IVW) model was applied to proteins which were instrumented by two or more SNPs.^23^ To test for horizontal pleiotropy and to ensure robustness of the proteome-wide MR findings, we applied MR-Egger and weighted median to the statistically significant associations where at least three instruments were available.^29^^,^^30^ For multiple testing correction, we calculated both Benjamini-Hochberg false discovery rate (FDR) and Bonferroni corrected family wise error rate (FWER) for each lipid fraction.^31^ Given strong correlation between some plasma proteins, we defined significance as FDR < 0.05.

To avoid bias due to sample overlap between UKBPPP and GLGC, we also performed MR using GLGC data excluding UKBB participants. A correlation analysis was performed to compare the beta estimates derived from GLGC data with or without UKBB individuals.

#### Bayesian colocalisation

A Bayesian colocalisation analysis was conducted on all protein-lipid associations with FDR-corrected *P* < 0.05 to determine if they shared the same causal variant.^32^ Bayesian colocalisation is a statistical approach that assesses whether two or more traits (e.g., a plasma protein such as PCSK9 and a lipid fraction such as LDL-C) share a single or distinct causal variant at a given locus, using summary-level data from GWAS. It is routinely employed in the field of causal inference to validate MR findings and helps minimise horizontal pleiotropy caused by LD, where two traits are influenced by distinct, but correlated, genetic variants.^33^ In this study, colocalisation was performed on the same window as the previous proteome-wide MR (within ± 500 KB of the cognate gene), with rare variants (MAF < 0.05) residing in the window dropped. Default priors as described in the original paper were applied.^32^ A posterior probability of colocalisation (PPH4) ≥ 80% indicated strong colocalisation, while 60% ≤ PPH4 < 80% was considered suggestive evidence for colocalisation.

#### MR generalised to correlated instruments (GMR)

Due to stringent genetic instrument selection, most proteins had fewer pQTLs as IVs, which could bias the MR estimates due to unknown pleiotropy and make MR-Egger and weighted median unapplicable. Therefore, to improve robustness, we performed MR with more liberal instrument selection criteria. Specifically, we ran GMR on *cis*-pQTLs significant at *P* < 1 × 10^−4^ and clumped them to r^2^ = 0.4.^34^ For further sensitivity check, we ran separate GMR analysis under moderate instrument selection criterion and *cis*-pQTLs with *P* < 5 × 10^−6^ were included and clumped to r^2^ = 0.1.

To guard against weak instrument bias, we calculated the F-statistics for each genetic instrument and only included IVs if the F-statistic ≥ 10. To account for correlation between instruments, a generalised inverse variance method (gIVW, i.e., weighted generalised linear regression) was applied.^35^ To supplement the GMR findings, we also applied Generalised Summary-data-based MR (GSMR) tailored for correlated instruments.^36^ Where applicable, we also performed the MR-Egger generalised to correlated variants (gEgger)^37^ and weighted median.^30^

### Causal effects of lipid-associated proteins on CAD and stroke

A *cis*-MR analysis was conducted with lipid-associated plasma proteins (*P*-FDR < 0.05) as exposures and CAD and stroke (any stroke, any ischemic stroke, large artery stroke, cardioembolic stroke, and small vessel stroke) as outcomes. An FDR correction was applied separately for each outcome while colocalisation and supplemental MR methods (gIVW, gEgger, and weighted median) were performed to validate the genetically proxied associations surviving the 5% FDR correction.^30^, ^31^, ^32^^,^^35^^,^^37^ The instrument selection criteria and parameters for MR and colocalisation in this step were set as described above.

Additionally, univariate MR was performed to assess whether the lipid fractions show similar causal effects on CVDs in South Asians compared to Europeans. Genetic instruments for lipid fractions were extracted from the whole genome (autosomes only), and other MR criteria and methods were the same as those applied in *cis*-MR.^28^^,^^38^ Where applicable, MR-Egger and weighted median were applied as well.^29^^,^^30^

Furthermore, for plasma proteins associated with both lipids and CVDs, we evaluated with multi-trait colocalisation whether the three traits have a shared causal variant in the corresponding gene region.^39^ The multi-trait colocalisation analysis was applied on plasma protein, lipid fractions, and the CVD on the genomic region ± 500 KB extended from the cognate gene.^39^ Rare variants with MAF < 0.05 were dropped. Priors for multi-trait colocalisation were set to default values: the probability of any SNP within the colocalisation window being exclusively associated with one of the three traits was 1 × 10^−4^, with two traits was 1 × 10^−6^, and with all three traits was 1 × 10^−7^.^39^

### Reverse MR with lipid fractions as exposures and plasma proteins as outcomes in South Asians

Furthermore, to understand the potential causal effects of dyslipidaemia on plasma protein abundance, we conducted reverse MR using lipid fractions as exposures and plasma proteins as outcomes. Genetic instruments were selected from the 22 autosomes using the filtering criteria as previously described.

### Comparison with European population

For plasma proteins with potential effects on lipid fractions in South Asians (i.e., identified by proteome-wide MR), we estimated their effects in Europeans using a two-sample MR approach, applying the same genetic instrument criteria used in South Asians but using a European specific reference panel in 1000 Genomes Phase 3.^27^ We checked the consistency of the MR estimates and applied a correlation analysis on ancestry specific estimates that were consistent and statistically significant in both ancestral groups.

### Observational associations of protein levels with lipid fractions

For plasma proteins associated to LDL-C and HDL-C showing significant MR, strong colocalisation, and GMR evidence, we conducted observational analysis using linear regression for each protein and its associated lipid fraction. Each regression model was adjusted for age, sex, Townsend deprivation, BMI, glycated haemoglobin (HbA1c), cholesterol medication, smoking, systolic blood pressure, blood pressure medication, and ethnicity. The variables included as covariates in the models were based on already well-established associations of cardiovascular disease with patient demographics (age, sex, ethnicity, deprivation) and cardiovascular risk factors; BMI, high cholesterol (via medication), blood pressure (systolic and medication), smoking and diabetes (via HbA1c). To evaluate potential effect modification by ancestry, we included an interaction term between a binary variable for South Asian ancestry and protein level. All continuous variables and lipid fractions were standardised before modelling and *P* values were adjusted for multiple testing using false discovery rate (FDR) at 5%.

### Role of the funding source

The funder of the study had no role in the study design, data collection, data analysis, data interpretation, or writing of the report.

## Results

### Proteome-wide MR identified 29 plasma proteins associated with lipid fractions in South Asians

**Fig. 1:**
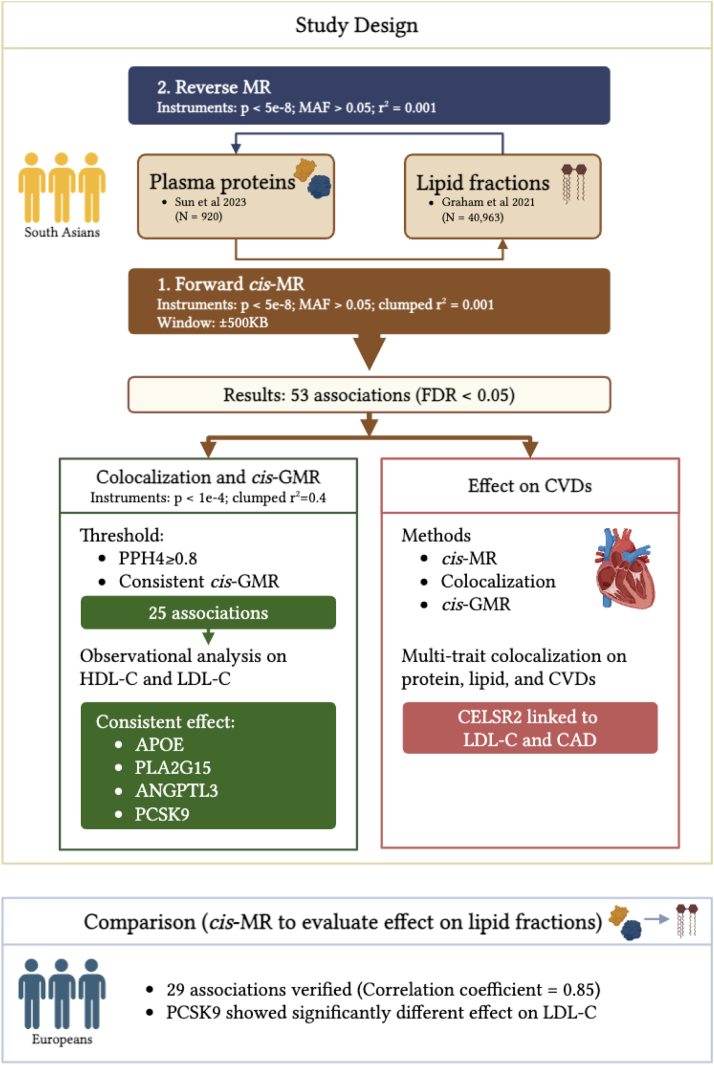
Overview of the study design. MR; mendelian randomisation, KB; kilobases, MAF; minor allele frequency, FDR; false discovery rate, GMR; generalised MR, PPH4; posterior probability of hypothesis 4, HDL-C and LDL-C; high- and low-density lipoprotein cholesterol (This figure was created using BioRender).

**Fig. 2:**
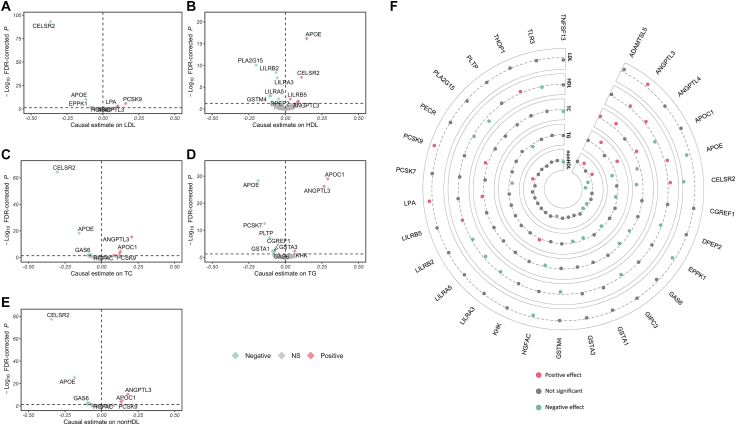
Volcano plots showing the causal effect of circulating plasma protein on the 5 lipid fractions (A) LDL-C; (B) HDL-C; (C) TC; (D) TG; (E) non-HDL-C. Each dot indicates a plasma protein with the x-axis showing the Wald ratio or IVW estimate while the y-axis showing -log_10_ FDR-corrected *P* from the MR analysis. (F) Circular plot showing the overlap of plasma proteins with the 5 lipid fractions tested. The dotted grey line represents the reference line for a null effect (i.e., an MR causal estimate of zero). The farther a dot is from this line, the stronger the estimated effect. Statistically significant associations are color-coded: red for positive effects (above the reference line) and green for negative effects (below the reference line).

An overview of the study design is shown in Fig. 1. Excluding proteins with ambiguous cognate genes and those encoded by genes located on the X-chromosome or within the *MHC* region (Supplementary Table S1), 2800 proteins were included in our study. Of these, 708 had at least one genetic IV available (Supplementary Fig. S1) and were carried forward for the proteome-wide MR study. IVs for each protein are presented in Supplementary Table S2. Using the Wald ratio (IV = 1) or IVW (IVs≥2) approach as the primary MR method, 186 plasma proteins showed a potential causal effect on at least one of the lipid fractions (309 associations in total; Prange = 6.0 × 10^−97^ to 0.05; Supplementary Table S3). After adjusting for multiple testing (5% FDR), a total of 29 genetically proxied plasma proteins showed potential causal effect on at least one of the 5 lipid fractions (53 associations in total; FDRrange = 4.3 × 10^−94^ to 0.05, Fig. 2, Supplementary Table S3). Out of the 53 associations with FDR < 0.05, 37 remain statistically significant using Bonferroni correction (Supplementary Table S3). Given the strong correlation between some plasma proteins, associations with FDR < 0.05 were considered statistically significant, and all downstream analyses were based on these FDR-corrected significant findings.

To avoid bias due to sample overlap between UKBPPP and GLGC, we performed MR using GLGC data without UKBB participants and the MR estimates were largely consistent across GLGC with and without UKBB (Pearson r^2^ = 0.93, *P* < 0.001; Supplementary Fig. S2A and B). Given the high Pearson correlation between the beta estimates, full GLGC data was carried forward for further downstream analyses.

Among these 29 lipids-associated proteins (53 associations), 12 (25 associations) were supported by strong colocalisation evidence (PPH4 > 80%) with one of the lipids fractions including ANGPTL3, APOE, CELSR2, EPPK1, GAS6, GSTA1, GSTA3, HGFAC, LPA, PCSK9, PLA2G15, and PLTP (Supplementary Table S4). Particularly, we identified strong colocalisation of ANGPTL3 with all lipid fractions except HDL-C, CELSR2 with all lipid fractions except TG, LPA with LDL-C and TC, and PCSK9 with HDL-C, TC, and non-HDL-C. Additionally, 7 plasma proteins (7 associations in total) showed suggestive colocalisation evidence (80% > PPH4 ≥ 60%) with the tested lipid fractions (Supplementary Fig. S3 and Table S4).

To further validate our findings in the proteome-wide *cis*-MR, we applied gIVW by including moderately significant (*P* < 1 × 10^−4^) and correlated (r^2^ < 0.4) cis-pQTLs as instruments.^34^ Out of the 53 associations of lipid fractions with plasma proteins, gIVW produced consistent and statistically significant estimates (Prange = 1.1 × 10^−28^ to 0.05) for 43 associations (Supplementary Table S5A). GSMR, provided as Supplementary analysis, produced consistent result as gIVW (Supplementary Table S5B). Subsequently, gEgger and weighted median were applied to the 42 associations with ≥ 3 SNP instruments. Weighted median produced highly consistent estimates for all 42 associations. The gEgger detected no horizontal pleiotropy (FDR corrected *P* for intercept > 0.05) but derived inconsistent estimates for the association of TNFSF13 with TC and non-HDL-C, THOP1 with HDL-C, and EPPK1 with TC (Supplementary Table S5A). Altogether, 30 associations of 14 genetically proxied proteins with lipid fractions were identified by proteome-wide MR, further validated by colocalisation (either strong or suggestive) and the subsequent MR analysis generalised to correlated instruments (GMR). The top findings include CELSR2 associated with all lipid fractions except TG, PCSK9 and HGFAC with TC, LDL-C, and non-HDL-C, LPA with LDL-C and TC, and ANGPTL3 with all lipid fractions (Fig. 2).

### Potential causal effects of lipid-associated proteins on CVD outcomes

**Fig. 3:**
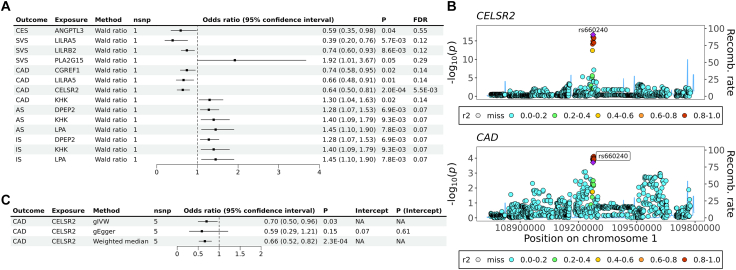
Effect of lipid-associated plasma proteins on CVDs. A) Forest plot showing all plasma proteins that were nominally associated with CVDs (*P* < 0.05). B) Stacked genomic locus plot showing evidence for colocalisation between CELSR2 and CAD; and C) Forest plot showing the effect of CELSR2 on CAD estimated by gIVW, gEgger, and weighted median.

Subsequently, we investigated whether lipid-associated proteins (N = 29) identified by proteome-wide *cis-*MR have potential causal effects on the risk of CAD and stroke in South Asians. After correcting for multiple testing (5% FDR), only genetically predicted CELSR2 had a causal association with CAD (Odds ratio [OR] = 0.64, 95% CI = 0.50, 0.81, FDR = 0.006; Fig. 3A, Supplementary Table S6) which was also supported by strong colocalisation evidence (PPH4 = 93.6%, Fig. 3B, Supplementary Table S7). Additionally, gIVW, gEgger, and weighted median produced consistent estimates, and no pleiotropy was detected (Fig. 3C, Supplementary Table S8). Notably, LPA showed suggestive associations with CVDs (*P* = 7.8 × 10^−3^ for LPA with all types of strokes) but did not pass the 5% FDR threshold (Fig. 3A).

We next assessed the causal effects of five lipid fractions on CVDs. Our univariable MR identified four associations reaching nominal significance (Prange = 0.02 to 0.05), including LDL-C with CAD (β_IVW_ = 1.64; 95% CI = 1.03, 2.62) and cardioembolic stroke (β_IVW_ = 1.78; 95% CI = 1.01, 3.15); Supplementary Table S9). The MR-Egger and weighted median methods produced estimates consistent in direction with the inverse variance weighted method.

**Fig. 4:**
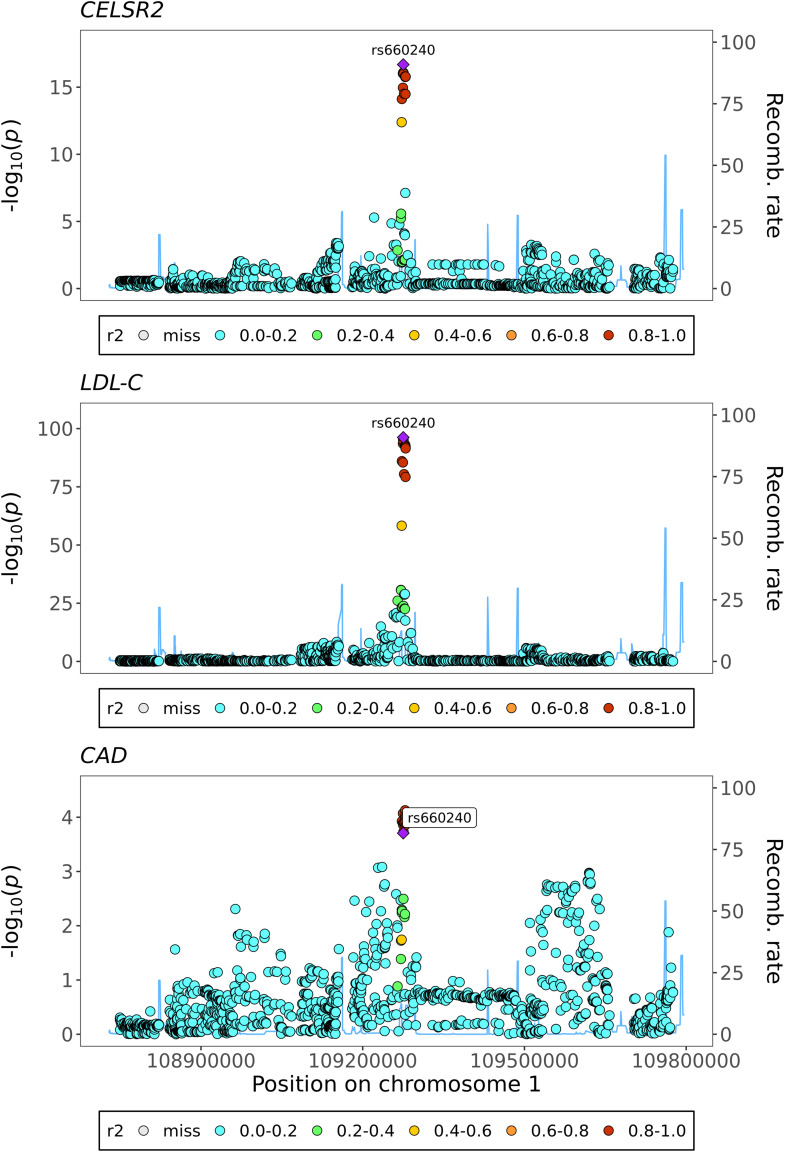
Stacked regional genomic plot from multi-trait colocalisation showing the colocalised genetic variant rs660240 across LDL-C, CAD at the *CELSR2* locus in South Asians.

Since CELSR2 showed potential causal effects on both LDL-C and CAD while genetically proxied LDL-C was also causally associated with CAD risk, a multi-trait colocalisation was performed on the traits in the genomic region ± 500 KB extended from the *CELSR2* gene. The multi-trait colocalisation produced a posterior probability of 70.0% that CELSR2, LDL-C, and CAD colocalised in this region (Fig. 4, Supplementary Table S10). The posterior probabilities for all 15 scenarios of multi-trait colocalisation were presented in Supplementary Table S10.

### Reverse MR with lipid fractions as exposures and plasma proteins as outcomes

To understand the plasma proteins modified by the lipid fractions, a reverse MR was performed using lipid fractions as exposures and plasma proteins as outcomes. This analysis identified genetically proxied associations between TG and LDLR, and HDL-C with 3 proteins: APOA1, MENT, and FGFBP2 (β_range_ = 0.55 to 0.61, FDR-corrected P_range_ = 0.05 to 0.02, Supplementary Fig. S4 and Table S11). Subsequent MR-Egger and weighted median produced consistent estimates and detected no horizontal pleiotropy (Supplementary Fig. S5 and Table S12).

### Comparison of MR findings across South Asians and Europeans

Among the 53 proteome-wide MR identified associations in South Asians, 29 were statistically significant (FDR-corrected Prange = 7.8 × 10^−9^ to 2.2 × 10^−308^) and consistent (i.e., beta estimates of the same sign) in Europeans. The correlation analysis on the 29 beta estimates in South Asians against those in European produced a correlation coefficient of 0.84 (*P* = 8.3 × 10^−9^, Supplementary Fig. S6).

**Fig. 5:**
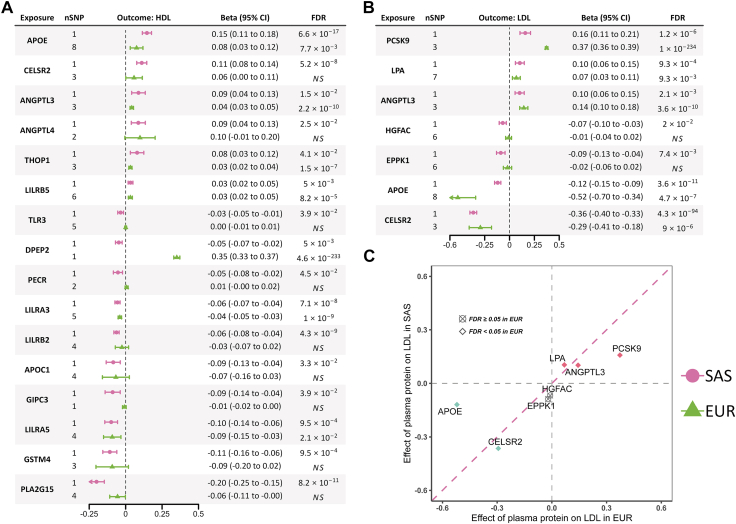
Effect of plasma proteins on lipid fractions using *cis-*MR in South Asians and Europeans on (A) HDL-C; (B) LDL-C; and (C) Scatter plot for comparison of causal effect estimates from MR between Europeans and South Asians for LDL-C.

Restricting the 53 South Asians MR findings to 30 associations that has strong/suggestive colocalisation and GMR evidence, 22 were verified in Europeans (Supplementary Table S13 and Fig. 5). Briefly, proteome-wide MR, colocalisation, and GMR identified 6 proteins associated with HDL-C in South Asians, but only ANGPTL3 and APOE were also statistically significant in Europeans (Fig. 5). We observed that DPEP2 showed statistically significant but discordant direction of effect on HDL-C in South Asians vs. Europeans. However, this protein did not show any evidence of Bayesian colocalisation (PPH4 = 2.0 × 10^−7^, Supplementary Table S4). Of the 6 proteins linked to LDL-C in South Asians, ANGPTL3, CELSR2, LPA, and PCSK9 were verified in Europeans, with PCSK9 showing a stronger effect in Europeans (βEuropean = 0.37; 95% CI = 0.36, 0.38; βSouth Asians = 0.16; 95% CI = 0.11, 0.21); Fig. 5). Among the remaining 18 associations with non-HDL-C, TC, or TG, only the association of EPPK1 with non-HDL-C and HGFAC with TC were not statistically significant in Europeans.

### Observational associations of protein levels with lipid fractions

There were three proteins with strong evidence (MR, strong colocalisation, and GMR) of association with HDL-C (APOE, CELSR2, and PLA2G15) and 6 proteins with LDL-C (ANGPTL3, CELSR2, EPPK1, HGFAC, LPA, and PCSK9) that were tested in observational analysis. After adjusting for multiple testing, all 9 proteins had a statistically significant association with their genetically associated lipid fraction (FDR adjusted Prange = 2.0 × 10^−16^ to 2.2 × 10^−308^) but only 4 had a consistent direction of effect with MR estimates (Supplementary Table S14); APOE (β = 0.078; 95% CI = 0.075, 0.082) and PLA2G15 (β = −0.067; 95% CI = −0.071, −0.063) with HDL-C, and ANGPTL3 (β = 0.180; 95% CI = 0.175, 0.184) and PCSK9 (β = 0.196; 95% CI = 0.192, 0.200) with LDL-C. Out of these four proteins, there was a statistically significant interaction between ANGPTL3 and PCSK9 levels with LDL-C in South Asians with South Asians having a significantly lower effect compared to other ancestries (ANGPLT3∗South Asian; β = −0.072; 95% CI = −0.103, −0.040, PCSK9∗South Asian; β = −0.140; 95% CI = −0.174, −0.107).

## Discussion

In the current study, we performed a bidirectional proteome-wide MR on five lipid fractions, and linked lipid-related proteins to cardiovascular outcomes in South Asians. Our study confirmed key proteins (PCSK9, ANGPTL3, LPA), identified potential novel targets (GSTA1, GSTA3, EPPK1, PECR, and PLA2G15), and strengthened evidence for CELSR2 and GAS6 in dyslipidaemia. Notably, our results highlight significant heterogeneity in MR estimates across genetic ancestry groups, particularly for the effect of PCSK9 on LDL-C. We also report CELSR2 with evidence for its effect on LDL-C and CAD risk. Reverse MR identified LDLR as modifiable by TG, and APOA1, MENT, and FGFBP2 by HDL-C.

We found an inverse association of genetically proxied CELSR2 with LDL-C and CAD risk in South Asians. CELSR2 is a transmembrane protein belonging to the flamingo family of cadherin superfamily.^40^ Although the biological function of CELSR2 is not well understood, its role in lipid metabolism has been suggested by previous studies. A locus in the vicinity of *CELSR2*, rs599839 (in LD with rs660240, the genetic instrument for CELSR2 in this study [r^2^ = 0.989 in South Asians, r^2^ = 0.871 in Europeans]), was first reported to be associated with CAD, LDL-C and TC by two GWAS conducted in European ancestry.^41^, ^42^, ^43^ rs660240, a 3' UTR variant, is an eQTL for *CELSR2*, *PSRC1*, and *SORT1* in liver tissue (Open Target Genetics). It shows slight variations in allele frequencies between South Asians (MAF = 0.26), Europeans (MAF = 0.20), and East Asians (MAF = 0.04), which could have implications for studies related to disease susceptibility and treatment response. Furthermore, a transcriptomic study revealed the risk allele of rs599839 to CAD and high LDL-C also suppressed the expression of *CELSR2* gene in liver.^44^ Extending to non-European populations, the association of *CELSR2* variants with lipid fractions and CAD risk was also verified in the South Asian population. However, although the effect of *CELRS2* on lipid metabolism was indicated by genetic and transcriptomic studies, the underlying mechanism is less clear. One study demonstrated that CELSR2 deficiency can elevate reactive oxygen species of hepatocytes, which impairs lipid homeostasis and physiological unfolded protein response.^45^ Our findings are consistent with the significant role of CELSR2 in CAD and lipid metabolism as suggested by earlier studies and extends the potential generalisability of CELSR2’s potential causal role in lipids to the South Asian population.

We identified an inverse association of genetically proxied GAS6 with TC and TG. GAS6 is a ligand for TAM receptor protein tyrosine kinases including AXL, TYRO3 and MER. The GAS6 - TAM pathway was found implicated in carcinoma, inflammation, and haemostasis and has been targeted for the treatment of carcinoma.^46^, ^47^, ^48^ In recent years, there is also evidence that GAS6 plays a role in regulating obesity and lipid metabolism as plasma gamma-glutamyl carboxylated GAS6 (Gla-GAS6) was found significantly lower in hyperlipidaemic individuals compared with healthy controls.^49^ The subsequent experiment showed that higher Gla-GAS6 expression induced by vitamin K in plasma and hepatocyte could reduce the plasma lipid level in hyperlipidaemic mice.^49^ The Gla-GAS6 takes effect by regulating the AMPK/SREBP1/PPARα signalling pathways of hepatic lipid metabolism.^49^ We further strengthen the evidence for the role of GAS6 in lipid metabolism by providing strong evidence from proteome-wide MR, which suggests that GAS6 could potentially be causally associated with different lipid fractions.

In this study, we replicated previously established protein associations with lipid metabolism, including ANGPTL3, APOE, LPA, PCSK9, and PLTP. A summary of the main findings (e.g., the prioritised proteins, lipid fraction affected, relevant pathways, novelty) is presented in Supplementary Table S15. Briefly, APOE and LPA are components of LDL-C while ANGPTL3 and PCSK9 are known drug targets for dyslipidaemia treatment.^50^ PLTP, phospholipid transfer protein, is also implicated in lipid metabolism but the mechanism by which it may affect lipid fractions is still under investigation. PLTP is a known mediator in the process of transferring phospholipids and free cholesterol from triglyceride-rich lipoproteins (primarily LDL-C and very - low density lipoprotein cholesterol, VLDL-C) into HDL-C.^51^ In addition, it also enhances the clearance of cholesterol from peripheral cells and tissues.^52^

To highlight, despite the concordance in direction and significance for the association of PCSK9 on LDL-C in both South Asians and Europeans, we identified a significantly reduced effect in South Asians as supported by both MR and observational analysis. PCSK9 is a protein that can bind to and degrade LDL receptor family members on hepatocytes, which are responsible for removing LDL-C from circulation.^53^ Therefore, inhibiting PCSK9 could potentially lower plasma LDL-C level and protect against CVDs. Since drug responses to lipid-lowering therapy may vary across ethnicities, this finding may have important clinical implications. Previous studies suggest that atorvastatin and simvastatin have similar lipid-lowering effects in South Asians patients compared to those in European.^54^ In an open-label, phase 4 study in India, homozygous familial hypercholesterolemia patients receiving monthly 420 mg evolocumab achieved a 6.4% reduction in LDL-C at week 12.^55^ In contrast, a phase 3 randomised, placebo-controlled trial in European patients with familial hypercholesterolemia showed a 30.9% reduction in LDL-C with the same dose of evolocumab over 12 weeks.^56^ Despite differences in study design and baseline characteristics, these results potentially suggest that PCSK9 inhibition may have a weaker effect on LDL-C levels in South Asian populations. Therefore, PCSK9, as a target for lipid-lowering medication, warrants further investigation for their effect in more diverse ethnicity. Mechanisms accounting for the ancestral heterogeneity in PCSK9’s effect are limited. However, a recent study sequencing *PCSK9* gene in Indians indicated difference in prevalence of mutation in South Asians. The by-ancestry heterogeneous mutation pattern can result in heterogeneity of PCSK9 structure and activity, which may modify the effect of PCSK9 abundance.^57^

Additionally, our study identified potentially novel associations, including HGFAC, GIPC3, EPPK1, GSTA1, GSTA3, PECR, and PLA2G15. HGFAC activates hepatocyte growth factor (HGF) by converting it to a heterodimer, which then binds to the MET receptor to activate downstream signalling. A previous study linked a putative *HGFAC* loss-of-function variant to elevated serum TG and LDL-C.^58^ In another animal experiment, an increase of circulating TG was present in both male and female HGFAC-KO mice while higher level of circulating TC was present in male HGFAC-KO mice.^59^ PLA2G15 (LCAT-like lysophospholipase, LLPL) is a major lysosomal phospholipase recently linked to HDL-C in a GWAS of British South Asians.^60^ A prior Pla2g15−/− knockout study also reported its presence in foam cells within atherosclerotic lesions in apoE−/− mice,^61^ suggesting a role in atherosclerosis. Together with our findings, these results highlight a potential role for LLPL in lipid regulation and atherosclerosis. The association of GIPC3 with HDL-C is potentially novel and previous studies have shown that plasma protein levels of GIPC3 are associated with the Framingham risk score.^62^ Lastly, we also identified GSTA1, GSTA3, EPPK1, and PECR associated with lipid traits, all of which are potential novel findings.

Conversely, we applied MR to identify plasma proteins modified by lipid fractions. We found that genetically proxied TG levels were associated with increased plasma LDLR. LDLR is a cell membrane glycoprotein that regulates lipid homeostasis by binding and internalising circulating cholesterol-containing lipoprotein particles, including LDL-C, VLDL-C, and chylomicron remnants.^63^ Deficiency in LDLR can result in dyslipidaemia. Therefore, we interpreted that the LDLR upregulation triggered by genetically proxied TG is likely to reverse the hyperlipidaemia. In addition, we observed genetically proxied HDL-C level associated with increased plasma APOA1, MENT, and FGFBP2 level. All three proteins play important roles in various disorders and mechanisms, including cholesterol transport, angiogenesis, tissue repair, and cellular metabolism. Therefore, further investigation is necessary for detailed biological interpretation of these findings.

Our study has several strengths. First, to the best of our knowledge, this is the first MR study to systematically evaluate the potential causal association between plasma proteins and lipid traits in South Asians, replicating known protein-lipids and CVD associations and discovering potential novel targets. Secondly, we combined MR with colocalisation, to reduce bias from LD and reverse causation — a potential limitation of conventional observational studies. Thirdly, MR approach, as a genetically proxied approach, is less affected by environment factors. Therefore, although genetic associations of plasma proteins and CAD were measured in British South Asians, our MR findings may be generalisable to population residing in South Asian continent as long as British South Asian in UK biobank is representative of the entire South Asian population. However, leaving environment factors out of consideration can lead to false negatives, especially when gene-environment interaction arises. Additionally, whether British South Asian in the UK biobank is representative of people living in South Asia is not yet investigated. Therefore, our findings from British South Asians need to be interpreted cautiously and future studies with larger sample size will be required to validate our findings. Finally, by incorporating GWAS on CVDs, we linked lipid associated proteins to cardiovascular outcomes and identified CELSR2 as a promising target for both LDL-C and CAD. Given CELSR2 is a transmembrane receptor, and our study advised that CELSR2 abundance is inversely associated with plasma LDL-C level and can protect against CAD, activating CELSR2 could hold promise for treating dyslipidaemia and CAD.

There are also limitations in our study. First, the genetic associations for plasma proteins in South Asians were based on a relatively small sample size, compared with current GWAS standards. Furthermore, the smaller GWAS sample size in South Asians compared to Europeans may introduce bias in detecting associations, and proteins with modest effect sizes may have been missed due to the relatively limited power of the South Asian proteomics GWAS. Secondly, as most proteins were instrumented by fewer genetic variants, the potential causal effects estimated using MR-Egger and weighted median methods may be biased and may not be suitable for testing horizontal pleiotropy (using MR-Egger); therefore, such findings warrant careful interpretation. Thirdly, in GMR analysis, we further included weaker instruments (*P* < 5 × 10^−6^ and *P* < 1 × 10^−4^). Although all included genetic instruments had F-statistics ≥ 10, weak instrument bias cannot be dismissed particularly given the lowest F-statistics was 15.15, where the genetically proxied estimates may still be biased towards the confounded, observational association.^64^ Fourthly, to avoid horizontal pleiotropy, we used cis-pQTLs as genetic instruments and applied various MR methods and sensitivity analyses including Bayesian colocalisation, gIVW and gEgger. However, unaccounted pleiotropy may still bias our results. Furthermore, our analysis assumes the absence of SNP–SNP and SNP–environment interactions, as such interactions were not measured in the GWAS. However, ignoring these interactions may lead to false negatives, potentially missing genetic variants that are effective only under specific conditions.^65^^,^^66^ Moreover, participation bias is known in UKBB and using proteomic GWAS measured in UKBB participants can potentially distort the genetic associations and bias the MR estimate by violating the assumption that genetic instruments should be independent of the confounders.^67^ Moreover, the genetic background of British South Asians in the UK Biobank may not be representative of all South Asian populations. Therefore, our findings may not be generalisable to individuals living in the South Asian subcontinent. Further studies are needed to validate the prioritised associations in South Asians residing in South Asia. We also noted that our genetically proxied findings may not be generalisable to ancestry groups (e.g., Black African, Hispanics, and East Asian) other than the ones studied here. Lastly, although our study advised serval new proteins associated with lipid fractions, the mechanisms underlying the association is unknown while functional experimental studies are warranted to elucidate the pathways linking the association.

Our comprehensive study triangulated evidence from MR, colocalisation, and observational analyses, highlighting several potential novel proteins associated with lipid fractions in South Asians. Notably, our analysis suggests that the causal effect of PCSK9 on LDL-C may be ancestry-specific. Future studies with larger sample sizes are needed to validate our findings, along with further mechanistic and clinical studies to confirm the role of PCSK9 on LDL-C in South Asians and Europeans.

## Contributors

DM, IT, and AD contributed to the conception and design of the study. SW and AS contributed to acquisition and statistical analysis of data. DM, IT, AD supervised the project. All authors contributed to the drafting and critical revision of the manuscript.

## Data sharing statement

The UKBPPP data can be downloaded from http://ukb-ppp.gwas.eu. GLGC dataset can be downloaded from https://csg.sph.umich.edu/willer/public/glgc-lipids2021/results/ancestry_specific/. ELGH data can be downloaded from https://www.genesandhealth.org/research/scientific-data-downloads/gwas-data-genes-health-feb-2020-datafreeze. GWAS summary statistics for stroke and its subtypes for South Asians and Europeans can be obtained from the GWAS catalogue with accession GCST90104559-GCST90104563, and GCST90104539-GCST90104543, respectively.

## Declaration of interests

AR reported consultancies for Raydel, Johnson & Johnson, and JP Morgan (all with modest compensation). AR also received a significant research grant from CSL Limited, but this did not include any salary support. We declare no other competing interests.
