# Supplementary material for: Identification of protein targets for dyslipidaemia and cardiovascular diseases among people with South Asian ancestry: a mendelian randomisation study
Source: Lancet Reg Health Southeast Asia. 2025 Jul 4;39:100621. doi: 10.1016/j.lansea.2025.100621 (PMC12271078; doi:10.1016/j.lansea.2025.100621)
Supplement: Supplementary Figs. S1–S6 and Tables S1–S15 [file mmc1.docx]

**SUPPLEMENTARY INFORMATION**

**Identification of protein targets for dyslipidaemia and cardiovascular diseases among people with South Asian ancestry: a mendelian randomisation study**

*Siwei Wu^a,b‡^, Devendra Meena^b‡^*, Alexander Smith^b‡^, Jingxian Huang^b^, Georg W. Otto^b^, Yi-Hsuan C^b^, James Yarmolinsky^b^, Dipender Gill^b^, Anand Rohatgi^c^, Abbas Dehghan^b,d^, Ioanna Tzoulaki^b^*

*The Lancet Regional Health—Southeast Asia* 2025

**Contents**

[Supplementary Method: Ancestral background of participants in each GWAS 3](#_Toc199454285)

[Supplementary Method: instrument selection for proteome-wide Mendelian randomisation 3](#_Toc199454286)

[Supplementary Method: Software and packages 3](#_Toc199454287)

[Supplementary Table S1. Proteins excluded from the proteome-wide Mendelian randomisation 4](#_Toc199454288)

[Supplementary Table S2. Instrument variables for proteins associated with lipid fractions at P value ≤ 0.05 8](#_Toc199454289)

[Supplementary Table S3. Proteome-wide MR results for associations with P ≤ 0.05 22](#_Toc199454290)

[Supplementary Table S4. Results of Bayesian colocalisation 35](#_Toc199454291)

[Supplementary Table S5A. Result of Generalised Mendelian randomizsation 38](#_Toc199454292)

[Supplementary Table S5B. Result of GSMR 46](#_Toc199454293)

[Supplementary Table S6. MR estimates of plasma proteins’ effect on cardiovascular diseases 49](#_Toc199454294)

[Supplementary Table S7. Colocalisation between plasma proteins and cardiovascular diseases 52](#_Toc199454295)

[Supplementary Table S8. Generalised Mendelian randomisation result of CELSR2’s effect on CAD 53](#_Toc199454296)

[Supplementary Table S9. Mendelian randomisation result of lipid fractions’ effect on cardiovascular diseases 53](#_Toc199454297)

[Supplementary Table S10. Multi-trait colocalisation between CELSR2, LDL-C, and CAD 55](#_Toc199454298)

[Supplementary Table S11. Significant Mendelian randomisation result of lipid fractions’ effect on plasma protein 56](#_Toc199454299)

[Supplementary Table S12. MR-Egger and weighted median result of lipid fractions’ effect on plasma protein 56](#_Toc199454300)

[Supplementary Table S13. Mendelian randomisation result of plasma proteins’ effect on lipid fractions in European 57](#_Toc199454301)

[Supplementary Table S14. Observational associations of plasma protein levels with lipid fractions 59](#_Toc199454302)

[Supplementary Table S15. Summary table of plasma proteins associated with lipid fractions 61](#_Toc199454303)

[Supplementary Figure S1. Number of instruments for plasma proteins 64](#_Toc199454304)

[Supplementary Figure S2A. Scatter plot comparing proteome-wide MR result including or excluding UK Biobank data in GWAS on lipid fractions 65](#_Toc199454305)

[Supplementary Figure S2B. Volcano plot comparing proteome-wide MR result including or excluding UK Biobank data in GWAS on lipid fractions 66](#_Toc199454306)

[Supplementary Figure S3A. Regional genomic plot for colocalisation between plasma proteins and LDL-C 67](#_Toc199454307)

[Supplementary Figure S3B. Regional genomic plot for colocalisation between plasma proteins and HDL-C 68](#_Toc199454308)

[Supplementary Figure S3C. Regional genomic plot for colocalisation between plasma proteins and TC 69](#_Toc199454309)

[Supplementary Figure S3D. Regional genomic plot for colocalisation between plasma proteins and TG 70](#_Toc199454310)

[Supplementary Figure S3E. Regional genomic plot for colocalisation between plasma proteins and nonHDL-C 71](#_Toc199454311)

[Supplementary Figure S4. Volocanoe plot of reverse MR evaluating lipid fractions’ effect on plasma protein 72](#_Toc199454312)

[Supplementary Figure S5. Forest plot of reverse MR identified plasma proteins effected by lipid fractions 73](#_Toc199454313)

[Supplementary Figure S6. Scatter plot comparing proteome-wide MR estimates in South Asian versus European 74](#_Toc199454314)

[Reference 75](#_Toc199454315)

# **Supplementary Method: Ancestral background of participants in each GWAS**

All the UKBPPP participants were citizens of the United Kingdom, among which 920 individuals are of Central/South Asian ancestry. The ancestry in UKBPPP referred to the genetic ancestry in reference to 2 large external reference dataset, 1000 Genomes Project and Human Genome Diversity Project (HGDP) ^1,2^. Given Central / South Asian genetic ancestry was essentially defined by individuals of Pakistan, Indian, Bangladesh, and Sri Landan ancestry in reference genome, we simply referred to the Central / South Asian ancestry as South Asian ancestry in this study. The European ethnicity of the 34,577 participants in UKBPPP refers to genetic ancestry as well. Similarly, the ELGH study, where the genetic associations of CAD were obtained for South Asian ancestral population, recruited British Bangladeshi or British Pakistani people in London ^3^.

However, the GLGC includes South Asian ancestral people living in India, Pakistan, Sri Lanka, and the UK while South Asian samples in GIGASTROKE study are from India or Pakistan ^4^. The European ancestral population in GLGC also involves participants from all over the world including Europe, North America, and Australia. Similarly, the CARDIoGRAMplusC4D and GIGASTROKE Consortium included European ethnicity people from diverse regions ^4,5^.

# **Supplementary Method: instrument selection for proteome-wide Mendelian randomisation**

To obtain genetic instruments for plasma proteins, we selected instrument variables from cis-protein quantitative trait locus (cis-pQTLs). We defined cis-pQTLs as genetic variants associated with plasma protein abundance that lies with +/- 500 kilobase (KB) from the coding gene. From the cis-pQTLs, biallelic single nucleotide polymorphisms (SNP), with minor allele frequency (MAF) > 0.05, and reaching genome wide significant level (P < 5 × 10^-8^) were extracted. The SNPs were further harmonized to the 5 lipid fractions and clumped to an r^2^ < 0.001 (in reference to 1000 Genome ancestry specific reference panel) to ensure independence across the instruments ^1^. Subsequently, a F statistic was calculated for each SNP and SNPs with F-statistic < 10 were excluded to avoid weak instrument bias ^6^. We also applied the Steiger filtering and excluded the SNPs with potential reverse causality ^7^.

# **Supplementary Method: Software and packages**

All statistical analyses were conducted with R 4.4.2. IVW, MR-Egger, and weighted median were performed with Package TwoSampleMR (version 0.6.9). GMR analysis including gIVW and gEgger were conducted with MendelianRandomization package (version 0.10.0). GSMR was performed using the gsmr package (version 1.1.1). We used coloc package (version 5.2.3) for Bayesian colocalisation and moloc package (version 0.1.0) for multi-trait colocalisation.

# **Supplementary Table S1. Proteins excluded from the proteome-wide Mendelian randomisation**

| **Protein** | **UniProtID** | **ProbeID** | **Coding gene ambiguious** | **Coding gene on Chromosome X** | **Coding gene on MHC region** |
| --- | --- | --- | --- | --- | --- |
| ACE2 | Q9BYF1 | OID20105 | FALSE | TRUE | FALSE |
| ADGRG2 | Q8IZP9 | OID20189 | FALSE | TRUE | FALSE |
| AGER | Q15109 | OID20756 | FALSE | FALSE | TRUE |
| AIF1 | P55008 | OID21234 | FALSE | FALSE | TRUE |
| AIFM1 | O95831 | OID21287 | FALSE | TRUE | FALSE |
| APOM | O95445 | OID20374 | FALSE | FALSE | TRUE |
| ATG4A | Q8WYN0 | OID21246 | FALSE | TRUE | FALSE |
| ATP6AP2 | O75787 | OID21178 | FALSE | TRUE | FALSE |
| BAG6 | P46379 | OID20114 | FALSE | FALSE | TRUE |
| BGN | P21810 | OID21198 | FALSE | TRUE | FALSE |
| C2 | P06681 | OID20410 | FALSE | FALSE | TRUE |
| CD40LG | P29965 | OID20616 | FALSE | TRUE | FALSE |
| CD99 | P14209 | OID21110 | FALSE | TRUE | FALSE |
| CD99L2 | Q8TCZ2 | OID21122 | FALSE | TRUE | FALSE |
| CDSN | Q15517 | OID20667 | FALSE | FALSE | TRUE |
| CETN2 | P41208 | OID20836 | FALSE | TRUE | FALSE |
| CHRDL1 | Q9BU40 | OID20771 | FALSE | TRUE | FALSE |
| CKMT1A_CKMT1B | P12532 | OID20721 | TRUE | FALSE | FALSE |
| CSF2RA | P15509 | OID20915 | FALSE | TRUE | FALSE |
| DDR1 | Q08345 | OID21009 | FALSE | FALSE | TRUE |
| DEFA1_DEFA1B | P59665 | OID20344 | TRUE | FALSE | FALSE |
| DEFB4A_DEFB4B | O15263 | OID21373 | TRUE | FALSE | FALSE |
| EBI3_IL27 | Q14213_Q8NEV9 | OID21389 | TRUE | FALSE | FALSE |
| EDA2R | Q9HAV5 | OID21451 | FALSE | TRUE | FALSE |
| F9 | P00740 | OID20349 | FALSE | TRUE | FALSE |
| FMR1 | Q06787 | OID21233 | FALSE | TRUE | FALSE |
| FUT3_FUT5 | P21217_Q11128 | OID21013 | TRUE | FALSE | FALSE |
| GPKOW | Q92917 | OID20890 | FALSE | TRUE | FALSE |
| HLA-DRA | P01903 | OID20520 | FALSE | FALSE | TRUE |
| HLA-E | P13747 | OID20532 | FALSE | FALSE | TRUE |
| HSPA1A | P0DMV8 | OID20718 | FALSE | FALSE | TRUE |
| IDS | P22304 | OID20619 | FALSE | TRUE | FALSE |
| IKBKG | Q9Y6K9 | OID20544 | FALSE | TRUE | FALSE |
| IL12A_IL12B | P29459_P29460 | OID21327 | TRUE | FALSE | FALSE |
| IL13RA1 | P78552 | OID21504 | FALSE | TRUE | FALSE |
| IL3RA | P26951 | OID20461 | FALSE | TRUE | FALSE |
| IRAK1 | P51617 | OID20485 | FALSE | TRUE | FALSE |
| ITGB1BP2 | Q9UKP3 | OID20112 | FALSE | TRUE | FALSE |
| ITM2A | O43736 | OID20504 | FALSE | TRUE | FALSE |
| L1CAM | P32004 | OID21484 | FALSE | TRUE | FALSE |
| LAMP2 | P13473 | OID21035 | FALSE | TRUE | FALSE |
| LGALS7_LGALS7B | P47929 | OID21406 | TRUE | FALSE | FALSE |
| LTA | P01374 | OID20586 | FALSE | FALSE | TRUE |
| MAGED1 | Q9Y5V3 | OID21181 | FALSE | TRUE | FALSE |
| MICB_MICA | Q29980_Q29983 | OID20593 | TRUE | FALSE | TRUE |
| MOG | Q16653 | OID21384 | FALSE | FALSE | TRUE |
| MPIG6B | O95866 | OID20779 | FALSE | FALSE | TRUE |
| PLXNB3 | Q9ULL4 | OID20160 | FALSE | TRUE | FALSE |
| PQBP1 | O60828 | OID21172 | FALSE | TRUE | FALSE |
| RP2 | O75695 | OID21302 | FALSE | TRUE | FALSE |
| SH2D1A | O60880 | OID20514 | FALSE | TRUE | FALSE |
| SLITRK2 | Q9H156 | OID21409 | FALSE | TRUE | FALSE |
| TBL1X | O60907 | OID21220 | FALSE | TRUE | FALSE |
| TIMP1 | P01033 | OID20418 | FALSE | TRUE | FALSE |
| TNF | P01375 | OID20074 | FALSE | FALSE | TRUE |
| TNF | P01375 | OID20473 | FALSE | FALSE | TRUE |
| TNF | P01375 | OID20848 | FALSE | FALSE | TRUE |
| TNF | P01375 | OID21237 | FALSE | FALSE | TRUE |
| TNXB | P22105 | OID21107 | FALSE | FALSE | TRUE |
| VEGFD | O43915 | OID20662 | FALSE | TRUE | FALSE |
| VSIG4 | Q9Y279 | OID21144 | FALSE | TRUE | FALSE |
| WAS | P42768 | OID20479 | FALSE | TRUE | FALSE |
| XG | P55808 | OID20259 | FALSE | TRUE | FALSE |
| XPNPEP2 | O43895 | OID21508 | FALSE | TRUE | FALSE |
| AMOT | Q4VCS5 | OID30572 | FALSE | TRUE | FALSE |
| AMY1A_AMY1B_AMY1C | P0DUB6_P0DTE7_P0DTE8 | OID30707 | TRUE | FALSE | FALSE |
| ARAF | P10398 | OID31260 | FALSE | TRUE | FALSE |
| ARMCX2 | Q7L311 | OID30792 | FALSE | TRUE | FALSE |
| ATP1B4 | Q9UN42 | OID31230 | FALSE | TRUE | FALSE |
| ATP6V1G2 | O95670 | OID30167 | FALSE | FALSE | TRUE |
| BEX3 | Q00994 | OID31202 | FALSE | TRUE | FALSE |
| BOLA2_BOLA2B | Q9H3K6 | OID30267 | TRUE | FALSE | FALSE |
| BRD2 | P25440 | OID30826 | FALSE | FALSE | TRUE |
| C1GALT1C1 | Q96EU7 | OID30180 | FALSE | TRUE | FALSE |
| CDKL5 | O76039 | OID30796 | FALSE | TRUE | FALSE |
| CFB | P00751 | OID30755 | FALSE | FALSE | TRUE |
| CFP | P27918 | OID30784 | FALSE | TRUE | FALSE |
| CGB3_CGB5_CGB8 | P0DN86 | OID30671 | TRUE | FALSE | FALSE |
| CHM | P24386 | OID30972 | FALSE | TRUE | FALSE |
| CTAG1A_CTAG1B | P78358 | OID31299 | TRUE | TRUE | FALSE |
| DDX53 | Q86TM3 | OID30944 | FALSE | TRUE | FALSE |
| DEFB103A_DEFB103B | P81534 | OID30534 | TRUE | FALSE | FALSE |
| DEFB104A_DEFB104B | Q8WTQ1 | OID30849 | TRUE | FALSE | FALSE |
| DIPK2B | Q9H7Y0 | OID30582 | FALSE | TRUE | FALSE |
| DMD | P11532 | OID30223 | FALSE | TRUE | FALSE |
| DXO | O77932 | OID31080 | FALSE | FALSE | TRUE |
| DYNLT3 | P51808 | OID31415 | FALSE | TRUE | FALSE |
| EIF1AX | P47813 | OID31125 | FALSE | TRUE | FALSE |
| ENOX2 | Q16206 | OID30152 | FALSE | TRUE | FALSE |
| FGF16 | O43320 | OID30535 | FALSE | TRUE | FALSE |
| FKBPL | Q9UIM3 | OID31499 | FALSE | FALSE | TRUE |
| FRMD7 | Q6ZUT3 | OID30898 | FALSE | TRUE | FALSE |
| GAGE2A | Q6NT46 | OID31199 | FALSE | TRUE | FALSE |
| GLA | P06280 | OID30594 | FALSE | TRUE | FALSE |
| GPR101 | Q96P66 | OID30819 | FALSE | TRUE | FALSE |
| H2AP | O75409 | OID31232 | FALSE | TRUE | FALSE |
| HCG22 | E2RYF7 | OID30088 | FALSE | FALSE | TRUE |
| HDAC8 | Q9BY41 | OID30436 | FALSE | TRUE | FALSE |
| HEPH | Q9BQS7 | OID30337 | FALSE | TRUE | FALSE |
| HLA-A | P04439 | OID31048 | FALSE | FALSE | TRUE |
| HS6ST2 | Q96MM7 | OID30481 | FALSE | TRUE | FALSE |
| IGBP1 | P78318 | OID31059 | FALSE | TRUE | FALSE |
| IL13RA2 | Q14627 | OID30841 | FALSE | TRUE | FALSE |
| IL2RG | P31785 | OID31361 | FALSE | TRUE | FALSE |
| LTB | Q06643 | OID31383 | FALSE | FALSE | TRUE |
| MAGEA3 | P43357 | OID31331 | FALSE | TRUE | FALSE |
| MCTS1 | Q9ULC4 | OID31444 | FALSE | TRUE | FALSE |
| MORF4L2 | Q15014 | OID31160 | FALSE | TRUE | FALSE |
| MUCL3 | Q3MIW9 | OID31170 | FALSE | FALSE | TRUE |
| NAA10 | P41227 | OID30188 | FALSE | TRUE | FALSE |
| NUDT10 | Q8NFP7 | OID30198 | FALSE | TRUE | FALSE |
| OFD1 | O75665 | OID31268 | FALSE | TRUE | FALSE |
| OGT | O15294 | OID31300 | FALSE | TRUE | FALSE |
| OPHN1 | O60890 | OID31087 | FALSE | TRUE | FALSE |
| PFDN6 | O15212 | OID30938 | FALSE | FALSE | TRUE |
| POF1B | Q8WVV4 | OID30504 | FALSE | TRUE | FALSE |
| RAB33A | Q14088 | OID30122 | FALSE | TRUE | FALSE |
| RAB39B | Q96DA2 | OID30069 | FALSE | TRUE | FALSE |
| RGL2 | O15211 | OID31297 | FALSE | FALSE | TRUE |
| RNF5 | Q99942 | OID30218 | FALSE | FALSE | TRUE |
| RPGR | Q92834 | OID30959 | FALSE | TRUE | FALSE |
| S100G | P29377 | OID31051 | FALSE | TRUE | FALSE |
| SAT1 | P21673 | OID30111 | FALSE | TRUE | FALSE |
| SERPINA7 | P05543 | OID30724 | FALSE | TRUE | FALSE |
| SKIV2L | Q15477 | OID30056 | FALSE | FALSE | TRUE |
| SLC44A4 | Q53GD3 | OID30897 | FALSE | FALSE | TRUE |
| SMS | P52788 | OID30853 | FALSE | TRUE | FALSE |
| SPACA5_SPACA5B | Q96QH8 | OID30990 | TRUE | TRUE | FALSE |
| SRPX | P78539 | OID30310 | FALSE | TRUE | FALSE |
| SYAP1 | Q96A49 | OID30636 | FALSE | TRUE | FALSE |
| SYNGAP1 | Q96PV0 | OID30797 | FALSE | FALSE | TRUE |
| SYTL4 | Q96C24 | OID30339 | FALSE | TRUE | FALSE |
| TAP1 | Q03518 | OID31289 | FALSE | FALSE | TRUE |
| TIMM8A | O60220 | OID31143 | FALSE | TRUE | FALSE |
| TRIM26 | Q12899 | OID31207 | FALSE | FALSE | TRUE |
| TRIM40 | Q6P9F5 | OID30885 | FALSE | FALSE | TRUE |
| TSPAN7 | P41732 | OID30810 | FALSE | TRUE | FALSE |
| WDR46 | O15213 | OID31389 | FALSE | FALSE | TRUE |
| XIAP | P98170 | OID30490 | FALSE | TRUE | FALSE |
| ZNF75D | P51815 | OID31303 | FALSE | TRUE | FALSE |

# **Supplementary Table S2. Instrument variables for proteins associated with lipid fractions at P value ≤ 0.05**

| **Protein** | **UniProtID** | **ProbeID** | **Outcome** | **SNP** | **Effect**  **allele** | **Other**  **allele** | **Beta**  **exposure** | **Se**  **exposure** | **Beta**  **outcome** | **Se**  **outcome** | **F**  **statistics** | **Steiger**  **direction** | **Steiger**  **P value** |
| --- | --- | --- | --- | --- | --- | --- | --- | --- | --- | --- | --- | --- | --- |
| ACP6 | Q9NPH0 | OID21432 | LDL | rs4950465 | T | C | 1.08 | 0.05 | -0.02 | 0.01 | 507.12 | TRUE | 5.9E-181 |
| ADAM8 | P78325 | OID21039 | LDL | rs2995310 | T | C | -0.69 | 0.08 | 0.02 | 0.01 | 74.62 | TRUE | 3.4E-18 |
| ANGPTL3 | Q9Y5C1 | OID20407 | LDL | rs11207980 | C | T | -0.30 | 0.05 | -0.03 | 0.01 | 40.04 | TRUE | 1.0E-08 |
| B4GALT1 | P15291 | OID20780 | LDL | rs7019909 | T | C | 0.38 | 0.06 | 0.02 | 0.01 | 46.99 | TRUE | 1.9E-10 |
| BPIFB1 | Q8TDL5 | OID20308 | LDL | rs2424957 | C | T | -0.35 | 0.04 | -0.02 | 0.01 | 63.57 | TRUE | 1.8E-12 |
| CCS | O14618 | OID20973 | LDL | rs498045 | G | A | 0.39 | 0.04 | 0.01 | 0.01 | 75.49 | TRUE | 3.5E-15 |
| CD4 | P01730 | OID20584 | LDL | rs73053728 | G | A | -0.41 | 0.07 | 0.03 | 0.01 | 33.92 | TRUE | 3.7E-07 |
| CLSTN2 | Q9H4D0 | OID20664 | LDL | rs2197720 | T | C | -0.33 | 0.05 | 0.02 | 0.01 | 43.85 | TRUE | 1.5E-10 |
| CNTN5 | O94779 | OID21006 | LDL | rs7115147 | C | T | 0.32 | 0.05 | -0.02 | 0.01 | 47.29 | TRUE | 3.8E-10 |
| DLK1 | P80370 | OID20295 | LDL | rs12881760 | C | G | 0.52 | 0.05 | -0.02 | 0.01 | 122.69 | TRUE | 3.4E-27 |
| DRAXIN | Q8NBI3 | OID20917 | LDL | rs12139487 | C | T | -0.42 | 0.07 | -0.02 | 0.01 | 35.38 | TRUE | 4.0E-08 |
| GAS6 | Q14393 | OID20318 | LDL | rs6602909 | C | T | -0.36 | 0.05 | 0.02 | 0.01 | 58.26 | TRUE | 9.7E-13 |
| IGF2R | P11717 | OID21146 | LDL | rs3777399 | A | G | 0.56 | 0.06 | -0.03 | 0.01 | 80.94 | TRUE | 1.6E-17 |
| ISLR2 | Q6UXK2 | OID20889 | LDL | rs12440952 | G | A | 0.30 | 0.05 | 0.02 | 0.01 | 41.84 | TRUE | 2.5E-08 |
| LILRB5 | O75023 | OID20324 | LDL | rs12975366 | C | T | -1.15 | 0.05 | -0.02 | 0.01 | 527.66 | TRUE | 8.1E-222 |
| MMP12 | P39900 | OID21439 | LDL | rs2276109 | C | T | -0.82 | 0.08 | 0.03 | 0.01 | 104.73 | TRUE | 8.6E-19 |
| NPTXR | O95502 | OID20191 | LDL | rs2280974 | C | A | -0.35 | 0.05 | 0.01 | 0.01 | 41.51 | TRUE | 4.8E-13 |
| NPTXR | O95502 | OID20191 | LDL | rs56187235 | C | T | 0.86 | 0.09 | -0.05 | 0.01 | 101.10 | TRUE | 3.7E-28 |
| NUCB2 | P80303 | OID21448 | LDL | rs7342262 | G | A | -0.40 | 0.05 | 0.02 | 0.01 | 63.57 | TRUE | 4.1E-15 |
| PCSK9 | Q8NBP7 | OID20235 | LDL | rs2495477 | G | A | -0.32 | 0.05 | -0.05 | 0.01 | 39.93 | TRUE | 1.3E-08 |
| PON2 | Q15165 | OID20088 | LDL | rs6961773 | G | T | -1.05 | 0.06 | -0.02 | 0.01 | 347.74 | TRUE | 2.5E-146 |
| PROK1 | P58294 | OID20543 | LDL | rs1857512 | A | G | 0.60 | 0.05 | 0.02 | 0.01 | 126.83 | TRUE | 2.2E-19 |
| SIGLEC9 | Q9Y336 | OID21390 | LDL | rs2075803 | G | A | -0.55 | 0.04 | 0.02 | 0.01 | 167.09 | TRUE | 2.9E-30 |
| SIRPB1 | O00241 | OID20739 | LDL | rs7271240 | A | C | -0.64 | 0.06 | 0.02 | 0.01 | 97.43 | TRUE | 2.9E-21 |
| TNFSF13 | O75888 | OID20733 | LDL | rs3803800 | G | A | -0.28 | 0.05 | 0.02 | 0.01 | 33.32 | TRUE | 1.5E-07 |
| VSTM1 | Q6UX27 | OID20851 | LDL | rs662850 | T | C | -0.69 | 0.05 | -0.02 | 0.01 | 179.95 | TRUE | 7.2E-46 |
| ADAMTSL5 | Q6ZMM2 | OID30355 | LDL | rs197198 | C | T | 0.26 | 0.05 | 0.02 | 0.01 | 32.57 | TRUE | 6.1E-07 |
| APOE | P02649 | OID30727 | LDL | rs429358 | C | T | -0.82 | 0.07 | 0.10 | 0.01 | 123.55 | TRUE | 3.3E-23 |
| CELSR2 | Q9HCU4 | OID30593 | LDL | rs660240 | C | T | -0.46 | 0.05 | 0.17 | 0.01 | 72.08 | TRUE | 1.4E-08 |
| CLEC4M | Q9H2X3 | OID30405 | LDL | rs4804774 | C | G | -0.36 | 0.05 | 0.02 | 0.01 | 58.27 | TRUE | 6.5E-12 |
| CYB5R2 | Q6BCY4 | OID31512 | LDL | rs3897382 | G | A | 0.33 | 0.05 | -0.02 | 0.01 | 44.15 | TRUE | 4.5E-11 |
| ECM1 | Q16610 | OID30736 | LDL | rs3737240 | T | C | -0.59 | 0.05 | -0.02 | 0.01 | 120.88 | TRUE | 8.9E-33 |
| EPPK1 | P58107 | OID30250 | LDL | rs4073082 | T | C | -0.34 | 0.05 | 0.03 | 0.01 | 43.34 | TRUE | 1.7E-10 |
| F11 | P03951 | OID30773 | LDL | rs2289252 | T | C | 0.41 | 0.05 | -0.02 | 0.01 | 69.89 | TRUE | 2.9E-14 |
| GRIK2 | Q13002 | OID30977 | LDL | rs9498418 | T | A | 0.47 | 0.07 | -0.02 | 0.01 | 45.75 | TRUE | 3.3E-11 |
| HBZ | P02008 | OID30307 | LDL | rs2541638 | C | T | -1.24 | 0.06 | 0.02 | 0.01 | 441.47 | TRUE | 2.1E-219 |
| HGFAC | Q04756 | OID30718 | LDL | rs4690097 | T | C | -0.42 | 0.05 | 0.03 | 0.01 | 79.06 | TRUE | 5.0E-16 |
| HSBP1 | O75506 | OID30232 | LDL | rs707236 | A | T | -0.46 | 0.05 | -0.02 | 0.01 | 73.67 | TRUE | 8.5E-18 |
| IDO1 | P14902 | OID30230 | LDL | rs3824259 | A | C | 0.32 | 0.06 | -0.02 | 0.01 | 34.39 | TRUE | 3.5E-09 |
| IDO1 | P14902 | OID30563 | LDL | rs3824259 | A | C | 0.32 | 0.05 | -0.02 | 0.01 | 33.65 | TRUE | 7.6E-09 |
| IDO1 | P14902 | OID31050 | LDL | rs3824259 | A | C | 0.33 | 0.05 | -0.02 | 0.01 | 38.13 | TRUE | 8.6E-10 |
| IDO1 | P14902 | OID31474 | LDL | rs3824259 | A | C | 0.32 | 0.05 | -0.02 | 0.01 | 36.06 | TRUE | 2.8E-09 |
| INHBB | P09529 | OID30625 | LDL | rs17050272 | A | G | 0.28 | 0.05 | -0.02 | 0.01 | 29.86 | TRUE | 7.3E-07 |
| LILRA3 | Q8N6C8 | OID30376 | LDL | rs1645788 | G | A | -1.22 | 0.06 | 0.02 | 0.01 | 380.52 | TRUE | 4.6E-157 |
| LPA | P08519 | OID30747 | LDL | rs56393506 | T | C | 0.43 | 0.06 | 0.05 | 0.01 | 52.51 | TRUE | 1.8E-10 |
| PGLYRP2 | Q96PD5 | OID30742 | LDL | rs34440547 | T | C | -1.09 | 0.06 | -0.02 | 0.01 | 365.42 | TRUE | 1.2E-99 |
| PRSS53 | Q2L4Q9 | OID31093 | LDL | rs1023623 | C | T | 1.17 | 0.06 | 0.02 | 0.01 | 361.07 | TRUE | 3.5E-232 |
| RNASE4 | P34096 | OID30705 | LDL | rs10145502 | T | A | -0.35 | 0.05 | -0.03 | 0.01 | 43.26 | TRUE | 3.4E-10 |
| SAT2 | Q96F10 | OID31306 | LDL | rs9901675 | A | G | -1.01 | 0.08 | -0.03 | 0.01 | 172.13 | TRUE | 7.6E-46 |
| VNN1 | O95497 | OID30711 | LDL | rs2272996 | C | T | -0.88 | 0.05 | -0.01 | 0.01 | 253.78 | TRUE | 2.3E-77 |
| VNN1 | O95497 | OID30711 | LDL | rs764263 | C | G | 0.50 | 0.05 | 0.02 | 0.01 | 85.17 | TRUE | 5.2E-22 |
| VNN1 | O95497 | OID30711 | LDL | rs8192621 | C | T | 0.61 | 0.10 | 0.00 | 0.02 | 35.40 | TRUE | 6.2E-10 |
| ACVRL1 | P37023 | OID20999 | HDL | rs12230575 | C | T | 0.54 | 0.10 | 0.04 | 0.02 | 30.03 | TRUE | 1.6E-06 |
| ADAM15 | Q13444 | OID20109 | HDL | rs11589479 | A | G | 1.04 | 0.07 | -0.03 | 0.01 | 229.43 | TRUE | 1.8E-55 |
| ANGPTL1 | O95841 | OID20211 | HDL | rs4650993 | G | A | -0.29 | 0.05 | -0.02 | 0.01 | 37.20 | TRUE | 8.1E-09 |
| ANGPTL3 | Q9Y5C1 | OID20407 | HDL | rs11207980 | C | T | -0.30 | 0.05 | -0.03 | 0.01 | 40.04 | TRUE | 6.3E-09 |
| ANGPTL4 | Q9BY76 | OID20703 | HDL | rs2010871 | T | C | 0.30 | 0.05 | 0.03 | 0.01 | 39.57 | TRUE | 1.0E-08 |
| BPIFB1 | Q8TDL5 | OID20308 | HDL | rs2424957 | C | T | -0.35 | 0.04 | -0.01 | 0.01 | 63.57 | TRUE | 1.1E-12 |
| BST1 | Q10588 | OID21051 | HDL | rs7683000 | G | A | -1.10 | 0.06 | -0.03 | 0.01 | 298.82 | TRUE | 1.1E-64 |
| CCL17 | Q92583 | OID20745 | HDL | rs62037101 | G | T | 0.55 | 0.10 | 0.03 | 0.01 | 33.50 | TRUE | 8.7E-09 |
| CD300E | Q496F6 | OID21418 | HDL | rs511074 | T | C | -0.51 | 0.05 | 0.02 | 0.01 | 97.12 | TRUE | 3.0E-22 |
| CD300LF | Q8TDQ1 | OID21469 | HDL | rs35489971 | G | A | 1.22 | 0.06 | -0.02 | 0.01 | 469.05 | TRUE | 3.0E-161 |
| CD302 | Q8IX05 | OID21328 | HDL | rs1978585 | A | G | -0.53 | 0.08 | 0.03 | 0.01 | 41.95 | TRUE | 3.7E-09 |
| CLMP | Q9H6B4 | OID21419 | HDL | rs878296 | C | T | 0.32 | 0.04 | -0.02 | 0.01 | 55.06 | TRUE | 8.0E-10 |
| CRISP2 | P16562 | OID21456 | HDL | rs478328 | G | A | 0.66 | 0.04 | 0.02 | 0.01 | 216.66 | TRUE | 8.9E-46 |
| CTSD | P07339 | OID20358 | HDL | rs72850955 | C | T | -0.51 | 0.07 | 0.02 | 0.01 | 51.59 | TRUE | 5.9E-10 |
| DPEP2 | Q9H4A9 | OID21305 | HDL | rs375557408 | A | G | -1.36 | 0.11 | 0.06 | 0.02 | 146.54 | TRUE | 1.4E-43 |
| DPP7 | Q9UHL4 | OID20142 | HDL | rs10747049 | C | G | 0.55 | 0.06 | 0.02 | 0.01 | 87.43 | TRUE | 2.5E-18 |
| FCER2 | P06734 | OID21133 | HDL | rs11260012 | C | T | -0.41 | 0.05 | -0.01 | 0.01 | 74.86 | TRUE | 3.1E-17 |
| FCER2 | P06734 | OID21133 | HDL | rs12980031 | T | G | -0.37 | 0.06 | -0.02 | 0.01 | 36.36 | TRUE | 1.3E-09 |
| GALNT3 | Q14435 | OID20471 | HDL | rs36058563 | A | G | 0.30 | 0.05 | 0.01 | 0.01 | 35.69 | TRUE | 1.1E-08 |
| GDF15 | Q99988 | OID20251 | HDL | rs888663 | T | G | 0.31 | 0.05 | -0.02 | 0.01 | 33.56 | TRUE | 1.2E-07 |
| GSTA1 | P08263 | OID20166 | HDL | rs6458869 | A | C | 0.34 | 0.05 | 0.02 | 0.01 | 52.53 | TRUE | 2.2E-10 |
| GSTA3 | Q16772 | OID21242 | HDL | rs6458869 | A | C | 0.40 | 0.05 | 0.02 | 0.01 | 58.98 | TRUE | 1.2E-13 |
| IL1RN | P18510 | OID20700 | HDL | rs6734238 | G | A | -0.26 | 0.05 | -0.01 | 0.01 | 31.57 | TRUE | 6.3E-07 |
| INHBC | P55103 | OID21093 | HDL | rs61352607 | T | G | -0.61 | 0.08 | 0.04 | 0.01 | 59.08 | TRUE | 3.6E-10 |
| KLK4 | Q9Y5K2 | OID21391 | HDL | rs1654552 | A | C | -0.47 | 0.05 | 0.02 | 0.01 | 93.52 | TRUE | 3.2E-20 |
| LILRA5 | A6NI73 | OID20209 | HDL | rs414135 | T | A | -0.35 | 0.05 | 0.03 | 0.01 | 50.63 | TRUE | 2.6E-09 |
| LILRB2 | Q8N423 | OID20301 | HDL | rs383369 | C | T | -1.12 | 0.06 | 0.07 | 0.01 | 321.14 | TRUE | 6.1E-85 |
| LILRB5 | O75023 | OID20324 | HDL | rs12975366 | C | T | -1.15 | 0.05 | -0.04 | 0.01 | 527.66 | TRUE | 6.0E-216 |
| MEP1B | Q16820 | OID20168 | HDL | rs620982 | C | T | -1.13 | 0.05 | 0.02 | 0.01 | 508.57 | TRUE | 4.5E-209 |
| MLN | P12872 | OID20541 | HDL | rs2281819 | A | T | -0.44 | 0.05 | 0.02 | 0.01 | 63.75 | TRUE | 1.4E-12 |
| MSR1 | P21757 | OID21063 | HDL | rs73665262 | T | C | -0.38 | 0.07 | 0.03 | 0.01 | 32.44 | TRUE | 8.6E-07 |
| NAAA | Q02083 | OID20931 | HDL | rs17001247 | C | T | -0.52 | 0.05 | -0.02 | 0.01 | 101.16 | TRUE | 3.4E-18 |
| NCAM2 | O15394 | OID21095 | HDL | rs34399466 | T | A | 0.46 | 0.07 | 0.02 | 0.01 | 40.90 | TRUE | 5.5E-10 |
| NID2 | Q14112 | OID21085 | HDL | rs1151582 | T | C | -0.38 | 0.04 | -0.01 | 0.01 | 72.18 | TRUE | 6.7E-15 |
| NRP1 | O14786 | OID20390 | HDL | rs2073321 | T | A | -0.31 | 0.05 | -0.02 | 0.01 | 38.53 | TRUE | 2.2E-09 |
| NT5E | P21589 | OID21498 | HDL | rs637346 | C | G | -0.81 | 0.07 | 0.02 | 0.01 | 145.20 | TRUE | 1.2E-31 |
| PLA2G15 | Q8NCC3 | OID21473 | HDL | rs12600062 | A | C | -0.33 | 0.06 | 0.07 | 0.01 | 31.47 | TRUE | 7.0E-06 |
| PLTP | P55058 | OID20238 | HDL | rs6073958 | C | T | -0.49 | 0.05 | -0.02 | 0.01 | 85.39 | TRUE | 2.9E-18 |
| PPCDC | Q96CD2 | OID21027 | HDL | rs58645417 | G | T | 0.58 | 0.05 | -0.01 | 0.01 | 146.25 | TRUE | 3.1E-35 |
| PSMD9 | O00233 | OID21304 | HDL | rs1169072 | G | A | 0.27 | 0.05 | -0.02 | 0.01 | 34.98 | TRUE | 2.0E-07 |
| RET | P07949 | OID21346 | HDL | rs3121280 | C | T | 0.38 | 0.06 | 0.03 | 0.01 | 37.13 | TRUE | 1.2E-07 |
| RGMA | Q96B86 | OID21065 | HDL | rs1969589 | C | T | 0.41 | 0.06 | -0.02 | 0.01 | 49.52 | TRUE | 2.2E-10 |
| SERPINA11 | Q86U17 | OID20281 | HDL | rs1885137 | G | T | -0.66 | 0.05 | 0.01 | 0.01 | 206.53 | TRUE | 9.3E-47 |
| SMOC2 | Q9H3U7 | OID20730 | HDL | rs4708741 | A | G | -0.41 | 0.06 | 0.03 | 0.01 | 49.85 | TRUE | 3.9E-10 |
| SPARCL1 | Q14515 | OID20361 | HDL | rs17012761 | G | T | 0.69 | 0.05 | -0.01 | 0.01 | 207.66 | TRUE | 1.7E-53 |
| SPARCL1 | Q14515 | OID20361 | HDL | rs56324651 | C | G | -0.59 | 0.09 | 0.02 | 0.01 | 38.50 | TRUE | 1.3E-09 |
| TGFBI | Q15582 | OID20417 | HDL | rs916951 | G | A | -0.64 | 0.05 | 0.02 | 0.01 | 175.36 | TRUE | 2.3E-44 |
| THOP1 | P52888 | OID20194 | HDL | rs4807332 | C | T | 0.31 | 0.05 | 0.02 | 0.01 | 43.38 | TRUE | 3.1E-09 |
| TLR3 | O15455 | OID20612 | HDL | rs3775291 | T | C | -1.23 | 0.06 | 0.04 | 0.01 | 442.51 | TRUE | 2.0E-167 |
| TYRO3 | Q06418 | OID20161 | HDL | rs8025212 | G | A | -0.57 | 0.05 | 0.02 | 0.01 | 130.85 | TRUE | 5.0E-31 |
| UMOD | P07911 | OID20237 | HDL | rs76625281 | T | G | 0.43 | 0.07 | 0.00 | 0.01 | 33.02 | TRUE | 1.4E-06 |
| UMOD | P07911 | OID20237 | HDL | rs77924615 | A | G | -0.78 | 0.05 | 0.02 | 0.01 | 224.19 | TRUE | 1.1E-46 |
| ADAMTSL5 | Q6ZMM2 | OID30355 | HDL | rs197198 | C | T | 0.26 | 0.05 | 0.02 | 0.01 | 32.57 | TRUE | 3.4E-07 |
| AFAP1 | Q8N556 | OID30569 | HDL | rs56270003 | T | C | 1.43 | 0.08 | 0.02 | 0.01 | 365.74 | TRUE | 1.1E-106 |
| APOC1 | P02654 | OID30749 | HDL | rs484195 | G | A | 0.32 | 0.05 | -0.03 | 0.01 | 46.56 | TRUE | 8.0E-10 |
| APOE | P02649 | OID30727 | HDL | rs429358 | C | T | -0.82 | 0.07 | -0.12 | 0.01 | 123.55 | TRUE | 4.6E-22 |
| BTD | P43251 | OID30754 | HDL | rs71627135 | C | T | -1.00 | 0.09 | 0.03 | 0.01 | 129.35 | TRUE | 2.8E-40 |
| CDA | P32320 | OID30345 | HDL | rs2072671 | C | A | -0.51 | 0.05 | -0.02 | 0.01 | 94.45 | TRUE | 1.2E-18 |
| CELSR2 | Q9HCU4 | OID30593 | HDL | rs660240 | C | T | -0.46 | 0.05 | -0.05 | 0.01 | 72.08 | TRUE | 6.2E-15 |
| ENDOU | P21128 | OID31498 | HDL | rs11168216 | C | T | 0.37 | 0.04 | 0.02 | 0.01 | 70.42 | TRUE | 1.8E-11 |
| GIPC3 | Q8TF64 | OID31507 | HDL | rs34722692 | A | G | -0.52 | 0.08 | 0.05 | 0.01 | 39.02 | TRUE | 7.8E-09 |
| GSTM4 | Q03013 | OID30164 | HDL | rs36209093 | T | C | -0.39 | 0.06 | 0.04 | 0.01 | 48.55 | TRUE | 1.5E-13 |
| GSTT2B | P0CG30 | OID31097 | HDL | rs5751777 | T | C | -1.13 | 0.05 | -0.02 | 0.01 | 475.42 | TRUE | 3.4E-164 |
| HBZ | P02008 | OID30307 | HDL | rs2541638 | C | T | -1.24 | 0.06 | 0.02 | 0.01 | 441.47 | TRUE | 3.3E-219 |
| INHBB | P09529 | OID30625 | HDL | rs17050272 | A | G | 0.28 | 0.05 | 0.02 | 0.01 | 29.86 | TRUE | 7.2E-07 |
| ITGA2 | P17301 | OID30570 | HDL | rs246521 | A | C | 0.53 | 0.05 | 0.02 | 0.01 | 110.60 | TRUE | 2.2E-27 |
| ITPA | Q9BY32 | OID30082 | HDL | rs1127354 | A | C | -1.35 | 0.08 | 0.02 | 0.01 | 273.35 | TRUE | 2.0E-88 |
| LECT2 | O14960 | OID30320 | HDL | rs31530 | T | C | -0.84 | 0.05 | -0.02 | 0.01 | 343.10 | TRUE | 3.2E-71 |
| LILRA3 | Q8N6C8 | OID30376 | HDL | rs1645788 | G | A | -1.22 | 0.06 | 0.07 | 0.01 | 380.52 | TRUE | 4.7E-148 |
| MTHFSD | Q2M296 | OID31305 | HDL | rs76712943 | C | A | 0.61 | 0.05 | -0.02 | 0.01 | 143.41 | TRUE | 2.8E-38 |
| PECR | Q9BY49 | OID30228 | HDL | rs10198818 | T | A | -0.52 | 0.06 | 0.03 | 0.01 | 88.51 | TRUE | 1.3E-19 |
| PER3 | P56645 | OID30511 | HDL | rs2153733 | C | T | -0.40 | 0.07 | 0.02 | 0.01 | 36.22 | TRUE | 7.3E-09 |
| PI16 | Q6UXB8 | OID30676 | HDL | rs75657649 | C | G | -0.55 | 0.08 | -0.03 | 0.01 | 43.81 | TRUE | 1.8E-09 |
| PINLYP | A6NC86 | OID30651 | HDL | rs2022062 | C | T | 0.76 | 0.05 | -0.02 | 0.01 | 223.25 | TRUE | 8.6E-58 |
| PTGR1 | Q14914 | OID30297 | HDL | rs77083817 | G | A | 0.66 | 0.09 | -0.03 | 0.01 | 51.37 | TRUE | 7.5E-12 |
| RNASE1 | P07998 | OID30672 | HDL | rs61976945 | G | A | -0.61 | 0.11 | -0.03 | 0.02 | 32.62 | TRUE | 2.1E-08 |
| SCN4B | Q8IWT1 | OID30277 | HDL | rs11604096 | A | C | -0.60 | 0.05 | -0.02 | 0.01 | 135.20 | TRUE | 3.4E-31 |
| SERPINE2 | P07093 | OID30359 | HDL | rs13412535 | A | G | -0.62 | 0.07 | -0.03 | 0.01 | 82.38 | TRUE | 7.1E-19 |
| TF | P02787 | OID30783 | HDL | rs8177295 | A | G | 0.46 | 0.05 | -0.02 | 0.01 | 85.65 | TRUE | 4.5E-21 |
| TGOLN2 | O43493 | OID30691 | HDL | rs1061782 | T | C | -0.65 | 0.05 | 0.02 | 0.01 | 190.65 | TRUE | 3.8E-41 |
| VSIG10L | Q86VR7 | OID30189 | HDL | rs28668475 | T | C | 0.29 | 0.05 | -0.02 | 0.01 | 30.70 | TRUE | 5.9E-07 |
| ACP6 | Q9NPH0 | OID21432 | TC | rs4950465 | T | C | 1.08 | 0.05 | -0.02 | 0.01 | 507.12 | TRUE | 6.6E-181 |
| ADAM8 | P78325 | OID21039 | TC | rs2995310 | T | C | -0.69 | 0.08 | 0.02 | 0.01 | 74.62 | TRUE | 4.3E-18 |
| ANGPTL3 | Q9Y5C1 | OID20407 | TC | rs11207980 | C | T | -0.30 | 0.05 | -0.06 | 0.01 | 40.04 | TRUE | 4.1E-07 |
| ASAH2 | Q9NR71 | OID20996 | TC | rs7077415 | A | G | -1.05 | 0.07 | -0.02 | 0.01 | 240.00 | TRUE | 2.1E-75 |
| BPIFB1 | Q8TDL5 | OID20308 | TC | rs2424957 | C | T | -0.35 | 0.04 | -0.02 | 0.01 | 63.57 | TRUE | 1.2E-12 |
| CCS | O14618 | OID20973 | TC | rs498045 | G | A | 0.39 | 0.04 | 0.02 | 0.01 | 75.49 | TRUE | 4.4E-15 |
| CD200R1 | Q8TD46 | OID20595 | TC | rs72491120 | T | G | 1.75 | 0.09 | -0.03 | 0.01 | 377.51 | TRUE | 1.5E-179 |
| CD276 | Q5ZPR3 | OID20680 | TC | rs10083681 | A | G | -1.33 | 0.07 | -0.02 | 0.01 | 324.93 | TRUE | 6.6E-101 |
| CD300E | Q496F6 | OID21418 | TC | rs511074 | T | C | -0.51 | 0.05 | 0.02 | 0.01 | 97.12 | TRUE | 1.1E-22 |
| CLSTN2 | Q9H4D0 | OID20664 | TC | rs2197720 | T | C | -0.33 | 0.05 | 0.02 | 0.01 | 43.85 | TRUE | 8.6E-11 |
| DLK1 | P80370 | OID20295 | TC | rs12881760 | C | G | 0.52 | 0.05 | -0.02 | 0.01 | 122.69 | TRUE | 4.2E-27 |
| FUT8 | Q9BYC5 | OID20992 | TC | rs2127870 | C | G | -1.06 | 0.07 | 0.02 | 0.01 | 228.78 | TRUE | 2.6E-76 |
| GALNT10 | Q86SR1 | OID21371 | TC | rs34164492 | A | G | 0.35 | 0.05 | -0.02 | 0.01 | 44.54 | TRUE | 5.3E-12 |
| GAS6 | Q14393 | OID20318 | TC | rs6602909 | C | T | -0.36 | 0.05 | 0.03 | 0.01 | 58.26 | TRUE | 3.2E-12 |
| HAVCR1 | Q96D42 | OID21422 | TC | rs10462975 | T | C | 0.44 | 0.04 | 0.01 | 0.01 | 101.57 | TRUE | 1.3E-20 |
| HDGF | P51858 | OID21455 | TC | rs11264534 | A | G | 1.09 | 0.05 | -0.02 | 0.01 | 493.57 | TRUE | 8.8E-125 |
| IGF2R | P11717 | OID21146 | TC | rs3777399 | A | G | 0.56 | 0.06 | -0.02 | 0.01 | 80.94 | TRUE | 1.2E-17 |
| IL12B | P29460 | OID20666 | TC | rs4244437 | A | G | 0.47 | 0.05 | 0.02 | 0.01 | 71.65 | TRUE | 4.5E-14 |
| IL2RA | P01589 | OID20267 | TC | rs7093069 | T | C | -0.55 | 0.06 | -0.02 | 0.01 | 80.34 | TRUE | 9.7E-19 |
| LILRB1 | Q8NHL6 | OID20323 | TC | rs1645788 | G | A | -0.63 | 0.06 | 0.02 | 0.01 | 129.15 | TRUE | 1.8E-29 |
| LILRB1 | Q8NHL6 | OID20323 | TC | rs1749311 | G | A | -0.34 | 0.05 | 0.01 | 0.01 | 48.22 | TRUE | 8.3E-13 |
| LILRB2 | Q8N423 | OID20301 | TC | rs383369 | C | T | -1.12 | 0.06 | 0.02 | 0.01 | 321.14 | TRUE | 2.8E-91 |
| LILRB5 | O75023 | OID20324 | TC | rs12975366 | C | T | -1.15 | 0.05 | -0.03 | 0.01 | 527.66 | TRUE | 6.8E-219 |
| MDGA1 | Q8NFP4 | OID20951 | TC | rs6937280 | G | T | -1.19 | 0.05 | 0.02 | 0.01 | 520.69 | TRUE | 2.7E-199 |
| MDGA1 | Q8NFP4 | OID20951 | TC | rs7745284 | T | C | -0.41 | 0.05 | 0.01 | 0.01 | 57.30 | TRUE | 8.8E-16 |
| MMP12 | P39900 | OID21439 | TC | rs2276109 | C | T | -0.82 | 0.08 | 0.03 | 0.01 | 104.73 | TRUE | 1.1E-18 |
| NUCB2 | P80303 | OID21448 | TC | rs7342262 | G | A | -0.40 | 0.05 | 0.02 | 0.01 | 63.57 | TRUE | 6.1E-15 |
| PCSK9 | Q8NBP7 | OID20235 | TC | rs2495477 | G | A | -0.32 | 0.05 | -0.04 | 0.01 | 39.93 | TRUE | 3.3E-09 |
| PDGFRB | P09619 | OID20268 | TC | rs2304058 | G | C | 1.13 | 0.05 | 0.02 | 0.01 | 536.54 | TRUE | 2.0E-191 |
| PLA2G15 | Q8NCC3 | OID21473 | TC | rs12600062 | A | C | -0.33 | 0.06 | 0.02 | 0.01 | 31.47 | TRUE | 1.2E-07 |
| SIRPB1 | O00241 | OID20739 | TC | rs7271240 | A | C | -0.64 | 0.06 | 0.02 | 0.01 | 97.43 | TRUE | 4.0E-21 |
| SSC5D | A1L4H1 | OID20351 | TC | rs8103017 | G | C | 0.51 | 0.05 | 0.02 | 0.01 | 85.90 | TRUE | 7.4E-19 |
| SUSD2 | Q9UGT4 | OID21098 | TC | rs62233115 | C | T | 0.42 | 0.05 | 0.02 | 0.01 | 63.52 | TRUE | 2.5E-16 |
| TMPRSS5 | Q9H3S3 | OID20947 | TC | rs4938033 | T | G | 0.77 | 0.05 | -0.02 | 0.01 | 225.61 | TRUE | 4.9E-57 |
| TNFSF13 | O75888 | OID20733 | TC | rs3803800 | G | A | -0.28 | 0.05 | 0.03 | 0.01 | 33.32 | TRUE | 2.2E-07 |
| VSTM1 | Q6UX27 | OID20851 | TC | rs662850 | T | C | -0.69 | 0.05 | -0.02 | 0.01 | 179.95 | TRUE | 2.9E-46 |
| WFIKKN1 | Q96NZ8 | OID20939 | TC | rs11548188 | A | G | 0.29 | 0.05 | 0.02 | 0.01 | 34.60 | TRUE | 3.3E-07 |
| ADAMTSL5 | Q6ZMM2 | OID30355 | TC | rs197198 | C | T | 0.26 | 0.05 | 0.03 | 0.01 | 32.57 | TRUE | 8.8E-07 |
| APOC1 | P02654 | OID30749 | TC | rs484195 | G | A | 0.32 | 0.05 | 0.04 | 0.01 | 46.56 | TRUE | 4.9E-09 |
| APOE | P02649 | OID30727 | TC | rs429358 | C | T | -0.82 | 0.07 | 0.13 | 0.01 | 123.55 | TRUE | 1.0E-21 |
| ARHGEF5 | Q12774 | OID30871 | TC | rs1635077 | G | A | -0.25 | 0.04 | 0.02 | 0.01 | 33.78 | TRUE | 9.1E-06 |
| C8B | P07358 | OID30719 | TC | rs620910 | C | T | 0.42 | 0.05 | 0.01 | 0.01 | 71.77 | TRUE | 3.3E-18 |
| CELSR2 | Q9HCU4 | OID30593 | TC | rs660240 | C | T | -0.46 | 0.05 | 0.14 | 0.01 | 72.08 | TRUE | 5.8E-10 |
| CLGN | O14967 | OID30957 | TC | rs6852397 | T | G | 1.17 | 0.07 | 0.02 | 0.01 | 246.20 | TRUE | 7.6E-65 |
| CRYM | Q14894 | OID30815 | TC | rs7194883 | C | T | -0.46 | 0.06 | 0.02 | 0.01 | 55.30 | TRUE | 2.4E-15 |
| EPPK1 | P58107 | OID30250 | TC | rs4073082 | T | C | -0.34 | 0.05 | 0.03 | 0.01 | 43.34 | TRUE | 1.0E-10 |
| F11 | P03951 | OID30773 | TC | rs2289252 | T | C | 0.41 | 0.05 | -0.02 | 0.01 | 69.89 | TRUE | 2.2E-14 |
| FGFR4 | P22455 | OID30646 | TC | rs451643 | T | G | 0.90 | 0.05 | -0.02 | 0.01 | 361.53 | TRUE | 7.3E-95 |
| GASK1A | Q9UFP1 | OID30109 | TC | rs10865927 | A | G | 0.40 | 0.06 | -0.01 | 0.01 | 52.03 | TRUE | 3.4E-16 |
| GASK1A | Q9UFP1 | OID30109 | TC | rs3732858 | A | G | -1.08 | 0.06 | 0.01 | 0.01 | 358.83 | TRUE | 8.3E-166 |
| GRIK2 | Q13002 | OID30977 | TC | rs9498418 | T | A | 0.47 | 0.07 | -0.03 | 0.01 | 45.75 | TRUE | 4.6E-11 |
| GRP | P07492 | OID30264 | TC | rs7243357 | G | T | 0.40 | 0.06 | -0.02 | 0.01 | 50.28 | TRUE | 5.2E-12 |
| HBZ | P02008 | OID30307 | TC | rs2541638 | C | T | -1.24 | 0.06 | 0.02 | 0.01 | 441.47 | TRUE | 2.4E-217 |
| HGFAC | Q04756 | OID30718 | TC | rs4690097 | T | C | -0.42 | 0.05 | 0.03 | 0.01 | 79.06 | TRUE | 7.0E-16 |
| HSBP1 | O75506 | OID30232 | TC | rs707236 | A | T | -0.46 | 0.05 | -0.02 | 0.01 | 73.67 | TRUE | 8.9E-18 |
| INHBB | P09529 | OID30625 | TC | rs17050272 | A | G | 0.28 | 0.05 | -0.02 | 0.01 | 29.86 | TRUE | 9.0E-07 |
| LILRA3 | Q8N6C8 | OID30376 | TC | rs1645788 | G | A | -1.22 | 0.06 | 0.02 | 0.01 | 380.52 | TRUE | 1.3E-156 |
| LPA | P08519 | OID30747 | TC | rs56393506 | T | C | 0.43 | 0.06 | 0.04 | 0.01 | 52.51 | TRUE | 7.0E-11 |
| PCSK7 | Q16549 | OID31410 | TC | rs1242127 | G | A | -0.42 | 0.05 | 0.02 | 0.01 | 71.61 | TRUE | 3.4E-18 |
| RNASE4 | P34096 | OID30705 | TC | rs10145502 | T | A | -0.35 | 0.05 | -0.03 | 0.01 | 43.26 | TRUE | 9.9E-10 |
| SAT2 | Q96F10 | OID31306 | TC | rs9901675 | A | G | -1.01 | 0.08 | -0.03 | 0.01 | 172.13 | TRUE | 1.1E-45 |
| TF | P02787 | OID30783 | TC | rs8177295 | A | G | 0.46 | 0.05 | -0.01 | 0.01 | 85.65 | TRUE | 9.6E-22 |
| ANGPTL3 | Q9Y5C1 | OID20407 | TG | rs11207980 | C | T | -0.30 | 0.05 | -0.08 | 0.01 | 40.04 | TRUE | 2.7E-06 |
| ANGPTL4 | Q9BY76 | OID20703 | TG | rs2010871 | T | C | 0.30 | 0.05 | -0.02 | 0.01 | 39.57 | TRUE | 5.2E-09 |
| AOC3 | Q16853 | OID20389 | TG | rs34908272 | A | G | -0.76 | 0.09 | 0.03 | 0.01 | 75.66 | TRUE | 1.6E-18 |
| APOH | P02749 | OID21072 | TG | rs1801692 | T | C | 0.70 | 0.11 | -0.03 | 0.02 | 42.58 | TRUE | 3.2E-10 |
| ARSB | P15848 | OID21331 | TG | rs60200329 | C | T | -0.38 | 0.06 | 0.02 | 0.01 | 46.36 | TRUE | 5.1E-11 |
| CD4 | P01730 | OID20584 | TG | rs73053728 | G | A | -0.41 | 0.07 | 0.03 | 0.01 | 33.92 | TRUE | 4.1E-07 |
| CEACAM1 | P13688 | OID21528 | TG | rs16975865 | C | T | -0.38 | 0.06 | -0.02 | 0.01 | 36.94 | TRUE | 3.3E-08 |
| CGREF1 | Q99674 | OID20152 | TG | rs6742004 | C | A | -0.43 | 0.05 | 0.03 | 0.01 | 72.45 | TRUE | 1.2E-15 |
| CLPS | P04118 | OID21156 | TG | rs3834738 | C | T | 0.62 | 0.06 | 0.02 | 0.01 | 106.23 | TRUE | 1.2E-35 |
| CST3 | P01034 | OID20400 | TG | rs2104005 | A | G | -0.30 | 0.05 | -0.02 | 0.01 | 41.45 | TRUE | 4.6E-08 |
| DDX58 | O95786 | OID21226 | TG | rs10970985 | G | C | 0.35 | 0.05 | 0.02 | 0.01 | 46.84 | TRUE | 1.8E-11 |
| DPEP2 | Q9H4A9 | OID21305 | TG | rs375557408 | A | G | -1.36 | 0.11 | -0.04 | 0.02 | 146.54 | TRUE | 5.4E-45 |
| ENTPD6 | O75354 | OID20100 | TG | rs56129418 | A | G | -0.44 | 0.08 | 0.03 | 0.01 | 31.80 | TRUE | 1.2E-06 |
| FCER2 | P06734 | OID21133 | TG | rs11260012 | C | T | -0.41 | 0.05 | 0.02 | 0.01 | 74.86 | TRUE | 5.8E-17 |
| FCER2 | P06734 | OID21133 | TG | rs12980031 | T | G | -0.37 | 0.06 | 0.01 | 0.01 | 36.36 | TRUE | 5.1E-10 |
| GALNT10 | Q86SR1 | OID21371 | TG | rs34164492 | A | G | 0.35 | 0.05 | -0.02 | 0.01 | 44.54 | TRUE | 8.4E-12 |
| GAS6 | Q14393 | OID20318 | TG | rs6602909 | C | T | -0.36 | 0.05 | 0.03 | 0.01 | 58.26 | TRUE | 2.2E-12 |
| GSTA1 | P08263 | OID20166 | TG | rs6458869 | A | C | 0.34 | 0.05 | -0.03 | 0.01 | 52.53 | TRUE | 5.1E-10 |
| GSTA3 | Q16772 | OID21242 | TG | rs6458869 | A | C | 0.40 | 0.05 | -0.03 | 0.01 | 58.98 | TRUE | 3.3E-13 |
| HDGF | P51858 | OID21455 | TG | rs11264534 | A | G | 1.09 | 0.05 | -0.02 | 0.01 | 493.57 | TRUE | 7.6E-125 |
| IGFBPL1 | Q8WX77 | OID20155 | TG | rs12235350 | T | C | 0.38 | 0.06 | 0.02 | 0.01 | 45.89 | TRUE | 9.3E-11 |
| IGFBPL1 | Q8WX77 | OID20155 | TG | rs1923424 | A | C | 0.39 | 0.05 | 0.01 | 0.01 | 51.96 | TRUE | 5.4E-14 |
| IGFBPL1 | Q8WX77 | OID20155 | TG | rs28759073 | A | G | -0.36 | 0.06 | -0.01 | 0.01 | 42.75 | TRUE | 1.3E-12 |
| IL17D | Q8TAD2 | OID20481 | TG | rs9506551 | C | T | -0.44 | 0.05 | -0.02 | 0.01 | 65.03 | TRUE | 4.0E-14 |
| IL1R2 | P27930 | OID20757 | TG | rs4851521 | T | C | -0.41 | 0.05 | 0.01 | 0.01 | 63.40 | TRUE | 2.2E-17 |
| LILRA5 | A6NI73 | OID20209 | TG | rs414135 | T | A | -0.35 | 0.05 | -0.02 | 0.01 | 50.63 | TRUE | 3.6E-10 |
| LYPD3 | O95274 | OID21530 | TG | rs2682567 | C | T | 0.42 | 0.06 | -0.02 | 0.01 | 48.61 | TRUE | 1.2E-11 |
| MEGF9 | Q9H1U4 | OID20285 | TG | rs1324472 | T | C | 0.42 | 0.05 | 0.02 | 0.01 | 61.59 | TRUE | 3.5E-14 |
| MFGE8 | Q08431 | OID21134 | TG | rs7171119 | G | T | 0.36 | 0.05 | -0.02 | 0.01 | 52.00 | TRUE | 4.7E-12 |
| MIA | Q16674 | OID21531 | TG | rs2279699 | A | G | 1.11 | 0.09 | 0.03 | 0.02 | 141.96 | TRUE | 8.7E-25 |
| MLN | P12872 | OID20541 | TG | rs2281819 | A | T | -0.44 | 0.05 | -0.02 | 0.01 | 63.75 | TRUE | 1.4E-12 |
| NCAM1 | P13591 | OID20354 | TG | rs2885208 | C | T | -0.45 | 0.07 | 0.03 | 0.01 | 43.08 | TRUE | 2.9E-10 |
| NFASC | O94856 | OID20634 | TG | rs6668366 | C | G | 0.52 | 0.08 | 0.03 | 0.01 | 40.27 | TRUE | 1.1E-08 |
| NRCAM | Q92823 | OID20229 | TG | rs17334792 | G | A | 0.43 | 0.05 | 0.01 | 0.01 | 72.42 | TRUE | 8.3E-18 |
| NUCB2 | P80303 | OID21448 | TG | rs7342262 | G | A | -0.40 | 0.05 | 0.02 | 0.01 | 63.57 | TRUE | 2.7E-15 |
| PLA2G15 | Q8NCC3 | OID21473 | TG | rs12600062 | A | C | -0.33 | 0.06 | -0.02 | 0.01 | 31.47 | TRUE | 1.4E-07 |
| PLTP | P55058 | OID20238 | TG | rs6073958 | C | T | -0.49 | 0.05 | 0.05 | 0.01 | 85.39 | TRUE | 3.5E-16 |
| PRELP | P51888 | OID20766 | TG | rs1417925 | G | T | -0.40 | 0.06 | 0.01 | 0.01 | 37.69 | TRUE | 9.0E-11 |
| PRELP | P51888 | OID20766 | TG | rs28676555 | T | A | -0.58 | 0.07 | 0.02 | 0.01 | 66.11 | TRUE | 8.0E-18 |
| SIRPB1 | O00241 | OID20739 | TG | rs7271240 | A | C | -0.64 | 0.06 | 0.03 | 0.01 | 97.43 | TRUE | 8.4E-21 |
| SMOC2 | Q9H3U7 | OID20730 | TG | rs4708741 | A | G | -0.41 | 0.06 | -0.02 | 0.01 | 49.85 | TRUE | 2.0E-10 |
| SPARCL1 | Q14515 | OID20361 | TG | rs17012761 | G | T | 0.69 | 0.05 | 0.01 | 0.01 | 207.66 | TRUE | 2.6E-53 |
| SPARCL1 | Q14515 | OID20361 | TG | rs56324651 | C | G | -0.59 | 0.09 | -0.01 | 0.01 | 38.50 | TRUE | 5.9E-10 |
| TEK | Q02763 | OID21496 | TG | rs116927451 | T | G | 0.97 | 0.11 | 0.03 | 0.01 | 79.97 | TRUE | 8.6E-22 |
| TFPI | P10646 | OID20388 | TG | rs4140885 | A | G | 0.37 | 0.05 | -0.02 | 0.01 | 51.30 | TRUE | 8.1E-12 |
| TIMP4 | Q99727 | OID21147 | TG | rs2600235 | G | T | 0.74 | 0.08 | 0.03 | 0.01 | 75.90 | TRUE | 3.1E-17 |
| VEGFA | P15692 | OID20650 | TG | rs6921438 | A | G | -0.57 | 0.05 | -0.02 | 0.01 | 140.13 | TRUE | 1.2E-32 |
| VWC2 | Q2TAL6 | OID20936 | TG | rs2114098 | G | A | -0.61 | 0.05 | -0.02 | 0.01 | 134.90 | TRUE | 6.2E-31 |
| AGT | P01019 | OID30709 | TG | rs35837081 | A | G | 0.56 | 0.07 | -0.03 | 0.01 | 58.18 | TRUE | 1.1E-14 |
| APOC1 | P02654 | OID30749 | TG | rs484195 | G | A | 0.32 | 0.05 | 0.09 | 0.01 | 46.56 | TRUE | 1.7E-06 |
| APOE | P02649 | OID30727 | TG | rs429358 | C | T | -0.82 | 0.07 | 0.15 | 0.01 | 123.55 | TRUE | 1.9E-20 |
| CELSR2 | Q9HCU4 | OID30593 | TG | rs660240 | C | T | -0.46 | 0.05 | 0.02 | 0.01 | 72.08 | TRUE | 1.0E-16 |
| CFHR2 | P36980 | OID30720 | TG | rs113141965 | C | T | 0.36 | 0.06 | 0.02 | 0.01 | 33.28 | TRUE | 7.5E-09 |
| CFHR2 | P36980 | OID30720 | TG | rs4085749 | T | C | -0.99 | 0.06 | -0.02 | 0.01 | 317.57 | TRUE | 1.9E-109 |
| CFHR4 | Q92496 | OID30758 | TG | rs1830958 | C | T | -0.75 | 0.05 | -0.02 | 0.01 | 233.01 | TRUE | 1.8E-53 |
| CFHR5 | Q9BXR6 | OID30716 | TG | rs61822230 | A | G | -0.37 | 0.06 | -0.03 | 0.01 | 35.40 | TRUE | 2.7E-11 |
| F13B | P05160 | OID30781 | TG | rs10922161 | G | A | -0.51 | 0.05 | -0.02 | 0.01 | 91.38 | TRUE | 3.5E-22 |
| FGFR4 | P22455 | OID30646 | TG | rs451643 | T | G | 0.90 | 0.05 | -0.02 | 0.01 | 361.53 | TRUE | 1.0E-94 |
| FN1 | P02751 | OID30787 | TG | rs1250247 | G | C | 0.46 | 0.05 | 0.01 | 0.01 | 91.25 | TRUE | 1.2E-21 |
| INHBB | P09529 | OID30625 | TG | rs17050272 | A | G | 0.28 | 0.05 | -0.02 | 0.01 | 29.86 | TRUE | 6.5E-07 |
| KHK | P50053 | OID30241 | TG | rs2304681 | A | G | -0.48 | 0.05 | -0.03 | 0.01 | 85.43 | TRUE | 1.2E-19 |
| KIAA0319 | Q5VV43 | OID31094 | TG | rs9358779 | A | G | 0.32 | 0.05 | -0.02 | 0.01 | 40.74 | TRUE | 6.4E-10 |
| NCR3LG1 | Q68D85 | OID31490 | TG | rs11024271 | C | T | 0.25 | 0.05 | -0.02 | 0.01 | 30.93 | TRUE | 1.8E-06 |
| PCSK7 | Q16549 | OID31410 | TG | rs1242127 | G | A | -0.42 | 0.05 | 0.06 | 0.01 | 71.61 | TRUE | 3.7E-15 |
| PENK | P01210 | OID30666 | TG | rs2609983 | A | G | -0.32 | 0.05 | 0.02 | 0.01 | 44.59 | TRUE | 4.2E-10 |
| REG3G | Q6UW15 | OID31408 | TG | rs434372 | A | T | 0.44 | 0.06 | 0.02 | 0.01 | 64.11 | TRUE | 7.2E-17 |
| RNASE10 | Q5GAN6 | OID30238 | TG | rs7144945 | T | C | -0.79 | 0.05 | 0.02 | 0.01 | 242.54 | TRUE | 1.1E-62 |
| SERPINE2 | P07093 | OID30359 | TG | rs13412535 | A | G | -0.62 | 0.07 | 0.04 | 0.01 | 82.38 | TRUE | 1.2E-18 |
| STAB2 | Q8WWQ8 | OID30401 | TG | rs11111707 | G | C | -0.37 | 0.06 | 0.02 | 0.01 | 38.37 | TRUE | 1.6E-08 |
| TPK1 | Q9H3S4 | OID30338 | TG | rs11763621 | G | T | -0.43 | 0.05 | -0.02 | 0.01 | 65.74 | TRUE | 6.1E-15 |
| ACP6 | Q9NPH0 | OID21432 | nonHDL | rs4950465 | T | C | 1.08 | 0.05 | -0.02 | 0.01 | 507.12 | TRUE | 9.9E-181 |
| ANGPTL3 | Q9Y5C1 | OID20407 | nonHDL | rs11207980 | C | T | -0.30 | 0.05 | -0.05 | 0.01 | 40.04 | TRUE | 1.7E-07 |
| CCS | O14618 | OID20973 | nonHDL | rs498045 | G | A | 0.39 | 0.04 | 0.02 | 0.01 | 75.49 | TRUE | 3.9E-15 |
| CD200R1 | Q8TD46 | OID20595 | nonHDL | rs72491120 | T | G | 1.75 | 0.09 | -0.03 | 0.01 | 377.51 | TRUE | 1.4E-178 |
| CD276 | Q5ZPR3 | OID20680 | nonHDL | rs10083681 | A | G | -1.33 | 0.07 | -0.02 | 0.01 | 324.93 | TRUE | 9.4E-101 |
| CD4 | P01730 | OID20584 | nonHDL | rs73053728 | G | A | -0.41 | 0.07 | 0.03 | 0.01 | 33.92 | TRUE | 3.6E-07 |
| CLSTN2 | Q9H4D0 | OID20664 | nonHDL | rs2197720 | T | C | -0.33 | 0.05 | 0.02 | 0.01 | 43.85 | TRUE | 1.0E-10 |
| DLK1 | P80370 | OID20295 | nonHDL | rs12881760 | C | G | 0.52 | 0.05 | -0.02 | 0.01 | 122.69 | TRUE | 1.8E-27 |
| ENTPD6 | O75354 | OID20100 | nonHDL | rs56129418 | A | G | -0.44 | 0.08 | 0.03 | 0.01 | 31.80 | TRUE | 1.3E-06 |
| FCER2 | P06734 | OID21133 | nonHDL | rs11260012 | C | T | -0.41 | 0.05 | 0.01 | 0.01 | 74.86 | TRUE | 3.1E-17 |
| FCER2 | P06734 | OID21133 | nonHDL | rs12980031 | T | G | -0.37 | 0.06 | 0.01 | 0.01 | 36.36 | TRUE | 6.3E-10 |
| GALNT10 | Q86SR1 | OID21371 | nonHDL | rs34164492 | A | G | 0.35 | 0.05 | -0.02 | 0.01 | 44.54 | TRUE | 6.7E-12 |
| GAS6 | Q14393 | OID20318 | nonHDL | rs6602909 | C | T | -0.36 | 0.05 | 0.03 | 0.01 | 58.26 | TRUE | 5.6E-12 |
| GSTA1 | P08263 | OID20166 | nonHDL | rs6458869 | A | C | 0.34 | 0.05 | -0.02 | 0.01 | 52.53 | TRUE | 1.1E-10 |
| GSTA3 | Q16772 | OID21242 | nonHDL | rs6458869 | A | C | 0.40 | 0.05 | -0.02 | 0.01 | 58.98 | TRUE | 5.7E-14 |
| HAVCR1 | Q96D42 | OID21422 | nonHDL | rs10462975 | T | C | 0.44 | 0.04 | 0.02 | 0.01 | 101.57 | TRUE | 3.3E-20 |
| IFNGR2 | P38484 | OID20941 | nonHDL | rs9808753 | G | A | 1.27 | 0.05 | -0.02 | 0.01 | 537.57 | TRUE | 3.4E-256 |
| IGF2R | P11717 | OID21146 | nonHDL | rs3777399 | A | G | 0.56 | 0.06 | -0.03 | 0.01 | 80.94 | TRUE | 1.9E-17 |
| KIR3DL1 | P43629 | OID21185 | nonHDL | rs11672983 | A | G | -0.94 | 0.05 | 0.01 | 0.01 | 330.70 | TRUE | 3.3E-113 |
| KIR3DL1 | P43629 | OID21185 | nonHDL | rs78732506 | T | C | -0.61 | 0.06 | 0.03 | 0.02 | 113.77 | TRUE | 7.1E-34 |
| LILRB5 | O75023 | OID20324 | nonHDL | rs12975366 | C | T | -1.15 | 0.05 | -0.02 | 0.01 | 527.66 | TRUE | 6.4E-220 |
| NCAM1 | P13591 | OID20354 | nonHDL | rs2885208 | C | T | -0.45 | 0.07 | 0.02 | 0.01 | 43.08 | TRUE | 2.2E-10 |
| NUCB2 | P80303 | OID21448 | nonHDL | rs7342262 | G | A | -0.40 | 0.05 | 0.03 | 0.01 | 63.57 | TRUE | 1.1E-14 |
| PCSK9 | Q8NBP7 | OID20235 | nonHDL | rs2495477 | G | A | -0.32 | 0.05 | -0.04 | 0.01 | 39.93 | TRUE | 5.3E-09 |
| PDGFRB | P09619 | OID20268 | nonHDL | rs2304058 | G | C | 1.13 | 0.05 | 0.01 | 0.01 | 536.54 | TRUE | 5.0E-192 |
| PGLYRP1 | O75594 | OID20338 | nonHDL | rs35247686 | T | C | -0.27 | 0.05 | -0.01 | 0.01 | 29.99 | TRUE | 4.6E-08 |
| PLTP | P55058 | OID20238 | nonHDL | rs6073958 | C | T | -0.49 | 0.05 | 0.02 | 0.01 | 85.39 | TRUE | 5.9E-18 |
| PROK1 | P58294 | OID20543 | nonHDL | rs1857512 | A | G | 0.60 | 0.05 | 0.02 | 0.01 | 126.83 | TRUE | 3.3E-19 |
| S100P | P25815 | OID20103 | nonHDL | rs28667650 | G | C | -0.45 | 0.07 | -0.02 | 0.01 | 41.94 | TRUE | 9.3E-11 |
| SCARA5 | Q6ZMJ2 | OID21058 | nonHDL | rs4496925 | A | G | 0.28 | 0.05 | -0.02 | 0.01 | 30.23 | TRUE | 4.2E-07 |
| SIRPB1 | O00241 | OID20739 | nonHDL | rs7271240 | A | C | -0.64 | 0.06 | 0.03 | 0.01 | 97.43 | TRUE | 5.1E-21 |
| TEK | Q02763 | OID21496 | nonHDL | rs116927451 | T | G | 0.97 | 0.11 | 0.03 | 0.01 | 79.97 | TRUE | 1.1E-21 |
| TGFBI | Q15582 | OID20417 | nonHDL | rs916951 | G | A | -0.64 | 0.05 | -0.02 | 0.01 | 175.36 | TRUE | 6.5E-45 |
| TMPRSS5 | Q9H3S3 | OID20947 | nonHDL | rs4938033 | T | G | 0.77 | 0.05 | -0.02 | 0.01 | 225.61 | TRUE | 9.9E-57 |
| TNFSF13 | O75888 | OID20733 | nonHDL | rs3803800 | G | A | -0.28 | 0.05 | 0.03 | 0.01 | 33.32 | TRUE | 2.8E-07 |
| VEGFA | P15692 | OID20650 | nonHDL | rs6921438 | A | G | -0.57 | 0.05 | -0.02 | 0.01 | 140.13 | TRUE | 6.5E-33 |
| VSTM1 | Q6UX27 | OID20851 | nonHDL | rs662850 | T | C | -0.69 | 0.05 | -0.02 | 0.01 | 179.95 | TRUE | 8.1E-46 |
| WFIKKN1 | Q96NZ8 | OID20939 | nonHDL | rs11548188 | A | G | 0.29 | 0.05 | 0.02 | 0.01 | 34.60 | TRUE | 3.1E-07 |
| ADAMTSL5 | Q6ZMM2 | OID30355 | nonHDL | rs197198 | C | T | 0.26 | 0.05 | 0.02 | 0.01 | 32.57 | TRUE | 5.9E-07 |
| APOC1 | P02654 | OID30749 | nonHDL | rs484195 | G | A | 0.32 | 0.05 | 0.04 | 0.01 | 46.56 | TRUE | 7.3E-09 |
| APOE | P02649 | OID30727 | nonHDL | rs429358 | C | T | -0.82 | 0.07 | 0.15 | 0.01 | 123.55 | TRUE | 1.6E-20 |
| C8B | P07358 | OID30719 | nonHDL | rs620910 | C | T | 0.42 | 0.05 | 0.02 | 0.01 | 71.77 | TRUE | 4.4E-18 |
| CELSR2 | Q9HCU4 | OID30593 | nonHDL | rs660240 | C | T | -0.46 | 0.05 | 0.16 | 0.01 | 72.08 | TRUE | 4.7E-09 |
| CLGN | O14967 | OID30957 | nonHDL | rs6852397 | T | G | 1.17 | 0.07 | 0.03 | 0.01 | 246.20 | TRUE | 2.5E-64 |
| CRYM | Q14894 | OID30815 | nonHDL | rs7194883 | C | T | -0.46 | 0.06 | 0.02 | 0.01 | 55.30 | TRUE | 2.1E-15 |
| EPPK1 | P58107 | OID30250 | nonHDL | rs4073082 | T | C | -0.34 | 0.05 | 0.03 | 0.01 | 43.34 | TRUE | 1.6E-10 |
| FGFR4 | P22455 | OID30646 | nonHDL | rs451643 | T | G | 0.90 | 0.05 | -0.02 | 0.01 | 361.53 | TRUE | 9.5E-95 |
| FN1 | P02751 | OID30787 | nonHDL | rs1250247 | G | C | 0.46 | 0.05 | 0.02 | 0.01 | 91.25 | TRUE | 3.0E-21 |
| GASK1A | Q9UFP1 | OID30109 | nonHDL | rs10865927 | A | G | 0.40 | 0.06 | -0.01 | 0.01 | 52.03 | TRUE | 2.7E-16 |
| GASK1A | Q9UFP1 | OID30109 | nonHDL | rs3732858 | A | G | -1.08 | 0.06 | 0.01 | 0.01 | 358.83 | TRUE | 1.1E-165 |
| GRIK2 | Q13002 | OID30977 | nonHDL | rs9498418 | T | A | 0.47 | 0.07 | -0.03 | 0.01 | 45.75 | TRUE | 7.4E-11 |
| HBZ | P02008 | OID30307 | nonHDL | rs2541638 | C | T | -1.24 | 0.06 | 0.02 | 0.01 | 441.47 | TRUE | 1.7E-218 |
| HGFAC | Q04756 | OID30718 | nonHDL | rs4690097 | T | C | -0.42 | 0.05 | 0.03 | 0.01 | 79.06 | TRUE | 7.8E-16 |
| HSBP1 | O75506 | OID30232 | nonHDL | rs707236 | A | T | -0.46 | 0.05 | -0.02 | 0.01 | 73.67 | TRUE | 2.1E-17 |
| INHBB | P09529 | OID30625 | nonHDL | rs17050272 | A | G | 0.28 | 0.05 | -0.03 | 0.01 | 29.86 | TRUE | 1.3E-06 |
| LPA | P08519 | OID30747 | nonHDL | rs56393506 | T | C | 0.43 | 0.06 | 0.03 | 0.01 | 52.51 | TRUE | 5.5E-11 |
| NCR3LG1 | Q68D85 | OID31490 | nonHDL | rs11024271 | C | T | 0.25 | 0.05 | -0.02 | 0.01 | 30.93 | TRUE | 1.3E-06 |
| PCSK7 | Q16549 | OID31410 | nonHDL | rs1242127 | G | A | -0.42 | 0.05 | 0.02 | 0.01 | 71.61 | TRUE | 2.1E-18 |
| PGLYRP2 | Q96PD5 | OID30742 | nonHDL | rs34440547 | T | C | -1.09 | 0.06 | -0.02 | 0.01 | 365.42 | TRUE | 1.5E-99 |
| RNASE4 | P34096 | OID30705 | nonHDL | rs10145502 | T | A | -0.35 | 0.05 | -0.03 | 0.01 | 43.26 | TRUE | 5.9E-10 |
| SAT2 | Q96F10 | OID31306 | nonHDL | rs9901675 | A | G | -1.01 | 0.08 | -0.03 | 0.01 | 172.13 | TRUE | 1.2E-45 |
| SERPINE2 | P07093 | OID30359 | nonHDL | rs13412535 | A | G | -0.62 | 0.07 | 0.03 | 0.01 | 82.38 | TRUE | 3.5E-19 |

# **Supplementary Table S3. Proteome-wide MR results for associations with P ≤ 0.05**

| **Protein** | **UniProtID** | **.ProbeID** | **Outcome** | **Method** | **N (SNP)** | **Beta (95% Confidence Interval)** | **P** | **P (FDR)** | | **P (Bonferroni)** | |
| --- | --- | --- | --- | --- | --- | --- | --- | --- | --- | --- | --- |
| ACP6 | Q9NPH0 | OID21432 | TC | Wald ratio | 1 | -0.02 (-0.03, 0) | 9.2E-03 | 0.28 | | 1 | |
| ACP6 | Q9NPH0 | OID21432 | LDL | Wald ratio | 1 | -0.02 (-0.03, 0) | 0.01 | 0.38 | | 1 | |
| ACP6 | Q9NPH0 | OID21432 | nonHDL | Wald ratio | 1 | -0.02 (-0.03, 0) | 0.01 | 0.38 | | 1 | |
| ACVRL1 | P37023 | OID20999 | HDL | Wald ratio | 1 | 0.07 (0.01, 0.12) | 0.02 | 0.26 | | 1 | |
| ADAM15 | Q13444 | OID20109 | HDL | Wald ratio | 1 | -0.03 (-0.05, -0.01) | 0.01 | 0.21 | | 1 | |
| ADAM8 | P78325 | OID21039 | TC | Wald ratio | 1 | -0.04 (-0.07, 0) | 0.03 | 0.51 | | 1 | |
| ADAM8 | P78325 | OID21039 | LDL | Wald ratio | 1 | -0.03 (-0.06, 0) | 0.05 | 0.71 | | 1 | |
| ADAMTSL5 | Q6ZMM2 | OID30355 | TC | Wald ratio | 1 | 0.1 (0.04, 0.15) | 4.9E-04 | 0.03 | | 0.35 | |
| ADAMTSL5 | Q6ZMM2 | OID30355 | LDL | Wald ratio | 1 | 0.09 (0.03, 0.14) | 2.1E-03 | 0.15 | | 1 | |
| ADAMTSL5 | Q6ZMM2 | OID30355 | nonHDL | Wald ratio | 1 | 0.08 (0.03, 0.14) | 3.5E-03 | 0.17 | | 1 | |
| ADAMTSL5 | Q6ZMM2 | OID30355 | HDL | Wald ratio | 1 | 0.07 (0.01, 0.12) | 0.02 | 0.28 | | 1 | |
| AFAP1 | Q8N556 | OID30569 | HDL | Wald ratio | 1 | 0.02 (0, 0.03) | 0.02 | 0.31 | | 1 | |
| AGT | P01019 | OID30709 | TG | Wald ratio | 1 | -0.06 (-0.09, -0.02) | 2.8E-03 | 0.16 | | 1 | |
| ANGPTL1 | O95841 | OID20211 | HDL | Wald ratio | 1 | 0.06 (0.01, 0.1) | 0.02 | 0.28 | | 1 | |
| ANGPTL3 | Q9Y5C1 | OID20407 | TG | Wald ratio | 1 | 0.26 (0.22, 0.31) | 2.4E-29 | 5.7E-27 | | 1.7E-26 | |
| ANGPTL3 | Q9Y5C1 | OID20407 | TC | Wald ratio | 1 | 0.21 (0.16, 0.25) | 1.6E-18 | 3.8E-16 | | 1.1E-15 | |
| ANGPTL3 | Q9Y5C1 | OID20407 | nonHDL | Wald ratio | 1 | 0.18 (0.13, 0.23) | 1.6E-13 | 3.7E-11 | | 1.1E-10 | |
| ANGPTL3 | Q9Y5C1 | OID20407 | LDL | Wald ratio | 1 | 0.1 (0.06, 0.15) | 1.5E-05 | 2.1E-03 | | 0.01 | |
| ANGPTL3 | Q9Y5C1 | OID20407 | HDL | Wald ratio | 1 | 0.09 (0.04, 0.13) | 2.1E-04 | 0.01 | | 0.15 | |
| ANGPTL4 | Q9BY76 | OID20703 | HDL | Wald ratio | 1 | 0.09 (0.04, 0.13) | 3.9E-04 | 0.02 | | 0.27 | |
| ANGPTL4 | Q9BY76 | OID20703 | TG | Wald ratio | 1 | -0.07 (-0.12, -0.02) | 4.2E-03 | 0.17 | | 1 | |
| AOC3 | Q16853 | OID20389 | TG | Wald ratio | 1 | -0.04 (-0.08, 0) | 0.04 | 0.53 | | 1 | |
| APOC1 | P02654 | OID30749 | TG | Wald ratio | 1 | 0.29 (0.24, 0.34) | 1.4E-32 | 9.6E-30 | | 9.6E-30 | |
| APOC1 | P02654 | OID30749 | nonHDL | Wald ratio | 1 | 0.14 (0.09, 0.19) | 4.1E-08 | 7.2E-06 | | 2.9E-05 | |
| APOC1 | P02654 | OID30749 | TC | Wald ratio | 1 | 0.13 (0.08, 0.18) | 1.6E-07 | 2.9E-05 | | 1.2E-04 | |
| APOC1 | P02654 | OID30749 | HDL | Wald ratio | 1 | -0.09 (-0.13, -0.04) | 5.5E-04 | 0.03 | | 0.39 | |
| APOE | P02649 | OID30727 | TG | Wald ratio | 1 | -0.19 (-0.22, -0.16) | 1.2E-31 | 4.3E-29 | | 8.6E-29 | |
| APOE | P02649 | OID30727 | nonHDL | Wald ratio | 1 | -0.19 (-0.22, -0.15) | 7.3E-29 | 2.6E-26 | | 5.2E-26 | |
| APOE | P02649 | OID30727 | TC | Wald ratio | 1 | -0.15 (-0.18, -0.12) | 1.3E-21 | 4.4E-19 | | 8.9E-19 | |
| APOE | P02649 | OID30727 | HDL | Wald ratio | 1 | 0.15 (0.11, 0.18) | 9.3E-20 | 6.5E-17 | | 6.5E-17 | |
| APOE | P02649 | OID30727 | LDL | Wald ratio | 1 | -0.12 (-0.15, -0.09) | 1.0E-13 | 3.6E-11 | | 7.2E-11 | |
| APOH | P02749 | OID21072 | TG | Wald ratio | 1 | -0.04 (-0.09, 0) | 0.05 | 0.54 | | 1 | |
| ARHGEF5 | Q12774 | OID30871 | TC | Wald ratio | 1 | -0.07 (-0.13, -0.01) | 0.02 | 0.51 | | 1 | |
| ARSB | P15848 | OID21331 | TG | Wald ratio | 1 | -0.05 (-0.09, 0) | 0.03 | 0.48 | | 1 | |
| ASAH2 | Q9NR71 | OID20996 | TC | Wald ratio | 1 | 0.02 (0, 0.04) | 0.03 | 0.51 | | 1 | |
| B4GALT1 | P15291 | OID20780 | LDL | Wald ratio | 1 | 0.06 (0.01, 0.1) | 0.01 | 0.37 | | 1 | |
| BPIFB1 | Q8TDL5 | OID20308 | LDL | Wald ratio | 1 | 0.05 (0.01, 0.09) | 0.01 | 0.41 | | 1 | |
| BPIFB1 | Q8TDL5 | OID20308 | TC | Wald ratio | 1 | 0.04 (0, 0.09) | 0.04 | 0.51 | | 1 | |
| BPIFB1 | Q8TDL5 | OID20308 | HDL | Wald ratio | 1 | 0.04 (0, 0.08) | 0.04 | 0.42 | | 1 | |
| BST1 | Q10588 | OID21051 | HDL | Wald ratio | 1 | 0.02 (0.01, 0.04) | 0.01 | 0.21 | | 1 | |
| BTD | P43251 | OID30754 | HDL | Wald ratio | 1 | -0.03 (-0.06, -0.01) | 0.01 | 0.23 | | 1 | |
| C8B | P07358 | OID30719 | nonHDL | Wald ratio | 1 | 0.04 (0, 0.07) | 0.03 | 0.50 | | 1 | |
| C8B | P07358 | OID30719 | TC | Wald ratio | 1 | 0.04 (0, 0.07) | 0.04 | 0.51 | | 1 | |
| CCL17 | Q92583 | OID20745 | HDL | Wald ratio | 1 | 0.06 (0.01, 0.11) | 0.02 | 0.29 | | 1 | |
| CCS | O14618 | OID20973 | TC | Wald ratio | 1 | 0.04 (0.01, 0.08) | 0.03 | 0.51 | | 1 | |
| CCS | O14618 | OID20973 | LDL | Wald ratio | 1 | 0.04 (0, 0.07) | 0.04 | 0.68 | | 1 | |
| CCS | O14618 | OID20973 | nonHDL | Wald ratio | 1 | 0.04 (0, 0.08) | 0.04 | 0.55 | | 1 | |
| CD200R1 | Q8TD46 | OID20595 | nonHDL | Wald ratio | 1 | -0.02 (-0.03, 0) | 0.01 | 0.37 | | 1 | |
| CD200R1 | Q8TD46 | OID20595 | TC | Wald ratio | 1 | -0.02 (-0.03, 0) | 0.03 | 0.51 | | 1 | |
| CD276 | Q5ZPR3 | OID20680 | TC | Wald ratio | 1 | 0.02 (0, 0.03) | 0.03 | 0.51 | | 1 | |
| CD276 | Q5ZPR3 | OID20680 | nonHDL | Wald ratio | 1 | 0.02 (0, 0.03) | 0.03 | 0.51 | | 1 | |
| CD300E | Q496F6 | OID21418 | HDL | Wald ratio | 1 | -0.04 (-0.07, -0.01) | 4.6E-03 | 0.12 | | 1 | |
| CD300E | Q496F6 | OID21418 | TC | Wald ratio | 1 | -0.03 (-0.06, 0) | 0.03 | 0.51 | | 1 | |
| CD300LF | Q8TDQ1 | OID21469 | HDL | Wald ratio | 1 | -0.01 (-0.03, 0) | 0.05 | 0.42 | | 1 | |
| CD302 | Q8IX05 | OID21328 | HDL | Wald ratio | 1 | -0.06 (-0.11, -0.01) | 0.02 | 0.26 | | 1 | |
| CD4 | P01730 | OID20584 | TG | Wald ratio | 1 | -0.07 (-0.13, -0.01) | 0.02 | 0.39 | | 1 | |
| CD4 | P01730 | OID20584 | LDL | Wald ratio | 1 | -0.07 (-0.13, -0.01) | 0.03 | 0.64 | | 1 | |
| CD4 | P01730 | OID20584 | nonHDL | Wald ratio | 1 | -0.06 (-0.12, -0.01) | 0.03 | 0.51 | | 1 | |
| CDA | P32320 | OID30345 | HDL | Wald ratio | 1 | 0.04 (0, 0.07) | 0.03 | 0.34 | | 1 | |
| CEACAM1 | P13688 | OID21528 | TG | Wald ratio | 1 | 0.05 (0, 0.1) | 0.05 | 0.54 | | 1 | |
| CELSR2 | Q9HCU4 | OID30593 | LDL | Wald ratio | 1 | -0.36 (-0.4, -0.33) | 6.0E-97 | 4.3E-94 | | 4.3E-94 | |
| CELSR2 | Q9HCU4 | OID30593 | nonHDL | Wald ratio | 1 | -0.34 (-0.38, -0.31) | 5.8E-81 | 4.1E-78 | | 4.1E-78 | |
| CELSR2 | Q9HCU4 | OID30593 | TC | Wald ratio | 1 | -0.3 (-0.34, -0.27) | 4.6E-68 | 3.3E-65 | | 3.3E-65 | |
| CELSR2 | Q9HCU4 | OID30593 | HDL | Wald ratio | 1 | 0.11 (0.08, 0.14) | 3.0E-10 | 5.2E-08 | | 2.1E-07 | |
| CELSR2 | Q9HCU4 | OID30593 | TG | Wald ratio | 1 | -0.05 (-0.08, -0.02) | 3.7E-03 | 0.16 | | 1 | |
| CFHR2 | P36980 | OID30720 | TG | Inverse variance weighted | 2 | 0.03 (0, 0.05) | 0.02 | 0.43 | | 1 | |
| CFHR4 | Q92496 | OID30758 | TG | Wald ratio | 1 | 0.03 (0.01, 0.05) | 0.01 | 0.31 | | 1 | |
| CFHR5 | Q9BXR6 | OID30716 | TG | Wald ratio | 1 | 0.09 (0.04, 0.15) | 1.3E-03 | 0.09 | | 0.94 | |
| CGREF1 | Q99674 | OID20152 | TG | Wald ratio | 1 | -0.08 (-0.11, -0.04) | 1.9E-05 | 2.3E-03 | | 0.01 | |
| CLEC4M | Q9H2X3 | OID30405 | LDL | Wald ratio | 1 | -0.05 (-0.1, 0) | 0.05 | 0.72 | | 1 | |
| CLGN | O14967 | OID30957 | nonHDL | Wald ratio | 1 | 0.02 (0, 0.04) | 0.01 | 0.38 | | 1 | |
| CLGN | O14967 | OID30957 | TC | Wald ratio | 1 | 0.02 (0, 0.04) | 0.03 | 0.51 | | 1 | |
| CLMP | Q9H6B4 | OID21419 | HDL | Wald ratio | 1 | -0.06 (-0.11, -0.01) | 0.01 | 0.23 | | 1 | |
| CLPS | P04118 | OID21156 | TG | Wald ratio | 1 | 0.04 (0.01, 0.07) | 0.01 | 0.34 | | 1 | |
| CLSTN2 | Q9H4D0 | OID20664 | LDL | Wald ratio | 1 | -0.07 (-0.12, -0.03) | 1.2E-03 | 0.10 | | 0.82 | |
| CLSTN2 | Q9H4D0 | OID20664 | nonHDL | Wald ratio | 1 | -0.06 (-0.11, -0.02) | 6.0E-03 | 0.25 | | 1 | |
| CLSTN2 | Q9H4D0 | OID20664 | TC | Wald ratio | 1 | -0.06 (-0.1, -0.02) | 7.4E-03 | 0.25 | | 1 | |
| CNTN5 | O94779 | OID21006 | LDL | Wald ratio | 1 | -0.05 (-0.09, 0) | 0.03 | 0.64 | | 1 | |
| CRISP2 | P16562 | OID21456 | HDL | Wald ratio | 1 | 0.03 (0, 0.05) | 0.02 | 0.28 | | 1 | |
| CRYM | Q14894 | OID30815 | TC | Wald ratio | 1 | -0.04 (-0.08, -0.01) | 0.02 | 0.51 | | 1 | |
| CRYM | Q14894 | OID30815 | nonHDL | Wald ratio | 1 | -0.04 (-0.08, 0) | 0.04 | 0.55 | | 1 | |
| CST3 | P01034 | OID20400 | TG | Wald ratio | 1 | 0.05 (0, 0.1) | 0.04 | 0.53 | | 1 | |
| CTSD | P07339 | OID20358 | HDL | Wald ratio | 1 | -0.05 (-0.09, 0) | 0.04 | 0.42 | | 1 | |
| CYB5R2 | Q6BCY4 | OID31512 | LDL | Wald ratio | 1 | -0.05 (-0.09, 0) | 0.04 | 0.66 | | 1 | |
| DDX58 | O95786 | OID21226 | TG | Wald ratio | 1 | 0.05 (0.01, 0.09) | 0.02 | 0.43 | | 1 | |
| DLK1 | P80370 | OID20295 | TC | Wald ratio | 1 | -0.04 (-0.07, -0.01) | 4.5E-03 | 0.19 | | 1 | |
| DLK1 | P80370 | OID20295 | LDL | Wald ratio | 1 | -0.04 (-0.06, -0.01) | 7.2E-03 | 0.36 | | 1 | |
| DLK1 | P80370 | OID20295 | nonHDL | Wald ratio | 1 | -0.03 (-0.06, 0) | 0.03 | 0.50 | | 1 | |
| DPEP2 | Q9H4A9 | OID21305 | HDL | Wald ratio | 1 | -0.05 (-0.07, -0.02) | 6.0E-05 | 5.0E-03 | | 0.04 | |
| DPEP2 | Q9H4A9 | OID21305 | TG | Wald ratio | 1 | 0.03 (0.01, 0.05) | 0.01 | 0.31 | | 1 | |
| DPP7 | Q9UHL4 | OID20142 | HDL | Wald ratio | 1 | 0.04 (0, 0.07) | 0.03 | 0.38 | | 1 | |
| DRAXIN | Q8NBI3 | OID20917 | LDL | Wald ratio | 1 | 0.06 (0, 0.11) | 0.03 | 0.65 | | 1 | |
| ECM1 | Q16610 | OID30736 | LDL | Wald ratio | 1 | 0.03 (0, 0.05) | 0.03 | 0.64 | | 1 | |
| ENDOU | P21128 | OID31498 | HDL | Wald ratio | 1 | 0.04 (0, 0.08) | 0.04 | 0.42 | | 1 | |
| ENTPD6 | O75354 | OID20100 | nonHDL | Wald ratio | 1 | -0.06 (-0.12, 0) | 0.05 | 0.56 | | 1 | |
| ENTPD6 | O75354 | OID20100 | TG | Wald ratio | 1 | -0.06 (-0.11, 0) | 0.05 | 0.54 | | 1 | |
| EPPK1 | P58107 | OID30250 | LDL | Wald ratio | 1 | -0.09 (-0.13, -0.04) | 6.3E-05 | 7.4E-03 | | 0.04 | |
| EPPK1 | P58107 | OID30250 | nonHDL | Wald ratio | 1 | -0.09 (-0.13, -0.04) | 1.5E-04 | 0.01 | | 0.11 | |
| EPPK1 | P58107 | OID30250 | TC | Wald ratio | 1 | -0.08 (-0.12, -0.03) | 4.2E-04 | 0.03 | | 0.30 | |
| F11 | P03951 | OID30773 | LDL | Wald ratio | 1 | -0.05 (-0.09, -0.01) | 9.1E-03 | 0.36 | | 1 | |
| F11 | P03951 | OID30773 | TC | Wald ratio | 1 | -0.05 (-0.08, -0.01) | 0.02 | 0.46 | | 1 | |
| F13B | P05160 | OID30781 | TG | Wald ratio | 1 | 0.03 (0, 0.06) | 0.03 | 0.44 | | 1 | |
| FCER2 | P06734 | OID21133 | HDL | Inverse variance weighted | 2 | 0.04 (0.01, 0.07) | 3.9E-03 | 0.11 | | 1 | |
| FCER2 | P06734 | OID21133 | TG | Inverse variance weighted | 2 | -0.04 (-0.07, -0.01) | 4.9E-03 | 0.18 | | 1 | |
| FCER2 | P06734 | OID21133 | nonHDL | Inverse variance weighted | 2 | -0.04 (-0.07, -0.01) | 0.02 | 0.42 | | 1 | |
| FGFR4 | P22455 | OID30646 | TG | Wald ratio | 1 | -0.02 (-0.03, 0) | 0.03 | 0.44 | | 1 | |
| FGFR4 | P22455 | OID30646 | TC | Wald ratio | 1 | -0.02 (-0.03, 0) | 0.03 | 0.51 | | 1 | |
| FGFR4 | P22455 | OID30646 | nonHDL | Wald ratio | 1 | -0.02 (-0.03, 0) | 0.04 | 0.55 | | 1 | |
| FN1 | P02751 | OID30787 | nonHDL | Wald ratio | 1 | 0.04 (0.01, 0.07) | 0.01 | 0.37 | | 1 | |
| FN1 | P02751 | OID30787 | TG | Wald ratio | 1 | 0.03 (0, 0.06) | 0.04 | 0.53 | | 1 | |
| FUT8 | Q9BYC5 | OID20992 | TC | Wald ratio | 1 | -0.02 (-0.04, 0) | 0.04 | 0.53 | | 1 | |
| GALNT10 | Q86SR1 | OID21371 | TG | Wald ratio | 1 | -0.05 (-0.09, -0.01) | 0.01 | 0.31 | | 1 | |
| GALNT10 | Q86SR1 | OID21371 | nonHDL | Wald ratio | 1 | -0.05 (-0.09, -0.01) | 0.03 | 0.50 | | 1 | |
| GALNT10 | Q86SR1 | OID21371 | TC | Wald ratio | 1 | -0.04 (-0.08, 0) | 0.04 | 0.51 | | 1 | |
| GALNT3 | Q14435 | OID20471 | HDL | Wald ratio | 1 | 0.05 (0, 0.1) | 0.05 | 0.44 | | 1 | |
| GAS6 | Q14393 | OID20318 | nonHDL | Wald ratio | 1 | -0.09 (-0.13, -0.05) | 7.2E-06 | 8.4E-04 | | 5.1E-03 | |
| GAS6 | Q14393 | OID20318 | TC | Wald ratio | 1 | -0.08 (-0.12, -0.04) | 3.4E-05 | 4.0E-03 | | 0.02 | |
| GAS6 | Q14393 | OID20318 | TG | Wald ratio | 1 | -0.08 (-0.11, -0.04) | 1.6E-04 | 0.01 | | 0.11 | |
| GAS6 | Q14393 | OID20318 | LDL | Wald ratio | 1 | -0.06 (-0.1, -0.02) | 2.7E-03 | 0.17 | | 1 | |
| GASK1A | Q9UFP1 | OID30109 | nonHDL | Inverse variance weighted | 2 | -0.01 (-0.03, 0) | 0.03 | 0.50 | | 1 | |
| GASK1A | Q9UFP1 | OID30109 | TC | Inverse variance weighted | 2 | -0.02 (-0.03, 0) | 0.03 | 0.51 | | 1 | |
| GDF15 | Q99988 | OID20251 | HDL | Wald ratio | 1 | -0.06 (-0.11, -0.01) | 0.02 | 0.31 | | 1 | |
| GIPC3 | Q8TF64 | OID31507 | HDL | Wald ratio | 1 | -0.09 (-0.14, -0.04) | 7.6E-04 | 0.04 | | 0.54 | |
| GRIK2 | Q13002 | OID30977 | nonHDL | Wald ratio | 1 | -0.07 (-0.11, -0.02) | 2.5E-03 | 0.14 | | 1 | |
| GRIK2 | Q13002 | OID30977 | TC | Wald ratio | 1 | -0.06 (-0.1, -0.02) | 7.3E-03 | 0.25 | | 1 | |
| GRIK2 | Q13002 | OID30977 | LDL | Wald ratio | 1 | -0.05 (-0.09, -0.01) | 0.02 | 0.55 | | 1 | |
| GRP | P07492 | OID30264 | TC | Wald ratio | 1 | -0.04 (-0.08, 0) | 0.05 | 0.57 | | 1 | |
| GSTA1 | P08263 | OID20166 | TG | Wald ratio | 1 | -0.09 (-0.13, -0.05) | 5.4E-05 | 4.2E-03 | | 0.04 | |
| GSTA1 | P08263 | OID20166 | HDL | Wald ratio | 1 | 0.07 (0.03, 0.11) | 1.8E-03 | 0.07 | | 1 | |
| GSTA1 | P08263 | OID20166 | nonHDL | Wald ratio | 1 | -0.05 (-0.1, -0.01) | 0.02 | 0.44 | | 1 | |
| GSTA3 | Q16772 | OID21242 | TG | Wald ratio | 1 | -0.08 (-0.11, -0.04) | 5.4E-05 | 4.2E-03 | | 0.04 | |
| GSTA3 | Q16772 | OID21242 | HDL | Wald ratio | 1 | 0.06 (0.02, 0.1) | 1.8E-03 | 0.07 | | 1 | |
| GSTA3 | Q16772 | OID21242 | nonHDL | Wald ratio | 1 | -0.05 (-0.08, -0.01) | 0.02 | 0.44 | | 1 | |
| GSTM4 | Q03013 | OID30164 | HDL | Wald ratio | 1 | -0.11 (-0.16, -0.06) | 9.4E-06 | 9.5E-04 | | 6.6E-03 | |
| GSTT2B | P0CG30 | OID31097 | HDL | Wald ratio | 1 | 0.02 (0, 0.03) | 0.01 | 0.24 | | 1 | |
| HAVCR1 | Q96D42 | OID21422 | nonHDL | Wald ratio | 1 | 0.04 (0.01, 0.07) | 0.01 | 0.37 | | 1 | |
| HAVCR1 | Q96D42 | OID21422 | TC | Wald ratio | 1 | 0.03 (0, 0.06) | 0.05 | 0.57 | | 1 | |
| HBZ | P02008 | OID30307 | TC | Wald ratio | 1 | -0.02 (-0.03, -0.01) | 2.7E-03 | 0.15 | | 1 | |
| HBZ | P02008 | OID30307 | nonHDL | Wald ratio | 1 | -0.01 (-0.03, 0) | 0.03 | 0.50 | | 1 | |
| HBZ | P02008 | OID30307 | HDL | Wald ratio | 1 | -0.01 (-0.03, 0) | 0.04 | 0.42 | | 1 | |
| HBZ | P02008 | OID30307 | LDL | Wald ratio | 1 | -0.01 (-0.03, 0) | 0.05 | 0.71 | | 1 | |
| HDGF | P51858 | OID21455 | TC | Wald ratio | 1 | -0.01 (-0.03, 0) | 0.04 | 0.56 | | 1 | |
| HDGF | P51858 | OID21455 | TG | Wald ratio | 1 | -0.01 (-0.03, 0) | 0.05 | 0.54 | | 1 | |
| HGFAC | Q04756 | OID30718 | TC | Wald ratio | 1 | -0.07 (-0.11, -0.04) | 6.1E-05 | 6.1E-03 | | 0.04 | |
| HGFAC | Q04756 | OID30718 | nonHDL | Wald ratio | 1 | -0.07 (-0.11, -0.04) | 9.3E-05 | 9.4E-03 | | 0.07 | |
| HGFAC | Q04756 | OID30718 | LDL | Wald ratio | 1 | -0.07 (-0.1, -0.03) | 1.9E-04 | 0.02 | | 0.14 | |
| HSBP1 | O75506 | OID30232 | nonHDL | Wald ratio | 1 | 0.05 (0.01, 0.08) | 7.6E-03 | 0.28 | | 1 | |
| HSBP1 | O75506 | OID30232 | TC | Wald ratio | 1 | 0.04 (0, 0.07) | 0.03 | 0.51 | | 1 | |
| HSBP1 | O75506 | OID30232 | LDL | Wald ratio | 1 | 0.04 (0, 0.07) | 0.04 | 0.65 | | 1 | |
| IDO1 | P14902 | OID30230 | LDL | Wald ratio | 1 | -0.05 (-0.09, 0) | 0.04 | 0.65 | | 1 | |
| IDO1 | P14902 | OID30563 | LDL | Wald ratio | 1 | -0.05 (-0.1, 0) | 0.04 | 0.65 | | 1 | |
| IDO1 | P14902 | OID31050 | LDL | Wald ratio | 1 | -0.05 (-0.09, 0) | 0.04 | 0.65 | | 1 | |
| IDO1 | P14902 | OID31474 | LDL | Wald ratio | 1 | -0.05 (-0.1, 0) | 0.04 | 0.65 | | 1 | |
| IFNGR2 | P38484 | OID20941 | nonHDL | Wald ratio | 1 | -0.01 (-0.02, 0) | 0.05 | 0.58 | | 1 | |
| IGF2R | P11717 | OID21146 | nonHDL | Wald ratio | 1 | -0.05 (-0.08, -0.02) | 3.4E-03 | 0.17 | | 1 | |
| IGF2R | P11717 | OID21146 | LDL | Wald ratio | 1 | -0.05 (-0.08, -0.02) | 3.8E-03 | 0.22 | | 1 | |
| IGF2R | P11717 | OID21146 | TC | Wald ratio | 1 | -0.04 (-0.08, -0.01) | 7.5E-03 | 0.25 | | 1 | |
| IGFBPL1 | Q8WX77 | OID20155 | TG | Inverse variance weighted | 3 | 0.03 (0.01, 0.05) | 0.01 | 0.31 | | 1 | |
| IL12B | P29460 | OID20666 | TC | Wald ratio | 1 | 0.04 (0, 0.08) | 0.03 | 0.51 | | 1 | |
| IL17D | Q8TAD2 | OID20481 | TG | Wald ratio | 1 | 0.04 (0, 0.08) | 0.03 | 0.48 | | 1 | |
| IL1R2 | P27930 | OID20757 | TG | Wald ratio | 1 | -0.04 (-0.07, 0) | 0.04 | 0.52 | | 1 | |
| IL1RN | P18510 | OID20700 | HDL | Wald ratio | 1 | 0.06 (0, 0.11) | 0.05 | 0.42 | | 1 | |
| IL2RA | P01589 | OID20267 | TC | Wald ratio | 1 | 0.04 (0, 0.07) | 0.03 | 0.51 | | 1 | |
| INHBB | P09529 | OID30625 | nonHDL | Wald ratio | 1 | -0.1 (-0.15, -0.04) | 1.2E-03 | 0.07 | | 0.84 | |
| INHBB | P09529 | OID30625 | TC | Wald ratio | 1 | -0.08 (-0.14, -0.03) | 3.1E-03 | 0.15 | | 1 | |
| INHBB | P09529 | OID30625 | LDL | Wald ratio | 1 | -0.08 (-0.13, -0.02) | 7.9E-03 | 0.36 | | 1 | |
| INHBB | P09529 | OID30625 | HDL | Wald ratio | 1 | 0.07 (0.02, 0.13) | 8.9E-03 | 0.19 | | 1 | |
| INHBB | P09529 | OID30625 | TG | Wald ratio | 1 | -0.07 (-0.13, -0.02) | 0.01 | 0.31 | | 1 | |
| INHBC | P55103 | OID21093 | HDL | Wald ratio | 1 | -0.06 (-0.1, -0.02) | 2.1E-03 | 0.07 | | 1 | |
| ISLR2 | Q6UXK2 | OID20889 | LDL | Wald ratio | 1 | 0.06 (0.01, 0.11) | 0.02 | 0.57 | | 1 | |
| ITGA2 | P17301 | OID30570 | HDL | Wald ratio | 1 | 0.04 (0.01, 0.07) | 4.7E-03 | 0.12 | | 1 | |
| ITPA | Q9BY32 | OID30082 | HDL | Wald ratio | 1 | -0.02 (-0.03, 0) | 0.04 | 0.41 | | 1 | |
| KHK | P50053 | OID30241 | TG | Wald ratio | 1 | 0.06 (0.03, 0.1) | 4.1E-05 | 4.1E-03 | | 0.03 | |
| KIAA0319 | Q5VV43 | OID31094 | TG | Wald ratio | 1 | -0.05 (-0.1, -0.01) | 0.02 | 0.43 | | 1 | |
| KIR3DL1 | P43629 | OID21185 | nonHDL | Inverse variance weighted | 2 | -0.02 (-0.03, 0) | 0.04 | 0.52 | | 1 | |
| KLK4 | Q9Y5K2 | OID21391 | HDL | Wald ratio | 1 | -0.04 (-0.07, 0) | 0.03 | 0.39 | | 1 | |
| LECT2 | O14960 | OID30320 | HDL | Wald ratio | 1 | 0.02 (0.01, 0.04) | 0.01 | 0.21 | | 1 | |
| LILRA3 | Q8N6C8 | OID30376 | HDL | Wald ratio | 1 | -0.06 (-0.07, -0.04) | 5.0E-10 | 7.1E-08 | | 3.6E-07 | |
| LILRA3 | Q8N6C8 | OID30376 | LDL | Wald ratio | 1 | -0.02 (-0.04, 0) | 0.02 | 0.64 | | 1 | |
| LILRA3 | Q8N6C8 | OID30376 | TC | Wald ratio | 1 | -0.02 (-0.04, 0) | 0.03 | 0.51 | | 1 | |
| LILRA5 | A6NI73 | OID20209 | HDL | Wald ratio | 1 | -0.1 (-0.14, -0.06) | 8.8E-06 | 9.5E-04 | | 6.2E-03 | |
| LILRA5 | A6NI73 | OID20209 | TG | Wald ratio | 1 | 0.05 (0.01, 0.09) | 0.02 | 0.42 | | 1 | |
| LILRB1 | Q8NHL6 | OID20323 | TC | Inverse variance weighted | 2 | -0.03 (-0.06, 0) | 0.02 | 0.51 | | 1 | |
| LILRB2 | Q8N423 | OID20301 | HDL | Wald ratio | 1 | -0.06 (-0.08, -0.04) | 1.8E-11 | 4.3E-09 | | 1.3E-08 | |
| LILRB2 | Q8N423 | OID20301 | TC | Wald ratio | 1 | -0.02 (-0.04, 0) | 0.04 | 0.51 | | 1 | |
| LILRB5 | O75023 | OID20324 | HDL | Wald ratio | 1 | 0.03 (0.02, 0.05) | 6.4E-05 | 5.0E-03 | | 0.05 | |
| LILRB5 | O75023 | OID20324 | TC | Wald ratio | 1 | 0.03 (0.01, 0.04) | 1.8E-03 | 0.11 | | 1 | |
| LILRB5 | O75023 | OID20324 | LDL | Wald ratio | 1 | 0.02 (0.01, 0.03) | 4.2E-03 | 0.23 | | 1 | |
| LILRB5 | O75023 | OID20324 | nonHDL | Wald ratio | 1 | 0.02 (0, 0.04) | 0.02 | 0.50 | | 1 | |
| LPA | P08519 | OID30747 | LDL | Wald ratio | 1 | 0.1 (0.06, 0.15) | 5.2E-06 | 9.3E-04 | | 3.7E-03 | |
| LPA | P08519 | OID30747 | TC | Wald ratio | 1 | 0.08 (0.04, 0.13) | 2.5E-04 | 0.02 | | 0.18 | |
| LPA | P08519 | OID30747 | nonHDL | Wald ratio | 1 | 0.08 (0.03, 0.12) | 1.1E-03 | 0.07 | | 0.78 | |
| LYPD3 | O95274 | OID21530 | TG | Wald ratio | 1 | -0.05 (-0.09, -0.01) | 0.02 | 0.40 | | 1 | |
| MDGA1 | Q8NFP4 | OID20951 | TC | Inverse variance weighted | 2 | -0.01 (-0.03, 0) | 0.01 | 0.35 | | 1 | |
| MEGF9 | Q9H1U4 | OID20285 | TG | Wald ratio | 1 | 0.05 (0.01, 0.08) | 0.02 | 0.40 | | 1 | |
| MEP1B | Q16820 | OID20168 | HDL | Wald ratio | 1 | -0.02 (-0.03, 0) | 0.01 | 0.21 | | 1 | |
| MFGE8 | Q08431 | OID21134 | TG | Wald ratio | 1 | -0.06 (-0.1, -0.01) | 0.01 | 0.34 | | 1 | |
| MIA | Q16674 | OID21531 | TG | Wald ratio | 1 | 0.03 (0, 0.05) | 0.04 | 0.52 | | 1 | |
| MLN | P12872 | OID20541 | TG | Wald ratio | 1 | 0.04 (0, 0.08) | 0.04 | 0.52 | | 1 | |
| MLN | P12872 | OID20541 | HDL | Wald ratio | 1 | -0.04 (-0.08, 0) | 0.04 | 0.42 | | 1 | |
| MMP12 | P39900 | OID21439 | TC | Wald ratio | 1 | -0.04 (-0.07, 0) | 0.03 | 0.51 | | 1 | |
| MMP12 | P39900 | OID21439 | LDL | Wald ratio | 1 | -0.03 (-0.06, 0) | 0.05 | 0.71 | | 1 | |
| MSR1 | P21757 | OID21063 | HDL | Wald ratio | 1 | -0.07 (-0.13, -0.01) | 0.02 | 0.28 | | 1 | |
| MTHFSD | Q2M296 | OID31305 | HDL | Wald ratio | 1 | -0.03 (-0.06, -0.01) | 3.0E-03 | 0.10 | | 1 | |
| NAAA | Q02083 | OID20931 | HDL | Wald ratio | 1 | 0.04 (0.01, 0.07) | 0.02 | 0.28 | | 1 | |
| NCAM1 | P13591 | OID20354 | TG | Wald ratio | 1 | -0.06 (-0.1, -0.01) | 0.01 | 0.31 | | 1 | |
| NCAM1 | P13591 | OID20354 | nonHDL | Wald ratio | 1 | -0.05 (-0.1, 0) | 0.03 | 0.51 | | 1 | |
| NCAM2 | O15394 | OID21095 | HDL | Wald ratio | 1 | 0.05 (0, 0.1) | 0.03 | 0.39 | | 1 | |
| NCR3LG1 | Q68D85 | OID31490 | TG | Wald ratio | 1 | -0.08 (-0.13, -0.02) | 6.8E-03 | 0.23 | | 1 | |
| NCR3LG1 | Q68D85 | OID31490 | nonHDL | Wald ratio | 1 | -0.07 (-0.12, -0.01) | 0.03 | 0.50 | | 1 | |
| NFASC | O94856 | OID20634 | TG | Wald ratio | 1 | 0.06 (0.01, 0.11) | 0.03 | 0.46 | | 1 | |
| NID2 | Q14112 | OID21085 | HDL | Wald ratio | 1 | 0.04 (0, 0.08) | 0.04 | 0.41 | | 1 | |
| NPTXR | O95502 | OID20191 | LDL | Inverse variance weighted | 2 | -0.04 (-0.08, 0) | 0.03 | 0.64 | | 1 | |
| NRCAM | Q92823 | OID20229 | TG | Wald ratio | 1 | 0.03 (0, 0.07) | 0.04 | 0.53 | | 1 | |
| NRP1 | O14786 | OID20390 | HDL | Wald ratio | 1 | 0.06 (0.01, 0.11) | 0.01 | 0.23 | | 1 | |
| NT5E | P21589 | OID21498 | HDL | Wald ratio | 1 | -0.03 (-0.05, 0) | 0.03 | 0.36 | | 1 | |
| NUCB2 | P80303 | OID21448 | nonHDL | Wald ratio | 1 | -0.06 (-0.1, -0.03) | 8.7E-04 | 0.06 | | 0.62 | |
| NUCB2 | P80303 | OID21448 | TC | Wald ratio | 1 | -0.05 (-0.09, -0.02) | 3.2E-03 | 0.15 | | 1 | |
| NUCB2 | P80303 | OID21448 | LDL | Wald ratio | 1 | -0.05 (-0.08, -0.01) | 9.2E-03 | 0.36 | | 1 | |
| NUCB2 | P80303 | OID21448 | TG | Wald ratio | 1 | -0.04 (-0.08, -0.01) | 0.02 | 0.43 | | 1 | |
| PCSK7 | Q16549 | OID31410 | TG | Wald ratio | 1 | -0.14 (-0.18, -0.11) | 1.6E-15 | 2.8E-13 | | 1.1E-12 | |
| PCSK7 | Q16549 | OID31410 | TC | Wald ratio | 1 | -0.05 (-0.08, -0.01) | 8.0E-03 | | 0.26 | | 1 |
| PCSK7 | Q16549 | OID31410 | nonHDL | Wald ratio | 1 | -0.04 (-0.08, 0) | 0.03 | | 0.50 | | 1 |
| PCSK9 | Q8NBP7 | OID20235 | LDL | Wald ratio | 1 | 0.16 (0.11, 0.21) | 5.2E-09 | | 1.2E-06 | | 3.7E-06 |
| PCSK9 | Q8NBP7 | OID20235 | nonHDL | Wald ratio | 1 | 0.13 (0.08, 0.19) | 2.9E-06 | | 4.0E-04 | | 2.0E-03 |
| PCSK9 | Q8NBP7 | OID20235 | TC | Wald ratio | 1 | 0.12 (0.07, 0.18) | 6.1E-06 | | 8.7E-04 | | 4.3E-03 |
| PDGFRB | P09619 | OID20268 | TC | Wald ratio | 1 | 0.02 (0, 0.03) | 0.01 | | 0.35 | | 1 |
| PDGFRB | P09619 | OID20268 | nonHDL | Wald ratio | 1 | 0.01 (0, 0.03) | 0.05 | | 0.58 | | 1 |
| PECR | Q9BY49 | OID30228 | HDL | Wald ratio | 1 | -0.05 (-0.08, -0.02) | 1.0E-03 | | 0.05 | | 0.72 |
| PENK | P01210 | OID30666 | TG | Wald ratio | 1 | -0.05 (-0.09, 0) | 0.03 | | 0.48 | | 1 |
| PER3 | P56645 | OID30511 | HDL | Wald ratio | 1 | -0.05 (-0.1, 0) | 0.04 | | 0.39 | | 1 |
| PGLYRP1 | O75594 | OID20338 | nonHDL | Wald ratio | 1 | 0.05 (0, 0.11) | 0.04 | | 0.55 | | 1 |
| PGLYRP2 | Q96PD5 | OID30742 | LDL | Wald ratio | 1 | 0.02 (0, 0.03) | 0.02 | | 0.57 | | 1 |
| PGLYRP2 | Q96PD5 | OID30742 | nonHDL | Wald ratio | 1 | 0.02 (0, 0.03) | 0.03 | | 0.50 | | 1 |
| PI16 | Q6UXB8 | OID30676 | HDL | Wald ratio | 1 | 0.06 (0.02, 0.11) | 8.8E-03 | | 0.19 | | 1 |
| PINLYP | A6NC86 | OID30651 | HDL | Wald ratio | 1 | -0.02 (-0.04, 0) | 0.02 | | 0.28 | | 1 |
| PLA2G15 | Q8NCC3 | OID21473 | HDL | Wald ratio | 1 | -0.2 (-0.25, -0.15) | 2.3E-13 | | 8.2E-11 | | 1.6E-10 |
| PLA2G15 | Q8NCC3 | OID21473 | TG | Wald ratio | 1 | 0.06 (0.01, 0.11) | 0.02 | | 0.43 | | 1 |
| PLA2G15 | Q8NCC3 | OID21473 | TC | Wald ratio | 1 | -0.06 (-0.11, 0) | 0.04 | | 0.51 | | 1 |
| PLTP | P55058 | OID20238 | TG | Wald ratio | 1 | -0.11 (-0.14, -0.07) | 3.9E-10 | | 5.5E-08 | | 2.8E-07 |
| PLTP | P55058 | OID20238 | nonHDL | Wald ratio | 1 | -0.05 (-0.08, -0.02) | 4.4E-03 | | 0.20 | | 1 |
| PLTP | P55058 | OID20238 | HDL | Wald ratio | 1 | 0.04 (0.01, 0.07) | 0.02 | | 0.26 | | 1 |
| PON2 | Q15165 | OID20088 | LDL | Wald ratio | 1 | 0.01 (0, 0.03) | 0.04 | | 0.71 | | 1 |
| PPCDC | Q96CD2 | OID21027 | HDL | Wald ratio | 1 | -0.03 (-0.05, 0) | 0.04 | | 0.42 | | 1 |
| PRELP | P51888 | OID20766 | TG | Inverse variance weighted | 2 | -0.03 (-0.06, 0) | 0.03 | | 0.48 | | 1 |
| PROK1 | P58294 | OID20543 | nonHDL | Wald ratio | 1 | 0.04 (0.01, 0.07) | 0.02 | | 0.42 | | 1 |
| PROK1 | P58294 | OID20543 | LDL | Wald ratio | 1 | 0.03 (0, 0.07) | 0.03 | | 0.64 | | 1 |
| PRSS53 | Q2L4Q9 | OID31093 | LDL | Wald ratio | 1 | 0.02 (0, 0.03) | 0.03 | | 0.64 | | 1 |
| PSMD9 | O00233 | OID21304 | HDL | Wald ratio | 1 | -0.07 (-0.12, -0.02) | 8.2E-03 | | 0.19 | | 1 |
| PTGR1 | Q14914 | OID30297 | HDL | Wald ratio | 1 | -0.05 (-0.1, -0.01) | 0.02 | | 0.28 | | 1 |
| REG3G | Q6UW15 | OID31408 | TG | Wald ratio | 1 | 0.04 (0.01, 0.07) | 0.01 | | 0.34 | | 1 |
| RET | P07949 | OID21346 | HDL | Wald ratio | 1 | 0.08 (0.03, 0.13) | 1.3E-03 | | 0.06 | | 0.95 |
| RGMA | Q96B86 | OID21065 | HDL | Wald ratio | 1 | -0.05 (-0.1, 0) | 0.03 | | 0.39 | | 1 |
| RNASE1 | P07998 | OID30672 | HDL | Wald ratio | 1 | 0.05 (0, 0.1) | 0.04 | | 0.42 | | 1 |
| RNASE10 | Q5GAN6 | OID30238 | TG | Wald ratio | 1 | -0.02 (-0.04, -0.01) | 0.01 | | 0.31 | | 1 |
| RNASE4 | P34096 | OID30705 | TC | Wald ratio | 1 | 0.1 (0.03, 0.17) | 4.9E-03 | | 0.19 | | 1 |
| RNASE4 | P34096 | OID30705 | LDL | Wald ratio | 1 | 0.08 (0.01, 0.14) | 0.02 | | 0.54 | | 1 |
| RNASE4 | P34096 | OID30705 | nonHDL | Wald ratio | 1 | 0.08 (0.01, 0.16) | 0.03 | | 0.51 | | 1 |
| S100P | P25815 | OID20103 | nonHDL | Wald ratio | 1 | 0.05 (0.01, 0.1) | 0.02 | | 0.49 | | 1 |
| SAT2 | Q96F10 | OID31306 | TC | Wald ratio | 1 | 0.03 (0.01, 0.05) | 4.2E-03 | | 0.19 | | 1 |
| SAT2 | Q96F10 | OID31306 | nonHDL | Wald ratio | 1 | 0.03 (0.01, 0.05) | 6.5E-03 | | 0.25 | | 1 |
| SAT2 | Q96F10 | OID31306 | LDL | Wald ratio | 1 | 0.03 (0.01, 0.05) | 8.4E-03 | | 0.36 | | 1 |
| SCARA5 | Q6ZMJ2 | OID21058 | nonHDL | Wald ratio | 1 | -0.06 (-0.11, 0) | 0.04 | | 0.55 | | 1 |
| SCN4B | Q8IWT1 | OID30277 | HDL | Wald ratio | 1 | 0.03 (0, 0.06) | 0.02 | | 0.30 | | 1 |
| SERPINA11 | Q86U17 | OID20281 | HDL | Wald ratio | 1 | -0.02 (-0.04, 0) | 0.04 | | 0.42 | | 1 |
| SERPINE2 | P07093 | OID30359 | TG | Wald ratio | 1 | -0.06 (-0.1, -0.02) | 3.0E-03 | | 0.16 | | 1 |
| SERPINE2 | P07093 | OID30359 | HDL | Wald ratio | 1 | 0.05 (0.01, 0.09) | 9.1E-03 | | 0.19 | | 1 |
| SERPINE2 | P07093 | OID30359 | nonHDL | Wald ratio | 1 | -0.04 (-0.08, 0) | 0.04 | | 0.55 | | 1 |
| SIGLEC9 | Q9Y336 | OID21390 | LDL | Wald ratio | 1 | -0.03 (-0.05, 0) | 0.04 | | 0.67 | | 1 |
| SIRPB1 | O00241 | OID20739 | TG | Wald ratio | 1 | -0.05 (-0.08, -0.02) | 3.1E-03 | | 0.16 | | 1 |
| SIRPB1 | O00241 | OID20739 | nonHDL | Wald ratio | 1 | -0.04 (-0.07, -0.01) | 0.01 | | 0.38 | | 1 |
| SIRPB1 | O00241 | OID20739 | TC | Wald ratio | 1 | -0.04 (-0.07, -0.01) | 0.02 | | 0.49 | | 1 |
| SIRPB1 | O00241 | OID20739 | LDL | Wald ratio | 1 | -0.03 (-0.06, 0) | 0.03 | | 0.64 | | 1 |
| SMOC2 | Q9H3U7 | OID20730 | HDL | Wald ratio | 1 | -0.07 (-0.11, -0.02) | 3.6E-03 | | 0.11 | | 1 |
| SMOC2 | Q9H3U7 | OID20730 | TG | Wald ratio | 1 | 0.05 (0.01, 0.09) | 0.03 | | 0.44 | | 1 |
| SPARCL1 | Q14515 | OID20361 | HDL | Inverse variance weighted | 2 | -0.02 (-0.04, 0) | 0.03 | | 0.36 | | 1 |
| SPARCL1 | Q14515 | OID20361 | TG | Inverse variance weighted | 2 | 0.02 (0, 0.04) | 0.04 | | 0.52 | | 1 |
| SSC5D | A1L4H1 | OID20351 | TC | Wald ratio | 1 | 0.05 (0.01, 0.08) | 0.03 | | 0.51 | | 1 |
| STAB2 | Q8WWQ8 | OID30401 | TG | Wald ratio | 1 | -0.06 (-0.12, -0.01) | 0.03 | | 0.44 | | 1 |
| SUSD2 | Q9UGT4 | OID21098 | TC | Wald ratio | 1 | 0.04 (0, 0.08) | 0.04 | | 0.51 | | 1 |
| TEK | Q02763 | OID21496 | nonHDL | Wald ratio | 1 | 0.03 (0, 0.06) | 0.04 | | 0.52 | | 1 |
| TEK | Q02763 | OID21496 | TG | Wald ratio | 1 | 0.03 (0, 0.06) | 0.04 | | 0.53 | | 1 |
| TF | P02787 | OID30783 | HDL | Wald ratio | 1 | -0.05 (-0.08, -0.02) | 2.5E-03 | | 0.08 | | 1 |
| TF | P02787 | OID30783 | TC | Wald ratio | 1 | -0.03 (-0.06, 0) | 0.05 | | 0.58 | | 1 |
| TFPI | P10646 | OID20388 | TG | Wald ratio | 1 | -0.04 (-0.09, 0) | 0.03 | | 0.48 | | 1 |
| TGFBI | Q15582 | OID20417 | HDL | Wald ratio | 1 | -0.03 (-0.05, -0.01) | 3.8E-03 | | 0.11 | | 1 |
| TGFBI | Q15582 | OID20417 | nonHDL | Wald ratio | 1 | 0.02 (0, 0.05) | 0.03 | | 0.50 | | 1 |
| TGOLN2 | O43493 | OID30691 | HDL | Wald ratio | 1 | -0.03 (-0.06, -0.01) | 3.3E-03 | | 0.10 | | 1 |
| THOP1 | P52888 | OID20194 | HDL | Wald ratio | 1 | 0.08 (0.03, 0.12) | 8.8E-04 | | 0.04 | | 0.62 |
| TIMP4 | Q99727 | OID21147 | TG | Wald ratio | 1 | 0.04 (0.01, 0.07) | 6.0E-03 | | 0.21 | | 1 |
| TLR3 | O15455 | OID20612 | HDL | Wald ratio | 1 | -0.03 (-0.05, -0.01) | 7.3E-04 | | 0.04 | | 0.51 |
| TMPRSS5 | Q9H3S3 | OID20947 | nonHDL | Wald ratio | 1 | -0.02 (-0.04, 0) | 0.03 | | 0.51 | | 1 |
| TMPRSS5 | Q9H3S3 | OID20947 | TC | Wald ratio | 1 | -0.02 (-0.04, 0) | 0.05 | | 0.57 | | 1 |
| TNFSF13 | O75888 | OID20733 | nonHDL | Wald ratio | 1 | -0.1 (-0.16, -0.05) | 1.4E-04 | | 0.01 | | 0.10 |
| TNFSF13 | O75888 | OID20733 | TC | Wald ratio | 1 | -0.09 (-0.15, -0.04) | 2.6E-04 | | 0.02 | | 0.18 |
| TNFSF13 | O75888 | OID20733 | LDL | Wald ratio | 1 | -0.08 (-0.13, -0.03) | 1.6E-03 | | 0.13 | | 1 |
| TPK1 | Q9H3S4 | OID30338 | TG | Wald ratio | 1 | 0.06 (0.02, 0.09) | 4.4E-03 | | 0.17 | | 1 |
| TYRO3 | Q06418 | OID20161 | HDL | Wald ratio | 1 | -0.03 (-0.06, -0.01) | 0.01 | | 0.23 | | 1 |
| UMOD | P07911 | OID20237 | HDL | Inverse variance weighted | 2 | -0.02 (-0.04, 0) | 0.05 | | 0.42 | | 1 |
| VEGFA | P15692 | OID20650 | TG | Wald ratio | 1 | 0.04 (0.01, 0.06) | 3.4E-03 | | 0.16 | | 1 |
| VEGFA | P15692 | OID20650 | nonHDL | Wald ratio | 1 | 0.03 (0.01, 0.06) | 0.01 | | 0.38 | | 1 |
| VNN1 | O95497 | OID30711 | LDL | Inverse variance weighted | 3 | 0.01 (0, 0.03) | 0.05 | | 0.71 | | 1 |
| VSIG10L | Q86VR7 | OID30189 | HDL | Wald ratio | 1 | -0.07 (-0.13, -0.02) | 7.2E-03 | | 0.17 | | 1 |
| VSTM1 | Q6UX27 | OID20851 | LDL | Wald ratio | 1 | 0.03 (0.01, 0.05) | 9.6E-03 | | 0.36 | | 1 |
| VSTM1 | Q6UX27 | OID20851 | nonHDL | Wald ratio | 1 | 0.03 (0.01, 0.06) | 0.02 | | 0.40 | | 1 |
| VSTM1 | Q6UX27 | OID20851 | TC | Wald ratio | 1 | 0.03 (0, 0.05) | 0.04 | | 0.51 | | 1 |
| VWC2 | Q2TAL6 | OID20936 | TG | Wald ratio | 1 | 0.03 (0, 0.06) | 0.03 | | 0.48 | | 1 |
| WFIKKN1 | Q96NZ8 | OID20939 | TC | Wald ratio | 1 | 0.06 (0.01, 0.12) | 0.03 | | 0.51 | | 1 |
| WFIKKN1 | Q96NZ8 | OID20939 | nonHDL | Wald ratio | 1 | 0.06 (0, 0.12) | 0.04 | | 0.55 | | 1 |

# **Supplementary Table S4. Results of Bayesian colocalisation**

| **Protein** | **UniProtID** | **ProbeID** | **Phenotype** | **N SNPs** | **PP.H0.abf** | **PP.H1.abf** | **PP.H2.abf** | **PP.H3.abf** | **PP.H4.abf** |
| --- | --- | --- | --- | --- | --- | --- | --- | --- | --- |
| APOE | P02649 | OID30727 | LDL | 2228 | 1.03E-65 | 1.40E-48 | 7.4E-18 | 1.00 | 7.79E-18 |
| KHK | P50053 | OID30241 | TG | 1232 | 6.30E-57 | 9.43E-45 | 6.7E-13 | 1.00 | 2.97E-14 |
| APOE | P02649 | OID30727 | TG | 2228 | 3.51E-60 | 4.75E-43 | 7.4E-18 | 1.00 | 5.23E-13 |
| LILRB5 | O75023 | OID20324 | HDL | 3003 | 7.00E-114 | 4.67E-14 | 1.5E-100 | 1.00 | 7.92E-13 |
| PCSK7 | Q16549 | OID31410 | TG | 2420 | 1.68E-235 | 4.81E-225 | 3.5E-11 | 1.00 | 1.26E-12 |
| CGREF1 | Q99674 | OID20152 | TG | 1249 | 7.54E-56 | 9.43E-45 | 8.0E-12 | 1.00 | 1.86E-12 |
| LILRA5 | A6NI73 | OID20209 | HDL | 2746 | 3.16E-20 | 4.68E-14 | 6.8E-07 | 1.00 | 5.16E-08 |
| DPEP2 | Q9H4A9 | OID21305 | HDL | 882 | 4.60E-27 | 2.12E-09 | 2.2E-18 | 1.00 | 1.96E-07 |
| LILRA3 | Q8N6C8 | OID30376 | HDL | 2796 | 9.44E-83 | 4.67E-14 | 2.0E-69 | 1.00 | 6.26E-07 |
| APOC1 | P02654 | OID30749 | HDL | 2226 | 1.50E-19 | 1.35E-14 | 1.1E-05 | 1.00 | 9.16E-07 |
| LILRB2 | Q8N423 | OID20301 | HDL | 2923 | 7.53E-70 | 4.66E-14 | 1.6E-56 | 1.00 | 1.27E-06 |
| APOC1 | P02654 | OID30749 | nonHDL | 2220 | 1.04E-27 | 9.40E-23 | 1.1E-05 | 1.00 | 3.33E-06 |
| APOC1 | P02654 | OID30749 | TC | 2233 | 1.06E-20 | 9.59E-16 | 1.1E-05 | 1.00 | 3.86E-06 |
| APOC1 | P02654 | OID30749 | TG | 2227 | 5.27E-48 | 4.75E-43 | 1.1E-05 | 1.00 | 8.98E-06 |
| GSTM4 | Q03013 | OID30164 | HDL | 1595 | 6.94E-10 | 5.70E-05 | 1.2E-05 | 1.00 | 4.71E-03 |
| ANGPTL4 | Q9BY76 | OID20703 | HDL | 2505 | 3.20E-05 | 0.23 | 6.0E-05 | 0.43 | 0.34 |
| THOP1 | P52888 | OID20194 | HDL | 3274 | 3.78E-06 | 0.53 | 6.6E-07 | 0.09 | 0.38 |
| GAS6 | Q14393 | OID20318 | nonHDL | 2629 | 4.05E-10 | 5.67E-03 | 3.3E-08 | 0.46 | 0.53 |
| ADAMTSL5 | Q6ZMM2 | OID30355 | TC | 2360 | 1.21E-03 | 0.33 | 3.1E-04 | 0.09 | 0.58 |
| TNFSF13 | O75888 | OID20733 | TC | 1912 | 1.47E-03 | 0.26 | 8.2E-04 | 0.15 | 0.59 |
| EPPK1 | P58107 | OID30250 | TC | 1562 | 8.39E-07 | 0.28 | 3.7E-07 | 0.12 | 0.60 |
| PECR | Q9BY49 | OID30228 | HDL | 2468 | 6.99E-15 | 0.34 | 1.1E-15 | 0.05 | 0.61 |
| GIPC3 | Q8TF64 | OID31507 | HDL | 2662 | 5.93E-04 | 0.26 | 2.8E-04 | 0.12 | 0.62 |
| TNFSF13 | O75888 | OID20733 | nonHDL | 1901 | 9.16E-04 | 0.16 | 9.6E-04 | 0.17 | 0.67 |
| TLR3 | O15455 | OID20612 | HDL | 3406 | 4.90E-82 | 0.30 | 2.2E-83 | 0.01 | 0.69 |
| GAS6 | Q14393 | OID20318 | TG | 2637 | 5.71E-09 | 0.08 | 1.4E-08 | 0.20 | 0.72 |
| ANGPTL3 | Q9Y5C1 | OID20407 | HDL | 2169 | 2.19E-06 | 0.14 | 1.9E-06 | 0.12 | 0.74 |
| EPPK1 | P58107 | OID30250 | nonHDL | 1556 | 3.30E-07 | 0.11 | 4.0E-07 | 0.13 | 0.76 |
| GSTA1 | P08263 | OID20166 | TG | 2249 | 3.25E-09 | 0.04 | 1.0E-08 | 0.14 | 0.82 |
| HGFAC | Q04756 | OID30718 | LDL | 2630 | 1.83E-12 | 0.17 | 1.2E-13 | 9.87E-03 | 0.82 |
| EPPK1 | P58107 | OID30250 | LDL | 1562 | 2.02E-07 | 0.07 | 3.4E-07 | 0.11 | 0.82 |
| GAS6 | Q14393 | OID20318 | TC | 2636 | 3.11E-09 | 0.04 | 9.5E-09 | 0.13 | 0.82 |
| LPA | P08519 | OID30747 | TC | 2860 | 3.71E-07 | 0.08 | 3.8E-07 | 0.09 | 0.83 |
| GSTA3 | Q16772 | OID21242 | TG | 2224 | 1.18E-10 | 0.04 | 3.6E-10 | 0.12 | 0.84 |
| PLA2G15 | Q8NCC3 | OID21473 | HDL | 1254 | 1.53E-13 | 3.04E-10 | 7.3E-05 | 0.14 | 0.86 |
| ANGPTL3 | Q9Y5C1 | OID20407 | TG | 2170 | 1.47E-30 | 9.56E-26 | 2.0E-06 | 0.13 | 0.87 |
| ANGPTL3 | Q9Y5C1 | OID20407 | LDL | 2170 | 2.81E-07 | 0.02 | 1.6E-06 | 0.11 | 0.88 |
| HGFAC | Q04756 | OID30718 | nonHDL | 2437 | 1.00E-12 | 0.09 | 7.0E-14 | 5.40E-03 | 0.90 |
| HGFAC | Q04756 | OID30718 | TC | 2627 | 7.08E-13 | 0.07 | 7.1E-14 | 5.64E-03 | 0.93 |
| ANGPTL3 | Q9Y5C1 | OID20407 | nonHDL | 2174 | 4.38E-15 | 2.86E-10 | 1.0E-06 | 0.07 | 0.93 |
| ANGPTL3 | Q9Y5C1 | OID20407 | TC | 2170 | 5.72E-20 | 3.73E-15 | 9.3E-07 | 0.06 | 0.94 |
| PCSK9 | Q8NBP7 | OID20235 | TC | 2741 | 1.26E-06 | 5.27E-03 | 3.3E-06 | 0.01 | 0.98 |
| PCSK9 | Q8NBP7 | OID20235 | LDL | 2741 | 6.03E-10 | 2.52E-06 | 3.7E-06 | 0.01 | 0.99 |
| PCSK9 | Q8NBP7 | OID20235 | nonHDL | 2739 | 8.15E-07 | 3.40E-03 | 2.4E-06 | 9.20E-03 | 0.99 |
| LPA | P08519 | OID30747 | LDL | 2860 | 7.51E-09 | 1.71E-03 | 4.9E-08 | 0.01 | 0.99 |
| APOE | P02649 | OID30727 | HDL | 2227 | 1.24E-33 | 1.68E-16 | 9.2E-20 | 0.01 | 0.99 |
| CELSR2 | Q9HCU4 | OID30593 | HDL | 1779 | 9.70E-17 | 6.36E-07 | 1.7E-12 | 0.01 | 0.99 |
| CELSR2 | Q9HCU4 | OID30593 | TC | 1778 | 1.16E-73 | 7.62E-64 | 6.4E-13 | 3.20E-03 | 1.00 |
| CELSR2 | Q9HCU4 | OID30593 | nonHDL | 1807 | 1.74E-86 | 1.14E-76 | 6.2E-13 | 3.09E-03 | 1.00 |
| CELSR2 | Q9HCU4 | OID30593 | LDL | 1780 | 2.42E-102 | 1.59E-92 | 5.3E-13 | 2.46E-03 | 1.00 |
| APOE | P02649 | OID30727 | TC | 2234 | 1.84E-35 | 2.49E-18 | 1.9E-20 | 1.60E-03 | 1.00 |
| APOE | P02649 | OID30727 | nonHDL | 2221 | 1.40E-42 | 1.90E-25 | 1.5E-20 | 1.02E-03 | 1.00 |
| PLTP | P55058 | OID20238 | TG | 2297 | 7.85E-19 | 6.27E-07 | 1.5E-15 | 1.75E-04 | 1.00 |

# **Supplementary Table S5A. Result of Generalised Mendelian randomizsation**

| **Protein** | **UniProtID** | **ProbeID** | **Outcome** | **Threshold (P)** | **Threshold (R^2^)** | **Method** | **n SNP** | **CI** | **P** | **FDR** | **gEgger**  **Intercept** | **P Intercept** | **FDR Intercept** |
| --- | --- | --- | --- | --- | --- | --- | --- | --- | --- | --- | --- | --- | --- |
| ANGPTL3 | Q9Y5C1 | OID20407 | HDL-C | 1.00E-04 | 0.4 | gEgger | 3 | 0.08 (-0.02, 0.19) | 0.13 | 0.18 | 8.3E-04 | 0.96 | 0.96 |
| ANGPTL3 | Q9Y5C1 | OID20407 | HDL-C | 1.00E-04 | 0.4 | gIVW | 3 | 0.09 (0.04, 0.13) | 2.1E-04 | 6.8E-04 | NA | NA | NA |
| ANGPTL3 | Q9Y5C1 | OID20407 | HDL-C | 1.00E-04 | 0.4 | Weighted median | 3 | 0.09 (0.04, 0.13) | 1.9E-04 | 1.9E-04 | NA | NA | NA |
| ANGPTL3 | Q9Y5C1 | OID20407 | LDL-C | 1.00E-04 | 0.4 | gEgger | 3 | 0.13 (0.02, 0.24) | 0.02 | 0.03 | -9.3E-03 | 0.53 | 0.85 |
| ANGPTL3 | Q9Y5C1 | OID20407 | LDL-C | 1.00E-04 | 0.4 | gIVW | 3 | 0.1 (0.05, 0.15) | 1.9E-05 | 6.5E-05 | NA | NA | NA |
| ANGPTL3 | Q9Y5C1 | OID20407 | LDL-C | 1.00E-04 | 0.4 | Weighted median | 3 | 0.1 (0.05, 0.15) | 3.6E-05 | 4.3E-05 | NA | NA | NA |
| ANGPTL3 | Q9Y5C1 | OID20407 | nonHDL-C | 1.00E-04 | 0.4 | gEgger | 3 | 0.19 (0.08, 0.3) | 7.0E-04 | 3.2E-03 | -4.3E-03 | 0.78 | 0.78 |
| ANGPTL3 | Q9Y5C1 | OID20407 | nonHDL-C | 1.00E-04 | 0.4 | gIVW | 3 | 0.18 (0.13, 0.23) | 2.0E-13 | 1.8E-12 | NA | NA | NA |
| ANGPTL3 | Q9Y5C1 | OID20407 | nonHDL-C | 1.00E-04 | 0.4 | Weighted median | 3 | 0.18 (0.12, 0.24) | 3.0E-08 | 6.8E-08 | NA | NA | NA |
| ANGPTL3 | Q9Y5C1 | OID20407 | TC | 1.00E-04 | 0.4 | gEgger | 3 | 0.23 (0.12, 0.33) | 3.6E-05 | 3.6E-04 | -6.4E-03 | 0.66 | 0.89 |
| ANGPTL3 | Q9Y5C1 | OID20407 | TC | 1.00E-04 | 0.4 | gIVW | 3 | 0.21 (0.16, 0.25) | 1.8E-18 | 1.8E-17 | NA | NA | NA |
| ANGPTL3 | Q9Y5C1 | OID20407 | TC | 1.00E-04 | 0.4 | Weighted median | 3 | 0.21 (0.14, 0.27) | 2.9E-09 | 1.1E-08 | NA | NA | NA |
| ANGPTL3 | Q9Y5C1 | OID20407 | TG | 1.00E-04 | 0.4 | gEgger | 3 | 0.26 (0.16, 0.37) | 1.7E-06 | 1.2E-05 | 6.0E-04 | 0.97 | 0.98 |
| ANGPTL3 | Q9Y5C1 | OID20407 | TG | 1.00E-04 | 0.4 | gIVW | 3 | 0.27 (0.22, 0.31) | 1.1E-29 | 1.1E-28 | NA | NA | NA |
| ANGPTL3 | Q9Y5C1 | OID20407 | TG | 1.00E-04 | 0.4 | Weighted median | 3 | 0.26 (0.18, 0.35) | 3.0E-10 | 7.0E-10 | NA | NA | NA |
| ANGPTL4 | Q9BY76 | OID20703 | HDL-C | 1.00E-04 | 0.4 | gIVW | 4 | 0.05 (-0.05, 0.15) | 0.36 | 0.36 | NA | NA | NA |
| APOC1 | P02654 | OID30749 | HDL-C | 1.00E-04 | 0.4 | gIVW | 4 | -0.09 (-0.17, 0) | 0.05 | 0.06 | NA | NA | NA |
| APOC1 | P02654 | OID30749 | nonHDL-C | 1.00E-04 | 0.4 | gEgger | 4 | 0.11 (-0.06, 0.28) | 0.21 | 0.32 | 9.6E-03 | 0.72 | 0.78 |
| APOC1 | P02654 | OID30749 | nonHDL-C | 1.00E-04 | 0.4 | gIVW | 4 | 0.14 (0.1, 0.18) | 2.2E-10 | 9.9E-10 | NA | NA | NA |
| APOC1 | P02654 | OID30749 | nonHDL-C | 1.00E-04 | 0.4 | Weighted median | 4 | 0.14 (0.09, 0.19) | 5.7E-08 | 1.0E-07 | NA | NA | NA |
| APOC1 | P02654 | OID30749 | TC | 1.00E-04 | 0.4 | gEgger | 4 | 0.06 (-0.1, 0.23) | 0.47 | 0.52 | 0.02 | 0.48 | 0.80 |
| APOC1 | P02654 | OID30749 | TC | 1.00E-04 | 0.4 | gIVW | 4 | 0.12 (0.08, 0.16) | 2.4E-08 | 1.2E-07 | NA | NA | NA |
| APOC1 | P02654 | OID30749 | TC | 1.00E-04 | 0.4 | Weighted median | 4 | 0.13 (0.08, 0.17) | 1.2E-07 | 1.9E-07 | NA | NA | NA |
| APOC1 | P02654 | OID30749 | TG | 1.00E-04 | 0.4 | gEgger | 4 | 0.72 (0.11, 1.33) | 0.02 | 0.03 | -0.15 | 0.11 | 0.39 |
| APOC1 | P02654 | OID30749 | TG | 1.00E-04 | 0.4 | gIVW | 4 | 0.24 (0.05, 0.43) | 0.01 | 0.02 | NA | NA | NA |
| APOC1 | P02654 | OID30749 | TG | 1.00E-04 | 0.4 | Weighted median | 4 | 0.25 (0.18, 0.32) | 2.5E-13 | 8.8E-13 | NA | NA | NA |
| APOE | P02649 | OID30727 | HDL-C | 5.00E-06 | 0.1 | gEgger | 3 | 0.22 (0.16, 0.27) | 3.6E-15 | 1.4E-14 | -0.06 | 1.1E-05 | 4.5E-05 |
| APOE | P02649 | OID30727 | HDL-C | 5.00E-06 | 0.1 | gIVW | 3 | 0.11 (0.03, 0.2) | 0.01 | 0.02 | NA | NA | NA |
| APOE | P02649 | OID30727 | HDL-C | 5.00E-06 | 0.1 | Weighted median | 3 | 0.1 (0.07, 0.14) | 1.9E-09 | 3.8E-09 | NA | NA | NA |
| APOE | P02649 | OID30727 | HDL-C | 1.00E-04 | 0.4 | gEgger | 11 | 0.1 (0.02, 0.17) | 0.01 | 0.04 | 1.8E-03 | 0.88 | 0.96 |
| APOE | P02649 | OID30727 | HDL-C | 1.00E-04 | 0.4 | gIVW | 11 | 0.1 (0.05, 0.16) | 4.1E-04 | 1.1E-03 | NA | NA | NA |
| APOE | P02649 | OID30727 | HDL-C | 1.00E-04 | 0.4 | Weighted median | 11 | 0.15 (0.11, 0.18) | 2.7E-18 | 1.4E-17 | NA | NA | NA |
| APOE | P02649 | OID30727 | LDL-C | 5.00E-06 | 0.1 | gIVW | 3 | -0.07 (-0.22, 0.08) | 0.36 | 0.36 | NA | NA | NA |
| APOE | P02649 | OID30727 | LDL-C | 1.00E-04 | 0.4 | gIVW | 11 | -0.12 (-0.25, 0.02) | 0.09 | 0.09 | NA | NA | NA |
| APOE | P02649 | OID30727 | nonHDL-C | 5.00E-06 | 0.1 | gIVW | 3 | -0.12 (-0.31, 0.07) | 0.23 | 0.23 | NA | NA | NA |
| APOE | P02649 | OID30727 | nonHDL-C | 1.00E-04 | 0.4 | gEgger | 11 | -0.19 (-0.34, -0.04) | 0.01 | 0.04 | 0.03 | 0.29 | 0.64 |
| APOE | P02649 | OID30727 | nonHDL-C | 1.00E-04 | 0.4 | gIVW | 11 | -0.14 (-0.26, -0.02) | 0.02 | 0.02 | NA | NA | NA |
| APOE | P02649 | OID30727 | nonHDL-C | 1.00E-04 | 0.4 | Weighted median | 11 | -0.15 (-0.2, -0.1) | 4.5E-10 | 2.0E-09 | NA | NA | NA |
| APOE | P02649 | OID30727 | TC | 5.00E-06 | 0.1 | gIVW | 3 | -0.09 (-0.27, 0.09) | 0.32 | 0.32 | NA | NA | NA |
| APOE | P02649 | OID30727 | TC | 1.00E-04 | 0.4 | gEgger | 11 | -0.16 (-0.3, -0.03) | 0.02 | 0.05 | 0.03 | 0.26 | 0.80 |
| APOE | P02649 | OID30727 | TC | 1.00E-04 | 0.4 | gIVW | 11 | -0.12 (-0.23, 0) | 0.04 | 0.05 | NA | NA | NA |
| APOE | P02649 | OID30727 | TC | 1.00E-04 | 0.4 | Weighted median | 11 | -0.12 (-0.16, -0.08) | 3.4E-09 | 1.1E-08 | NA | NA | NA |
| APOE | P02649 | OID30727 | TG | 5.00E-06 | 0.1 | gIVW | 3 | -0.12 (-0.3, 0.06) | 0.20 | 0.20 | NA | NA | NA |
| APOE | P02649 | OID30727 | TG | 1.00E-04 | 0.4 | gIVW | 11 | -0.06 (-0.19, 0.07) | 0.34 | 0.34 | NA | NA | NA |
| CELSR2 | Q9HCU4 | OID30593 | HDL-C | 5.00E-06 | 0.1 | gIVW | 2 | 0.1 (0.06, 0.15) | 1.8E-06 | 8.9E-06 | NA | NA | NA |
| CELSR2 | Q9HCU4 | OID30593 | HDL-C | 1.00E-04 | 0.4 | gEgger | 6 | 0.11 (0.02, 0.21) | 0.02 | 0.05 | -9.3E-03 | 0.62 | 0.89 |
| CELSR2 | Q9HCU4 | OID30593 | HDL-C | 1.00E-04 | 0.4 | gIVW | 6 | 0.09 (0.05, 0.14) | 3.1E-05 | 2.4E-04 | NA | NA | NA |
| CELSR2 | Q9HCU4 | OID30593 | HDL-C | 1.00E-04 | 0.4 | Weighted median | 6 | 0.1 (0.07, 0.13) | 3.7E-09 | 7.4E-09 | NA | NA | NA |
| CELSR2 | Q9HCU4 | OID30593 | LDL-C | 5.00E-06 | 0.1 | gIVW | 2 | -0.34 (-0.49, -0.19) | 8.0E-06 | 3.0E-05 | NA | NA | NA |
| CELSR2 | Q9HCU4 | OID30593 | LDL-C | 1.00E-04 | 0.4 | gEgger | 6 | -0.38 (-0.65, -0.1) | 6.8E-03 | 0.03 | 0.03 | 0.55 | 0.85 |
| CELSR2 | Q9HCU4 | OID30593 | LDL-C | 1.00E-04 | 0.4 | gIVW | 6 | -0.3 (-0.43, -0.18) | 2.4E-06 | 1.7E-05 | NA | NA | NA |
| CELSR2 | Q9HCU4 | OID30593 | LDL-C | 1.00E-04 | 0.4 | Weighted median | 6 | -0.32 (-0.39, -0.24) | 1.1E-16 | 6.5E-16 | NA | NA | NA |
| CELSR2 | Q9HCU4 | OID30593 | nonHDL-C | 5.00E-06 | 0.1 | gIVW | 2 | -0.32 (-0.45, -0.2) | 3.0E-07 | 1.8E-06 | NA | NA | NA |
| CELSR2 | Q9HCU4 | OID30593 | nonHDL-C | 1.00E-04 | 0.4 | gEgger | 6 | -0.35 (-0.63, -0.06) | 0.02 | 0.04 | 0.03 | 0.63 | 0.78 |
| CELSR2 | Q9HCU4 | OID30593 | nonHDL-C | 1.00E-04 | 0.4 | gIVW | 6 | -0.29 (-0.42, -0.16) | 1.8E-05 | 3.2E-05 | NA | NA | NA |
| CELSR2 | Q9HCU4 | OID30593 | nonHDL-C | 1.00E-04 | 0.4 | Weighted median | 6 | -0.3 (-0.38, -0.22) | 3.7E-14 | 3.3E-13 | NA | NA | NA |
| CELSR2 | Q9HCU4 | OID30593 | TC | 5.00E-06 | 0.1 | gIVW | 2 | -0.29 (-0.39, -0.18) | 1.1E-07 | 8.0E-07 | NA | NA | NA |
| CELSR2 | Q9HCU4 | OID30593 | TC | 1.00E-04 | 0.4 | gEgger | 6 | -0.29 (-0.54, -0.05) | 0.02 | 0.05 | 0.02 | 0.71 | 0.89 |
| CELSR2 | Q9HCU4 | OID30593 | TC | 1.00E-04 | 0.4 | gIVW | 6 | -0.25 (-0.36, -0.14) | 5.3E-06 | 1.3E-05 | NA | NA | NA |
| CELSR2 | Q9HCU4 | OID30593 | TC | 1.00E-04 | 0.4 | Weighted median | 6 | -0.26 (-0.33, -0.2) | 2.7E-14 | 2.7E-13 | NA | NA | NA |
| CGREF1 | Q99674 | OID20152 | TG | 5.00E-06 | 0.1 | gIVW | 2 | -0.07 (-0.1, -0.04) | 2.7E-05 | 5.3E-05 | NA | NA | NA |
| CGREF1 | Q99674 | OID20152 | TG | 1.00E-04 | 0.4 | gEgger | 5 | -0.13 (-0.37, 0.11) | 0.28 | 0.32 | 0.02 | 0.63 | 0.98 |
| CGREF1 | Q99674 | OID20152 | TG | 1.00E-04 | 0.4 | gIVW | 5 | -0.08 (-0.12, -0.03) | 2.9E-03 | 4.9E-03 | NA | NA | NA |
| CGREF1 | Q99674 | OID20152 | TG | 1.00E-04 | 0.4 | Weighted median | 5 | -0.08 (-0.1, -0.05) | 5.1E-08 | 7.1E-08 | NA | NA | NA |
| DPEP2 | Q9H4A9 | OID21305 | HDL-C | 5.00E-06 | 0.1 | gIVW | 2 | -0.05 (-0.07, -0.03) | 7.7E-06 | 2.6E-05 | NA | NA | NA |
| DPEP2 | Q9H4A9 | OID21305 | HDL-C | 1.00E-04 | 0.4 | gEgger | 7 | -0.04 (-0.1, 0.02) | 0.23 | 0.25 | -0.02 | 0.33 | 0.89 |
| DPEP2 | Q9H4A9 | OID21305 | HDL-C | 1.00E-04 | 0.4 | gIVW | 7 | -0.06 (-0.1, -0.01) | 0.02 | 0.03 | NA | NA | NA |
| DPEP2 | Q9H4A9 | OID21305 | HDL-C | 1.00E-04 | 0.4 | Weighted median | 7 | -0.06 (-0.08, -0.04) | 1.9E-06 | 3.2E-06 | NA | NA | NA |
| EPPK1 | P58107 | OID30250 | LDL-C | 5.00E-06 | 0.1 | gIVW | 2 | -0.08 (-0.12, -0.04) | 9.3E-05 | 1.9E-04 | NA | NA | NA |
| EPPK1 | P58107 | OID30250 | LDL-C | 1.00E-04 | 0.4 | gEgger | 6 | -0.02 (-0.17, 0.14) | 0.81 | 0.81 | -0.01 | 0.57 | 0.85 |
| EPPK1 | P58107 | OID30250 | LDL-C | 1.00E-04 | 0.4 | gIVW | 6 | -0.06 (-0.11, -0.01) | 0.01 | 0.01 | NA | NA | NA |
| EPPK1 | P58107 | OID30250 | LDL-C | 1.00E-04 | 0.4 | Weighted median | 6 | -0.05 (-0.09, -0.02) | 3.0E-03 | 3.0E-03 | NA | NA | NA |
| EPPK1 | P58107 | OID30250 | nonHDL-C | 5.00E-06 | 0.1 | gIVW | 2 | -0.08 (-0.13, -0.04) | 1.5E-04 | 4.5E-04 | NA | NA | NA |
| EPPK1 | P58107 | OID30250 | nonHDL-C | 1.00E-04 | 0.4 | gEgger | 6 | 0 (-0.13, 0.13) | 0.97 | 0.97 | -0.02 | 0.27 | 0.64 |
| EPPK1 | P58107 | OID30250 | nonHDL-C | 1.00E-04 | 0.4 | gIVW | 6 | -0.07 (-0.11, -0.03) | 1.9E-03 | 2.2E-03 | NA | NA | NA |
| EPPK1 | P58107 | OID30250 | nonHDL-C | 1.00E-04 | 0.4 | Weighted median | 6 | -0.08 (-0.12, -0.04) | 1.7E-05 | 1.9E-05 | NA | NA | NA |
| EPPK1 | P58107 | OID30250 | TC | 5.00E-06 | 0.1 | gIVW | 2 | -0.07 (-0.14, 0) | 0.05 | 0.08 | NA | NA | NA |
| EPPK1 | P58107 | OID30250 | TC | 1.00E-04 | 0.4 | gEgger | 6 | 0 (-0.16, 0.17) | 0.95 | 0.95 | -0.02 | 0.46 | 0.80 |
| EPPK1 | P58107 | OID30250 | TC | 1.00E-04 | 0.4 | gIVW | 6 | -0.05 (-0.11, 0) | 0.05 | 0.05 | NA | NA | NA |
| EPPK1 | P58107 | OID30250 | TC | 1.00E-04 | 0.4 | Weighted median | 6 | -0.06 (-0.09, -0.02) | 1.9E-03 | 1.9E-03 | NA | NA | NA |
| GAS6 | Q14393 | OID20318 | nonHDL-C | 1.00E-04 | 0.4 | gEgger | 9 | -0.02 (-0.1, 0.06) | 0.66 | 0.74 | -0.02 | 0.06 | 0.54 |
| GAS6 | Q14393 | OID20318 | nonHDL-C | 1.00E-04 | 0.4 | gIVW | 9 | -0.09 (-0.12, -0.05) | 1.5E-06 | 4.5E-06 | NA | NA | NA |
| GAS6 | Q14393 | OID20318 | nonHDL-C | 1.00E-04 | 0.4 | Weighted median | 9 | -0.09 (-0.12, -0.06) | 4.9E-09 | 1.5E-08 | NA | NA | NA |
| GAS6 | Q14393 | OID20318 | TC | 1.00E-04 | 0.4 | gEgger | 9 | -0.03 (-0.11, 0.05) | 0.43 | 0.52 | -0.01 | 0.23 | 0.80 |
| GAS6 | Q14393 | OID20318 | TC | 1.00E-04 | 0.4 | gIVW | 9 | -0.07 (-0.11, -0.04) | 2.9E-05 | 5.9E-05 | NA | NA | NA |
| GAS6 | Q14393 | OID20318 | TC | 1.00E-04 | 0.4 | Weighted median | 9 | -0.08 (-0.11, -0.05) | 1.5E-08 | 3.8E-08 | NA | NA | NA |
| GAS6 | Q14393 | OID20318 | TG | 1.00E-04 | 0.4 | gEgger | 9 | -0.03 (-0.1, 0.05) | 0.50 | 0.50 | -0.02 | 0.11 | 0.39 |
| GAS6 | Q14393 | OID20318 | TG | 1.00E-04 | 0.4 | gIVW | 9 | -0.08 (-0.12, -0.05) | 2.2E-06 | 7.5E-06 | NA | NA | NA |
| GAS6 | Q14393 | OID20318 | TG | 1.00E-04 | 0.4 | Weighted median | 9 | -0.08 (-0.11, -0.05) | 4.2E-08 | 7.1E-08 | NA | NA | NA |
| GIPC3 | Q8TF64 | OID31507 | HDL-C | 1.00E-04 | 0.4 | gIVW | 2 | -0.09 (-0.16, -0.02) | 7.8E-03 | 0.02 | NA | NA | NA |
| GSTA1 | P08263 | OID20166 | TG | 1.00E-04 | 0.4 | gEgger | 5 | -0.12 (-0.21, -0.03) | 0.01 | 0.02 | 9.5E-03 | 0.49 | 0.98 |
| GSTA1 | P08263 | OID20166 | TG | 1.00E-04 | 0.4 | gIVW | 5 | -0.09 (-0.13, -0.05) | 3.6E-05 | 7.2E-05 | NA | NA | NA |
| GSTA1 | P08263 | OID20166 | TG | 1.00E-04 | 0.4 | Weighted median | 5 | -0.08 (-0.12, -0.05) | 3.1E-06 | 3.1E-06 | NA | NA | NA |
| GSTA3 | Q16772 | OID21242 | TG | 1.00E-04 | 0.4 | gEgger | 6 | -0.07 (-0.13, -0.02) | 0.01 | 0.02 | -2.2E-03 | 0.80 | 0.98 |
| GSTA3 | Q16772 | OID21242 | TG | 1.00E-04 | 0.4 | gIVW | 6 | -0.08 (-0.12, -0.04) | 1.7E-05 | 4.2E-05 | NA | NA | NA |
| GSTA3 | Q16772 | OID21242 | TG | 1.00E-04 | 0.4 | Weighted median | 6 | -0.08 (-0.11, -0.05) | 6.8E-07 | 7.9E-07 | NA | NA | NA |
| GSTM4 | Q03013 | OID30164 | HDL-C | 5.00E-06 | 0.1 | gIVW | 3 | 0.01 (-0.12, 0.15) | 0.84 | 0.84 | NA | NA | NA |
| GSTM4 | Q03013 | OID30164 | HDL-C | 1.00E-04 | 0.4 | gIVW | 12 | -0.08 (-0.2, 0.03) | 0.15 | 0.17 | NA | NA | NA |
| HGFAC | Q04756 | OID30718 | LDL-C | 5.00E-06 | 0.1 | gEgger | 4 | -0.03 (-0.27, 0.22) | 0.82 | 0.82 | -7.6E-03 | 0.88 | 0.88 |
| HGFAC | Q04756 | OID30718 | LDL-C | 5.00E-06 | 0.1 | gIVW | 4 | -0.05 (-0.08, -0.01) | 5.0E-03 | 7.5E-03 | NA | NA | NA |
| HGFAC | Q04756 | OID30718 | LDL-C | 5.00E-06 | 0.1 | Weighted median | 4 | -0.04 (-0.07, -0.01) | 0.02 | 0.02 | NA | NA | NA |
| HGFAC | Q04756 | OID30718 | LDL-C | 1.00E-04 | 0.4 | gEgger | 12 | -0.04 (-0.08, -0.01) | 0.01 | 0.03 | 3.9E-03 | 0.49 | 0.85 |
| HGFAC | Q04756 | OID30718 | LDL-C | 1.00E-04 | 0.4 | gIVW | 12 | -0.04 (-0.06, -0.01) | 4.7E-03 | 8.3E-03 | NA | NA | NA |
| HGFAC | Q04756 | OID30718 | LDL-C | 1.00E-04 | 0.4 | Weighted median | 12 | -0.05 (-0.08, -0.03) | 1.8E-05 | 2.7E-05 | NA | NA | NA |
| HGFAC | Q04756 | OID30718 | nonHDL-C | 5.00E-06 | 0.1 | gEgger | 4 | 0.08 (-0.1, 0.26) | 0.38 | 0.38 | -0.06 | 0.12 | 0.12 |
| HGFAC | Q04756 | OID30718 | nonHDL-C | 5.00E-06 | 0.1 | gIVW | 4 | -0.06 (-0.1, -0.03) | 4.8E-04 | 9.7E-04 | NA | NA | NA |
| HGFAC | Q04756 | OID30718 | nonHDL-C | 5.00E-06 | 0.1 | Weighted median | 4 | -0.07 (-0.1, -0.04) | 3.8E-05 | 3.8E-05 | NA | NA | NA |
| HGFAC | Q04756 | OID30718 | nonHDL-C | 1.00E-04 | 0.4 | gEgger | 12 | -0.07 (-0.1, -0.03) | 1.0E-04 | 9.0E-04 | 5.5E-03 | 0.36 | 0.64 |
| HGFAC | Q04756 | OID30718 | nonHDL-C | 1.00E-04 | 0.4 | gIVW | 12 | -0.06 (-0.08, -0.03) | 1.3E-05 | 3.0E-05 | NA | NA | NA |
| HGFAC | Q04756 | OID30718 | nonHDL-C | 1.00E-04 | 0.4 | Weighted median | 12 | -0.07 (-0.09, -0.04) | 3.5E-07 | 5.3E-07 | NA | NA | NA |
| HGFAC | Q04756 | OID30718 | TC | 5.00E-06 | 0.1 | gEgger | 4 | 0.05 (-0.12, 0.22) | 0.57 | 0.57 | -0.05 | 0.20 | 0.20 |
| HGFAC | Q04756 | OID30718 | TC | 5.00E-06 | 0.1 | gIVW | 4 | -0.06 (-0.09, -0.03) | 1.0E-04 | 3.5E-04 | NA | NA | NA |
| HGFAC | Q04756 | OID30718 | TC | 5.00E-06 | 0.1 | Weighted median | 4 | -0.07 (-0.1, -0.03) | 5.9E-05 | 1.2E-04 | NA | NA | NA |
| HGFAC | Q04756 | OID30718 | TC | 1.00E-04 | 0.4 | gEgger | 12 | -0.06 (-0.09, -0.03) | 3.3E-04 | 1.7E-03 | 4.4E-04 | 0.94 | 0.94 |
| HGFAC | Q04756 | OID30718 | TC | 1.00E-04 | 0.4 | gIVW | 12 | -0.06 (-0.08, -0.03) | 2.7E-06 | 8.9E-06 | NA | NA | NA |
| HGFAC | Q04756 | OID30718 | TC | 1.00E-04 | 0.4 | Weighted median | 12 | -0.07 (-0.09, -0.04) | 7.9E-08 | 1.6E-07 | NA | NA | NA |
| KHK | P50053 | OID30241 | TG | 1.00E-04 | 0.4 | gIVW | 6 | 0.06 (0, 0.13) | 0.05 | 0.06 | NA | NA | NA |
| LILRA3 | Q8N6C8 | OID30376 | HDL-C | 5.00E-06 | 0.1 | gEgger | 8 | -0.03 (-0.06, 0) | 0.03 | 0.05 | -0.02 | 0.03 | 0.07 |
| LILRA3 | Q8N6C8 | OID30376 | HDL-C | 5.00E-06 | 0.1 | gIVW | 8 | -0.05 (-0.08, -0.03) | 1.5E-07 | 1.5E-06 | NA | NA | NA |
| LILRA3 | Q8N6C8 | OID30376 | HDL-C | 5.00E-06 | 0.1 | Weighted median | 8 | -0.06 (-0.07, -0.04) | 1.8E-14 | 7.0E-14 | NA | NA | NA |
| LILRA3 | Q8N6C8 | OID30376 | HDL-C | 1.00E-04 | 0.4 | gEgger | 30 | -0.06 (-0.07, -0.04) | 1.2E-13 | 1.2E-12 | -1.2E-03 | 0.56 | 0.89 |
| LILRA3 | Q8N6C8 | OID30376 | HDL-C | 1.00E-04 | 0.4 | gIVW | 30 | -0.06 (-0.07, -0.04) | 1.0E-16 | 1.6E-15 | NA | NA | NA |
| LILRA3 | Q8N6C8 | OID30376 | HDL-C | 1.00E-04 | 0.4 | Weighted median | 30 | -0.06 (-0.07, -0.05) | 7.2E-43 | 7.2E-42 | NA | NA | NA |
| LILRA5 | A6NI73 | OID20209 | HDL-C | 1.00E-04 | 0.4 | gIVW | 4 | -0.07 (-0.21, 0.06) | 0.28 | 0.30 | NA | NA | NA |
| LILRB2 | Q8N423 | OID20301 | HDL-C | 5.00E-06 | 0.1 | gIVW | 6 | -0.04 (-0.1, 0.02) | 0.19 | 0.24 | NA | NA | NA |
| LILRB2 | Q8N423 | OID20301 | HDL-C | 1.00E-04 | 0.4 | gEgger | 23 | -0.04 (-0.08, 0) | 0.03 | 0.06 | 7.7E-04 | 0.89 | 0.96 |
| LILRB2 | Q8N423 | OID20301 | HDL-C | 1.00E-04 | 0.4 | gIVW | 23 | -0.04 (-0.08, -0.01) | 0.02 | 0.03 | NA | NA | NA |
| LILRB2 | Q8N423 | OID20301 | HDL-C | 1.00E-04 | 0.4 | Weighted median | 23 | -0.06 (-0.07, -0.04) | 1.9E-10 | 4.8E-10 | NA | NA | NA |
| LILRB5 | O75023 | OID20324 | HDL-C | 5.00E-06 | 0.1 | gEgger | 12 | 0.01 (-0.04, 0.06) | 0.70 | 0.70 | 0.02 | 0.07 | 0.09 |
| LILRB5 | O75023 | OID20324 | HDL-C | 5.00E-06 | 0.1 | gIVW | 12 | 0.04 (0, 0.08) | 0.03 | 0.05 | NA | NA | NA |
| LILRB5 | O75023 | OID20324 | HDL-C | 5.00E-06 | 0.1 | Weighted median | 12 | 0.04 (0.02, 0.06) | 4.2E-07 | 5.5E-07 | NA | NA | NA |
| LILRB5 | O75023 | OID20324 | HDL-C | 1.00E-04 | 0.4 | gEgger | 37 | 0.03 (0.01, 0.06) | 4.5E-03 | 0.02 | 5.0E-03 | 0.14 | 0.89 |
| LILRB5 | O75023 | OID20324 | HDL-C | 1.00E-04 | 0.4 | gIVW | 37 | 0.04 (0.02, 0.06) | 1.9E-04 | 6.8E-04 | NA | NA | NA |
| LILRB5 | O75023 | OID20324 | HDL-C | 1.00E-04 | 0.4 | Weighted median | 37 | 0.04 (0.03, 0.05) | 7.8E-14 | 2.6E-13 | NA | NA | NA |
| LPA | P08519 | OID30747 | LDL-C | 5.00E-06 | 0.1 | gEgger | 3 | 0.16 (0.04, 0.28) | 0.01 | 0.02 | -0.02 | 0.24 | 0.49 |
| LPA | P08519 | OID30747 | LDL-C | 5.00E-06 | 0.1 | gIVW | 3 | 0.09 (0.05, 0.13) | 9.9E-06 | 3.0E-05 | NA | NA | NA |
| LPA | P08519 | OID30747 | LDL-C | 5.00E-06 | 0.1 | Weighted median | 3 | 0.08 (0.04, 0.13) | 9.5E-05 | 1.9E-04 | NA | NA | NA |
| LPA | P08519 | OID30747 | LDL-C | 1.00E-04 | 0.4 | gEgger | 9 | 0.07 (-0.04, 0.18) | 0.21 | 0.28 | -1.9E-03 | 0.91 | 0.91 |
| LPA | P08519 | OID30747 | LDL-C | 1.00E-04 | 0.4 | gIVW | 9 | 0.07 (0.02, 0.12) | 9.4E-03 | 0.01 | NA | NA | NA |
| LPA | P08519 | OID30747 | LDL-C | 1.00E-04 | 0.4 | Weighted median | 9 | 0.1 (0.07, 0.13) | 1.4E-09 | 4.2E-09 | NA | NA | NA |
| LPA | P08519 | OID30747 | TC | 5.00E-06 | 0.1 | gEgger | 3 | 0.16 (0.04, 0.28) | 9.4E-03 | 0.02 | -0.03 | 0.11 | 0.20 |
| LPA | P08519 | OID30747 | TC | 5.00E-06 | 0.1 | gIVW | 3 | 0.07 (0.02, 0.11) | 9.0E-03 | 0.02 | NA | NA | NA |
| LPA | P08519 | OID30747 | TC | 5.00E-06 | 0.1 | Weighted median | 3 | 0.05 (0.01, 0.09) | 9.4E-03 | 9.4E-03 | NA | NA | NA |
| LPA | P08519 | OID30747 | TC | 1.00E-04 | 0.4 | gEgger | 9 | 0.08 (-0.01, 0.17) | 0.07 | 0.15 | -0.01 | 0.37 | 0.80 |
| LPA | P08519 | OID30747 | TC | 1.00E-04 | 0.4 | gIVW | 9 | 0.05 (0, 0.09) | 0.03 | 0.04 | NA | NA | NA |
| LPA | P08519 | OID30747 | TC | 1.00E-04 | 0.4 | Weighted median | 9 | 0.07 (0.04, 0.1) | 3.6E-05 | 4.5E-05 | NA | NA | NA |
| PCSK7 | Q16549 | OID31410 | TG | 5.00E-06 | 0.1 | gEgger | 4 | 0.16 (-0.09, 0.4) | 0.21 | 0.21 | -0.12 | 0.01 | 0.01 |
| PCSK7 | Q16549 | OID31410 | TG | 5.00E-06 | 0.1 | gIVW | 4 | -0.14 (-0.26, -0.03) | 0.01 | 0.02 | NA | NA | NA |
| PCSK7 | Q16549 | OID31410 | TG | 5.00E-06 | 0.1 | Weighted median | 4 | -0.13 (-0.18, -0.09) | 8.1E-09 | 8.1E-09 | NA | NA | NA |
| PCSK7 | Q16549 | OID31410 | TG | 1.00E-04 | 0.4 | gIVW | 16 | -0.1 (-0.21, 0.01) | 0.09 | 0.10 | NA | NA | NA |
| PCSK9 | Q8NBP7 | OID20235 | LDL-C | 5.00E-06 | 0.1 | gIVW | 2 | 0.12 (0.01, 0.23) | 0.03 | 0.04 | NA | NA | NA |
| PCSK9 | Q8NBP7 | OID20235 | LDL-C | 1.00E-04 | 0.4 | gEgger | 6 | 0.1 (-0.06, 0.26) | 0.23 | 0.28 | 5.3E-03 | 0.82 | 0.91 |
| PCSK9 | Q8NBP7 | OID20235 | LDL-C | 1.00E-04 | 0.4 | gIVW | 6 | 0.12 (0.06, 0.17) | 4.6E-05 | 1.1E-04 | NA | NA | NA |
| PCSK9 | Q8NBP7 | OID20235 | LDL-C | 1.00E-04 | 0.4 | Weighted median | 6 | 0.13 (0.09, 0.17) | 2.5E-09 | 5.0E-09 | NA | NA | NA |
| PCSK9 | Q8NBP7 | OID20235 | nonHDL-C | 5.00E-06 | 0.1 | gIVW | 2 | 0.1 (0.01, 0.19) | 0.04 | 0.04 | NA | NA | NA |
| PCSK9 | Q8NBP7 | OID20235 | nonHDL-C | 1.00E-04 | 0.4 | gEgger | 6 | 0.12 (-0.05, 0.29) | 0.16 | 0.29 | -8.2E-03 | 0.74 | 0.78 |
| PCSK9 | Q8NBP7 | OID20235 | nonHDL-C | 1.00E-04 | 0.4 | gIVW | 6 | 0.09 (0.04, 0.15) | 1.5E-03 | 1.9E-03 | NA | NA | NA |
| PCSK9 | Q8NBP7 | OID20235 | nonHDL-C | 1.00E-04 | 0.4 | Weighted median | 6 | 0.1 (0.06, 0.14) | 1.4E-06 | 1.9E-06 | NA | NA | NA |
| PCSK9 | Q8NBP7 | OID20235 | TC | 5.00E-06 | 0.1 | gIVW | 2 | 0.09 (-0.01, 0.18) | 0.07 | 0.10 | NA | NA | NA |
| PCSK9 | Q8NBP7 | OID20235 | TC | 1.00E-04 | 0.4 | gEgger | 6 | 0.1 (-0.07, 0.27) | 0.25 | 0.36 | -4.9E-03 | 0.84 | 0.93 |
| PCSK9 | Q8NBP7 | OID20235 | TC | 1.00E-04 | 0.4 | gIVW | 6 | 0.08 (0.02, 0.14) | 5.3E-03 | 8.8E-03 | NA | NA | NA |
| PCSK9 | Q8NBP7 | OID20235 | TC | 1.00E-04 | 0.4 | Weighted median | 6 | 0.09 (0.05, 0.13) | 3.5E-06 | 4.9E-06 | NA | NA | NA |
| PECR | Q9BY49 | OID30228 | HDL-C | 5.00E-06 | 0.1 | gEgger | 3 | -0.04 (-0.13, 0.05) | 0.34 | 0.46 | -4.5E-03 | 0.81 | 0.81 |
| PECR | Q9BY49 | OID30228 | HDL-C | 5.00E-06 | 0.1 | gIVW | 3 | -0.05 (-0.08, -0.02) | 4.1E-04 | 1.0E-03 | NA | NA | NA |
| PECR | Q9BY49 | OID30228 | HDL-C | 5.00E-06 | 0.1 | Weighted median | 3 | -0.05 (-0.08, -0.02) | 4.2E-04 | 4.2E-04 | NA | NA | NA |
| PECR | Q9BY49 | OID30228 | HDL-C | 1.00E-04 | 0.4 | gEgger | 7 | -0.06 (-0.12, 0) | 0.06 | 0.10 | 9.8E-03 | 0.43 | 0.89 |
| PECR | Q9BY49 | OID30228 | HDL-C | 1.00E-04 | 0.4 | gIVW | 7 | -0.04 (-0.07, 0) | 0.03 | 0.04 | NA | NA | NA |
| PECR | Q9BY49 | OID30228 | HDL-C | 1.00E-04 | 0.4 | Weighted median | 7 | -0.05 (-0.07, -0.03) | 8.4E-06 | 1.2E-05 | NA | NA | NA |
| PLA2G15 | Q8NCC3 | OID21473 | HDL-C | 5.00E-06 | 0.1 | gIVW | 2 | -0.16 (-0.3, -0.02) | 0.02 | 0.04 | NA | NA | NA |
| PLA2G15 | Q8NCC3 | OID21473 | HDL-C | 1.00E-04 | 0.4 | gEgger | 4 | -0.25 (-0.59, 0.09) | 0.15 | 0.19 | 0.03 | 0.60 | 0.89 |
| PLA2G15 | Q8NCC3 | OID21473 | HDL-C | 1.00E-04 | 0.4 | gIVW | 4 | -0.16 (-0.24, -0.08) | 8.0E-05 | 4.3E-04 | NA | NA | NA |
| PLA2G15 | Q8NCC3 | OID21473 | HDL-C | 1.00E-04 | 0.4 | Weighted median | 4 | -0.13 (-0.2, -0.07) | 3.5E-05 | 4.4E-05 | NA | NA | NA |
| PLTP | P55058 | OID20238 | TG | 5.00E-06 | 0.1 | gIVW | 2 | -0.1 (-0.14, -0.07) | 1.3E-08 | 5.4E-08 | NA | NA | NA |
| PLTP | P55058 | OID20238 | TG | 1.00E-04 | 0.4 | gEgger | 14 | -0.09 (-0.13, -0.05) | 3.4E-06 | 1.2E-05 | -1.1E-04 | 0.98 | 0.98 |
| PLTP | P55058 | OID20238 | TG | 1.00E-04 | 0.4 | gIVW | 14 | -0.09 (-0.13, -0.06) | 2.4E-09 | 1.2E-08 | NA | NA | NA |
| PLTP | P55058 | OID20238 | TG | 1.00E-04 | 0.4 | Weighted median | 14 | -0.11 (-0.13, -0.08) | 7.3E-19 | 5.1E-18 | NA | NA | NA |
| THOP1 | P52888 | OID20194 | HDL-C | 1.00E-04 | 0.4 | gEgger | 7 | 0 (-0.1, 0.09) | 0.98 | 0.98 | 0.02 | 0.21 | 0.89 |
| THOP1 | P52888 | OID20194 | HDL-C | 1.00E-04 | 0.4 | gIVW | 7 | 0.05 (0.02, 0.09) | 6.2E-03 | 0.01 | NA | NA | NA |
| THOP1 | P52888 | OID20194 | HDL-C | 1.00E-04 | 0.4 | Weighted median | 7 | 0.08 (0.04, 0.12) | 7.9E-05 | 8.7E-05 | NA | NA | NA |
| TLR3 | O15455 | OID20612 | HDL-C | 5.00E-06 | 0.1 | gIVW | 10 | -0.01 (-0.03, 0.01) | 0.33 | 0.37 | NA | NA | NA |
| TLR3 | O15455 | OID20612 | HDL-C | 1.00E-04 | 0.4 | gIVW | 33 | 0.02 (0, 0.03) | 0.03 | 0.04 | NA | NA | NA |
| TNFSF13 | O75888 | OID20733 | nonHDL-C | 5.00E-06 | 0.1 | gIVW | 2 | -0.08 (-0.14, -0.01) | 0.02 | 0.03 | NA | NA | NA |
| TNFSF13 | O75888 | OID20733 | nonHDL-C | 1.00E-04 | 0.4 | gEgger | 3 | 0.04 (-0.12, 0.2) | 0.59 | 0.74 | -0.04 | 0.13 | 0.58 |
| TNFSF13 | O75888 | OID20733 | nonHDL-C | 1.00E-04 | 0.4 | gIVW | 3 | -0.08 (-0.12, -0.03) | 4.9E-04 | 7.3E-04 | NA | NA | NA |
| TNFSF13 | O75888 | OID20733 | nonHDL-C | 1.00E-04 | 0.4 | Weighted median | 3 | -0.1 (-0.14, -0.05) | 5.2E-05 | 5.2E-05 | NA | NA | NA |
| TNFSF13 | O75888 | OID20733 | TC | 5.00E-06 | 0.1 | gIVW | 2 | -0.06 (-0.14, 0.03) | 0.17 | 0.20 | NA | NA | NA |
| TNFSF13 | O75888 | OID20733 | TC | 1.00E-04 | 0.4 | gEgger | 3 | 0.09 (-0.06, 0.25) | 0.23 | 0.36 | -0.05 | 0.04 | 0.40 |
| TNFSF13 | O75888 | OID20733 | TC | 1.00E-04 | 0.4 | gIVW | 3 | -0.06 (-0.12, 0) | 0.04 | 0.05 | NA | NA | NA |
| TNFSF13 | O75888 | OID20733 | TC | 1.00E-04 | 0.4 | Weighted median | 3 | -0.08 (-0.13, -0.03) | 1.0E-03 | 1.1E-03 | NA | NA | NA |

# **Supplementary Table S5B. Result of GSMR**

| Protein | Protein.UniProtID | Protein.ProbeID | Outcome | Threshold (P) | Threshold (R^2^) | Method | SNPs | Odds ratio (95% Confidence interval) | P |
| --- | --- | --- | --- | --- | --- | --- | --- | --- | --- |
| ANGPTL3 | Q9Y5C1 | OID20407 | HDL | 1.00E-04 | 0.4 | GSMR | 3 | 0.09 (0.03, 0.14) | 1.4E-03 |
| ANGPTL3 | Q9Y5C1 | OID20407 | LDL | 1.00E-04 | 0.4 | GSMR | 3 | 0.1 (0.04, 0.15) | 4.5E-04 |
| ANGPTL3 | Q9Y5C1 | OID20407 | nonHDL | 1.00E-04 | 0.4 | GSMR | 3 | 0.18 (0.10, 0.25) | 1.7E-06 |
| ANGPTL3 | Q9Y5C1 | OID20407 | TC | 1.00E-04 | 0.4 | GSMR | 3 | 0.2 (0.13, 0.28) | 2.8E-07 |
| ANGPTL3 | Q9Y5C1 | OID20407 | TG | 1.00E-04 | 0.4 | GSMR | 3 | 0.27 (0.17, 0.36) | 3.0E-08 |
| ANGPTL4 | Q9BY76 | OID20703 | HDL | 1.00E-04 | 0.4 | GSMR | 4 | 0.03 (-0.01, 0.08) | 0.15 |
| APOC1 | P02654 | OID30749 | HDL | 1.00E-04 | 0.4 | GSMR | 4 | -0.07 (-0.12, -0.03) | 2.0E-03 |
| APOC1 | P02654 | OID30749 | nonHDL | 1.00E-04 | 0.4 | GSMR | 4 | 0.14 (0.08, 0.19) | 5.8E-07 |
| APOC1 | P02654 | OID30749 | TC | 1.00E-04 | 0.4 | GSMR | 4 | 0.12 (0.07, 0.17) | 4.4E-06 |
| APOC1 | P02654 | OID30749 | TG | 1.00E-04 | 0.4 | GSMR | 4 | 0.13 (0.07, 0.19) | 2.6E-05 |
| APOE | P02649 | OID30727 | HDL | 5.00E-06 | 0.1 | GSMR | 3 | 0.1 (0.06, 0.13) | 7.5E-09 |
| APOE | P02649 | OID30727 | HDL | 1.00E-04 | 0.4 | GSMR | 11 | 0.08 (0.05, 0.11) | 6.5E-08 |
| APOE | P02649 | OID30727 | LDL | 5.00E-06 | 0.1 | GSMR | 3 | -0.06 (-0.09, -0.03) | 1.8E-04 |
| APOE | P02649 | OID30727 | LDL | 1.00E-04 | 0.4 | GSMR | 11 | -0.05 (-0.08, -0.02) | 4.0E-04 |
| APOE | P02649 | OID30727 | nonHDL | 5.00E-06 | 0.1 | GSMR | 3 | -0.08 (-0.12, -0.04) | 1.2E-05 |
| APOE | P02649 | OID30727 | nonHDL | 1.00E-04 | 0.4 | GSMR | 11 | -0.07 (-0.10, -0.03) | 1.1E-04 |
| APOE | P02649 | OID30727 | TC | 5.00E-06 | 0.1 | GSMR | 3 | -0.07 (-0.10, -0.04) | 3.8E-05 |
| APOE | P02649 | OID30727 | TC | 1.00E-04 | 0.4 | GSMR | 11 | -0.05 (-0.09, -0.02) | 4.8E-04 |
| APOE | P02649 | OID30727 | TG | 5.00E-06 | 0.1 | GSMR | 3 | -0.08 (-0.11, -0.04) | 1.4E-05 |
| APOE | P02649 | OID30727 | TG | 1.00E-04 | 0.4 | GSMR | 11 | -0.06 (-0.10, -0.03) | 1.7E-04 |
| CELSR2 | Q9HCU4 | OID30593 | HDL | 1.00E-04 | 0.4 | GSMR | 6 | 0.08 (0.05, 0.12) | 8.8E-06 |
| CELSR2 | Q9HCU4 | OID30593 | LDL | 1.00E-04 | 0.4 | GSMR | 6 | -0.23 (-0.29, -0.16) | 6.8E-13 |
| CELSR2 | Q9HCU4 | OID30593 | nonHDL | 1.00E-04 | 0.4 | GSMR | 6 | -0.2 (-0.26, -0.14) | 2.1E-11 |
| CELSR2 | Q9HCU4 | OID30593 | TC | 1.00E-04 | 0.4 | GSMR | 6 | -0.19 (-0.25, -0.14) | 6.0E-12 |
| CGREF1 | Q99674 | OID20152 | TG | 1.00E-04 | 0.4 | GSMR | 5 | -0.07 (-0.10, -0.04) | 6.7E-05 |
| DPEP2 | Q9H4A9 | OID21305 | HDL | 1.00E-04 | 0.4 | GSMR | 7 | -0.05 (-0.07, -0.03) | 3.7E-06 |
| EPPK1 | P58107 | OID30250 | LDL | 1.00E-04 | 0.4 | GSMR | 6 | -0.05 (-0.09, -0.02) | 3.8E-03 |
| EPPK1 | P58107 | OID30250 | nonHDL | 1.00E-04 | 0.4 | GSMR | 6 | -0.06 (-0.10, -0.02) | 1.3E-03 |
| EPPK1 | P58107 | OID30250 | TC | 1.00E-04 | 0.4 | GSMR | 6 | -0.05 (-0.08, -0.01) | 0.01 |
| GAS6 | Q14393 | OID20318 | nonHDL | 1.00E-04 | 0.4 | GSMR | 9 | -0.08 (-0.12, -0.04) | 9.6E-05 |
| GAS6 | Q14393 | OID20318 | TC | 1.00E-04 | 0.4 | GSMR | 9 | -0.07 (-0.11, -0.03) | 4.5E-04 |
| GAS6 | Q14393 | OID20318 | TG | 1.00E-04 | 0.4 | GSMR | 9 | -0.08 (-0.12, -0.04) | 7.6E-05 |
| GSTA1 | P08263 | OID20166 | TG | 1.00E-04 | 0.4 | GSMR | 5 | -0.08 (-0.13, -0.04) | 4.4E-04 |
| GSTA3 | Q16772 | OID21242 | TG | 1.00E-04 | 0.4 | GSMR | 6 | -0.08 (-0.12, -0.04) | 2.1E-04 |
| GSTM4 | Q03013 | OID30164 | HDL | 1.00E-04 | 0.4 | GSMR | 12 | -0.05 (-0.09, -0.02) | 1.7E-03 |
| GSTM4 | Q03013 | OID30164 | HDL | 5.00E-06 | 0.1 | GSMR | 3 | 0.01 (-0.04, 0.06) | 0.66 |
| HGFAC | Q04756 | OID30718 | LDL | 5.00E-06 | 0.1 | GSMR | 4 | -0.05 (-0.07, -0.02) | 3.0E-03 |
| HGFAC | Q04756 | OID30718 | LDL | 1.00E-04 | 0.4 | GSMR | 12 | -0.03 (-0.06, -0.01) | 8.4E-03 |
| HGFAC | Q04756 | OID30718 | nonHDL | 1.00E-04 | 0.4 | GSMR | 12 | -0.05 (-0.08, -0.03) | 1.4E-04 |
| HGFAC | Q04756 | OID30718 | nonHDL | 5.00E-06 | 0.1 | GSMR | 4 | -0.06 (-0.09, -0.03) | 2.9E-04 |
| HGFAC | Q04756 | OID30718 | TC | 1.00E-04 | 0.4 | GSMR | 12 | -0.06 (-0.08, -0.03) | 2.9E-05 |
| HGFAC | Q04756 | OID30718 | TC | 5.00E-06 | 0.1 | GSMR | 4 | -0.06 (-0.09, -0.03) | 1.3E-04 |
| KHK | P50053 | OID30241 | TG | 1.00E-04 | 0.4 | GSMR | 6 | 0.06 (0.02, 0.09) | 7.1E-04 |
| LILRA3 | Q8N6C8 | OID30376 | HDL | 1.00E-04 | 0.4 | GSMR | 30 | -0.05 (-0.07, -0.04) | 6.0E-18 |
| LILRA3 | Q8N6C8 | OID30376 | HDL | 5.00E-06 | 0.1 | GSMR | 8 | -0.05 (-0.07, -0.04) | 3.1E-13 |
| LILRA5 | A6NI73 | OID20209 | HDL | 1.00E-04 | 0.4 | GSMR | 4 | -0.05 (-0.10, -0.00) | 0.03 |
| LILRB2 | Q8N423 | OID20301 | HDL | 1.00E-04 | 0.4 | GSMR | 23 | -0.04 (-0.05, -0.02) | 8.4E-06 |
| LILRB2 | Q8N423 | OID20301 | HDL | 5.00E-06 | 0.1 | GSMR | 6 | -0.04 (-0.05, -0.02) | 3.4E-05 |
| LILRB5 | O75023 | OID20324 | HDL | 1.00E-04 | 0.4 | GSMR | 37 | 0.03 (0.02, 0.05) | 1.0E-06 |
| LILRB5 | O75023 | OID20324 | HDL | 5.00E-06 | 0.1 | GSMR | 12 | 0.04 (0.02, 0.05) | 3.4E-06 |
| LPA | P08519 | OID30747 | LDL | 5.00E-06 | 0.1 | GSMR | 3 | 0.09 (0.04, 0.13) | 1.7E-04 |
| LPA | P08519 | OID30747 | LDL | 1.00E-04 | 0.4 | GSMR | 9 | 0.06 (0.02, 0.10) | 3.5E-03 |
| LPA | P08519 | OID30747 | TC | 5.00E-06 | 0.1 | GSMR | 3 | 0.06 (0.02, 0.10) | 4.7E-03 |
| LPA | P08519 | OID30747 | TC | 1.00E-04 | 0.4 | GSMR | 9 | 0.04 (0.01, 0.08) | 0.03 |
| PCSK7 | Q16549 | OID31410 | TG | 5.00E-06 | 0.1 | GSMR | 4 | -0.11 (-0.15, -0.07) | 1.1E-07 |
| PCSK7 | Q16549 | OID31410 | TG | 1.00E-04 | 0.4 | GSMR | 16 | -0.04 (-0.07, -0.00) | 0.03 |
| PCSK9 | Q8NBP7 | OID20235 | LDL | 1.00E-04 | 0.4 | GSMR | 6 | 0.1 (0.05, 0.15) | 6.2E-05 |
| PCSK9 | Q8NBP7 | OID20235 | nonHDL | 1.00E-04 | 0.4 | GSMR | 6 | 0.08 (0.03, 0.13) | 9.1E-04 |
| PCSK9 | Q8NBP7 | OID20235 | TC | 1.00E-04 | 0.4 | GSMR | 6 | 0.07 (0.03, 0.12) | 2.5E-03 |
| PECR | Q9BY49 | OID30228 | HDL | 5.00E-06 | 0.1 | GSMR | 3 | -0.05 (-0.08, -0.02) | 1.0E-03 |
| PECR | Q9BY49 | OID30228 | HDL | 1.00E-04 | 0.4 | GSMR | 7 | -0.04 (-0.07, -0.01) | 8.4E-03 |
| PLA2G15 | Q8NCC3 | OID21473 | HDL | 1.00E-04 | 0.4 | GSMR | 4 | -0.13 (-0.20, -0.07) | 2.5E-05 |
| PLTP | P55058 | OID20238 | TG | 1.00E-04 | 0.4 | GSMR | 14 | -0.09 (-0.12, -0.05) | 6.4E-07 |
| THOP1 | P52888 | OID20194 | HDL | 1.00E-04 | 0.4 | GSMR | 7 | 0.05 (0.01, 0.09) | 0.02 |
| TLR3 | O15455 | OID20612 | HDL | 1.00E-04 | 0.4 | GSMR | 33 | 0.01 (0.00, 0.02) | 0.05 |
| TLR3 | O15455 | OID20612 | HDL | 5.00E-06 | 0.1 | GSMR | 10 | -0.01 (-0.02, 0.00) | 0.18 |
| TNFSF13 | O75888 | OID20733 | nonHDL | 1.00E-04 | 0.4 | GSMR | 3 | -0.07 (-0.12, -0.03) | 1.0E-03 |
| TNFSF13 | O75888 | OID20733 | TC | 1.00E-04 | 0.4 | GSMR | 3 | -0.06 (-0.10, -0.01) | 8.1E-03 |

# **Supplementary Table S6. MR estimates of plasma proteins’ effect on cardiovascular diseases**

| Protein | UniProtID | ProbeID | Outcome | Method | n SNPs | Beta (95% Confidence Interval) | P | FDR |
| --- | --- | --- | --- | --- | --- | --- | --- | --- |
| ADAMTSL5 | Q6ZMM2 | OID30355 | LAS | Wald ratio | 1 | 0.53 (0.28, 1.00) | 0.05 | 0.46 |
| ADAMTSL5 | Q6ZMM2 | OID30355 | CES | Wald ratio | 1 | 0.67 (0.37, 1.22) | 0.19 | 0.56 |
| ADAMTSL5 | Q6ZMM2 | OID30355 | stroke | Wald ratio | 1 | 0.89 (0.66, 1.21) | 0.47 | 0.93 |
| ADAMTSL5 | Q6ZMM2 | OID30355 | IS | Wald ratio | 1 | 0.89 (0.66, 1.21) | 0.47 | 0.93 |
| ADAMTSL5 | Q6ZMM2 | OID30355 | SVS | Wald ratio | 1 | 0.89 (0.46, 1.74) | 0.74 | 0.97 |
| ADAMTSL5 | Q6ZMM2 | OID30355 | CAD | Wald ratio | 1 | 1.14 (0.78, 1.66) | 0.50 | 1.00 |
| ANGPTL3 | Q9Y5C1 | OID20407 | SVS | Wald ratio | 1 | 0.71 (0.41, 1.24) | 0.23 | 0.45 |
| ANGPTL3 | Q9Y5C1 | OID20407 | LAS | Wald ratio | 1 | 0.70 (0.41, 1.19) | 0.19 | 0.46 |
| ANGPTL3 | Q9Y5C1 | OID20407 | CES | Wald ratio | 1 | 0.59 (0.35, 0.98) | 0.04 | 0.55 |
| ANGPTL3 | Q9Y5C1 | OID20407 | CAD | Wald ratio | 1 | 0.94 (0.67, 1.31) | 0.72 | 1.00 |
| ANGPTL4 | Q9BY76 | OID20703 | SVS | Wald ratio | 1 | 0.66 (0.37, 1.21) | 0.18 | 0.42 |
| ANGPTL4 | Q9BY76 | OID20703 | CES | Wald ratio | 1 | 1.07 (0.63, 1.81) | 0.81 | 0.90 |
| ANGPTL4 | Q9BY76 | OID20703 | LAS | Wald ratio | 1 | 0.92 (0.52, 1.62) | 0.77 | 0.90 |
| ANGPTL4 | Q9BY76 | OID20703 | stroke | Wald ratio | 1 | 1.00 (0.76, 1.31) | 1.00 | 1.00 |
| ANGPTL4 | Q9BY76 | OID20703 | IS | Wald ratio | 1 | 1.00 (0.76, 1.31) | 1.00 | 1.00 |
| ANGPTL4 | Q9BY76 | OID20703 | CAD | Wald ratio | 1 | 1.19 (0.84, 1.68) | 0.34 | 1.00 |
| APOC1 | P02654 | OID30749 | LAS | Wald ratio | 1 | 1.45 (0.82, 2.54) | 0.20 | 0.46 |
| APOC1 | P02654 | OID30749 | CES | Wald ratio | 1 | 1.10 (0.65, 1.85) | 0.72 | 0.85 |
| APOC1 | P02654 | OID30749 | SVS | Wald ratio | 1 | 0.96 (0.53, 1.75) | 0.90 | 0.97 |
| APOC1 | P02654 | OID30749 | stroke | Wald ratio | 1 | 0.99 (0.75, 1.30) | 0.94 | 1.00 |
| APOC1 | P02654 | OID30749 | IS | Wald ratio | 1 | 0.99 (0.75, 1.30) | 0.94 | 1.00 |
| APOC1 | P02654 | OID30749 | CAD | Wald ratio | 1 | 1.02 (0.71, 1.46) | 0.90 | 1.00 |
| APOE | P02649 | OID30727 | SVS | Wald ratio | 1 | 0.81 (0.54, 1.20) | 0.29 | 0.48 |
| APOE | P02649 | OID30727 | CES | Wald ratio | 1 | 0.75 (0.51, 1.09) | 0.13 | 0.55 |
| APOE | P02649 | OID30727 | CAD | Wald ratio | 1 | 0.86 (0.70, 1.04) | 0.12 | 0.55 |
| APOE | P02649 | OID30727 | LAS | Wald ratio | 1 | 0.95 (0.65, 1.40) | 0.80 | 0.90 |
| APOE | P02649 | OID30727 | stroke | Wald ratio | 1 | 0.98 (0.82, 1.17) | 0.83 | 1.00 |
| APOE | P02649 | OID30727 | IS | Wald ratio | 1 | 0.98 (0.82, 1.17) | 0.83 | 1.00 |
| CELSR2 | Q9HCU4 | OID30593 | CAD | Wald ratio | 1 | 0.64 (0.50, 0.81) | 2.0E-04 | 5.5E-03 |
| CELSR2 | Q9HCU4 | OID30593 | LAS | Wald ratio | 1 | 1.16 (0.77, 1.74) | 0.47 | 0.70 |
| CELSR2 | Q9HCU4 | OID30593 | CES | Wald ratio | 1 | 0.86 (0.59, 1.26) | 0.44 | 0.72 |
| CELSR2 | Q9HCU4 | OID30593 | stroke | Wald ratio | 1 | 0.95 (0.77, 1.16) | 0.59 | 0.93 |
| CELSR2 | Q9HCU4 | OID30593 | IS | Wald ratio | 1 | 0.95 (0.77, 1.16) | 0.59 | 0.93 |
| CELSR2 | Q9HCU4 | OID30593 | SVS | Wald ratio | 1 | 0.93 (0.61, 1.43) | 0.75 | 0.97 |
| CGREF1 | Q99674 | OID20152 | CAD | Wald ratio | 1 | 0.74 (0.58, 0.95) | 0.02 | 0.14 |
| CGREF1 | Q99674 | OID20152 | stroke | Wald ratio | 1 | 0.81 (0.65, 1.01) | 0.06 | 0.29 |
| CGREF1 | Q99674 | OID20152 | IS | Wald ratio | 1 | 0.81 (0.65, 1.01) | 0.06 | 0.29 |
| CGREF1 | Q99674 | OID20152 | SVS | Wald ratio | 1 | 0.66 (0.41, 1.07) | 0.09 | 0.29 |
| CGREF1 | Q99674 | OID20152 | LAS | Wald ratio | 1 | 0.78 (0.49, 1.24) | 0.30 | 0.56 |
| CGREF1 | Q99674 | OID20152 | CES | Wald ratio | 1 | 0.88 (0.57, 1.33) | 0.54 | 0.73 |
| DPEP2 | Q9H4A9 | OID21305 | stroke | Wald ratio | 1 | 1.28 (1.07, 1.53) | 6.9E-03 | 0.07 |
| DPEP2 | Q9H4A9 | OID21305 | IS | Wald ratio | 1 | 1.28 (1.07, 1.53) | 6.9E-03 | 0.07 |
| DPEP2 | Q9H4A9 | OID21305 | SVS | Wald ratio | 1 | 1.41 (0.93, 2.14) | 0.10 | 0.29 |
| DPEP2 | Q9H4A9 | OID21305 | LAS | Wald ratio | 1 | 1.41 (0.95, 2.09) | 0.09 | 0.46 |
| DPEP2 | Q9H4A9 | OID21305 | CES | Wald ratio | 1 | 1.25 (0.88, 1.77) | 0.21 | 0.56 |
| DPEP2 | Q9H4A9 | OID21305 | CAD | Wald ratio | 1 | 0.97 (0.80, 1.19) | 0.79 | 1.00 |
| EPPK1 | P58107 | OID30250 | stroke | Wald ratio | 1 | 1.29 (0.98, 1.69) | 0.07 | 0.29 |
| EPPK1 | P58107 | OID30250 | IS | Wald ratio | 1 | 1.29 (0.98, 1.69) | 0.07 | 0.29 |
| EPPK1 | P58107 | OID30250 | SVS | Wald ratio | 1 | 1.39 (0.80, 2.40) | 0.24 | 0.45 |
| EPPK1 | P58107 | OID30250 | LAS | Wald ratio | 1 | 1.47 (0.86, 2.50) | 0.16 | 0.46 |
| EPPK1 | P58107 | OID30250 | CES | Wald ratio | 1 | 1.32 (0.80, 2.17) | 0.28 | 0.67 |
| EPPK1 | P58107 | OID30250 | CAD | Wald ratio | 1 | 0.92 (0.68, 1.25) | 0.58 | 1.00 |
| GAS6 | Q14393 | OID20318 | LAS | Wald ratio | 1 | 1.55 (0.98, 2.44) | 0.06 | 0.46 |
| GAS6 | Q14393 | OID20318 | stroke | Wald ratio | 1 | 1.17 (0.94, 1.46) | 0.17 | 0.53 |
| GAS6 | Q14393 | OID20318 | IS | Wald ratio | 1 | 1.17 (0.94, 1.46) | 0.17 | 0.53 |
| GAS6 | Q14393 | OID20318 | CES | Wald ratio | 1 | 0.69 (0.42, 1.15) | 0.16 | 0.55 |
| GAS6 | Q14393 | OID20318 | SVS | Wald ratio | 1 | 1.00 (0.62, 1.60) | 0.99 | 0.99 |
| GAS6 | Q14393 | OID20318 | CAD | Wald ratio | 1 | 1.01 (0.75, 1.36) | 0.95 | 1.00 |
| GIPC3 | Q8TF64 | OID31507 | SVS | Wald ratio | 1 | 0.65 (0.33, 1.31) | 0.23 | 0.45 |
| GIPC3 | Q8TF64 | OID31507 | CAD | Wald ratio | 1 | 1.38 (0.92, 2.06) | 0.12 | 0.55 |
| GIPC3 | Q8TF64 | OID31507 | CES | Wald ratio | 1 | 1.24 (0.66, 2.33) | 0.50 | 0.72 |
| GIPC3 | Q8TF64 | OID31507 | LAS | Wald ratio | 1 | 0.99 (0.51, 1.91) | 0.97 | 0.97 |
| GSTA1 | P08263 | OID20166 | LAS | Wald ratio | 1 | 1.42 (0.86, 2.34) | 0.17 | 0.46 |
| GSTA1 | P08263 | OID20166 | CES | Wald ratio | 1 | 1.17 (0.73, 1.87) | 0.51 | 0.72 |
| GSTA1 | P08263 | OID20166 | SVS | Wald ratio | 1 | 1.04 (0.62, 1.76) | 0.87 | 0.97 |
| GSTA1 | P08263 | OID20166 | stroke | Wald ratio | 1 | 1.02 (0.78, 1.33) | 0.88 | 1.00 |
| GSTA1 | P08263 | OID20166 | IS | Wald ratio | 1 | 1.02 (0.78, 1.33) | 0.88 | 1.00 |
| GSTA1 | P08263 | OID20166 | CAD | Wald ratio | 1 | 0.92 (0.67, 1.26) | 0.61 | 1.00 |
| GSTA3 | Q16772 | OID21242 | LAS | Wald ratio | 1 | 1.35 (0.88, 2.09) | 0.17 | 0.46 |
| GSTA3 | Q16772 | OID21242 | CES | Wald ratio | 1 | 1.15 (0.77, 1.71) | 0.51 | 0.72 |
| GSTA3 | Q16772 | OID21242 | SVS | Wald ratio | 1 | 1.04 (0.66, 1.63) | 0.87 | 0.97 |
| GSTA3 | Q16772 | OID21242 | stroke | Wald ratio | 1 | 1.02 (0.82, 1.27) | 0.88 | 1.00 |
| GSTA3 | Q16772 | OID21242 | IS | Wald ratio | 1 | 1.02 (0.82, 1.27) | 0.88 | 1.00 |
| GSTA3 | Q16772 | OID21242 | CAD | Wald ratio | 1 | 0.93 (0.71, 1.22) | 0.61 | 1.00 |
| HGFAC | Q04756 | OID30718 | SVS | Wald ratio | 1 | 0.71 (0.46, 1.11) | 0.14 | 0.35 |
| HGFAC | Q04756 | OID30718 | CES | Wald ratio | 1 | 0.75 (0.51, 1.12) | 0.16 | 0.55 |
| HGFAC | Q04756 | OID30718 | LAS | Wald ratio | 1 | 1.04 (0.68, 1.58) | 0.86 | 0.92 |
| HGFAC | Q04756 | OID30718 | CAD | Wald ratio | 1 | 0.90 (0.68, 1.19) | 0.46 | 1.00 |
| KHK | P50053 | OID30241 | stroke | Wald ratio | 1 | 1.40 (1.09, 1.79) | 9.3E-03 | 0.07 |
| KHK | P50053 | OID30241 | IS | Wald ratio | 1 | 1.40 (1.09, 1.79) | 9.3E-03 | 0.07 |
| KHK | P50053 | OID30241 | CAD | Wald ratio | 1 | 1.30 (1.04, 1.63) | 0.02 | 0.14 |
| KHK | P50053 | OID30241 | SVS | Wald ratio | 1 | 1.42 (0.94, 2.14) | 0.10 | 0.29 |
| KHK | P50053 | OID30241 | LAS | Wald ratio | 1 | 1.31 (0.78, 2.20) | 0.30 | 0.56 |
| KHK | P50053 | OID30241 | CES | Wald ratio | 1 | 1.15 (0.79, 1.67) | 0.46 | 0.72 |
| LILRA3 | Q8N6C8 | OID30376 | SVS | Wald ratio | 1 | 0.82 (0.67, 1.00) | 0.05 | 0.29 |
| LILRA3 | Q8N6C8 | OID30376 | LAS | Wald ratio | 1 | 1.08 (0.89, 1.31) | 0.42 | 0.65 |
| LILRA3 | Q8N6C8 | OID30376 | CES | Wald ratio | 1 | 1.02 (0.85, 1.22) | 0.83 | 0.90 |
| LILRA3 | Q8N6C8 | OID30376 | stroke | Wald ratio | 1 | 0.96 (0.88, 1.05) | 0.38 | 0.91 |
| LILRA3 | Q8N6C8 | OID30376 | IS | Wald ratio | 1 | 0.96 (0.88, 1.05) | 0.38 | 0.91 |
| LILRA3 | Q8N6C8 | OID30376 | CAD | Wald ratio | 1 | 1.00 (0.91, 1.10) | 1.00 | 1.00 |
| LILRA5 | A6NI73 | OID20209 | SVS | Wald ratio | 1 | 0.39 (0.20, 0.76) | 5.7E-03 | 0.12 |
| LILRA5 | A6NI73 | OID20209 | CAD | Wald ratio | 1 | 0.66 (0.48, 0.91) | 0.01 | 0.14 |
| LILRA5 | A6NI73 | OID20209 | LAS | Wald ratio | 1 | 1.16 (0.61, 2.23) | 0.65 | 0.87 |
| LILRB2 | Q8N423 | OID20301 | SVS | Wald ratio | 1 | 0.74 (0.60, 0.93) | 8.6E-03 | 0.12 |
| LILRB2 | Q8N423 | OID20301 | LAS | Wald ratio | 1 | 1.03 (0.84, 1.27) | 0.77 | 0.90 |
| LILRB2 | Q8N423 | OID20301 | stroke | Wald ratio | 1 | 0.96 (0.85, 1.08) | 0.51 | 0.93 |
| LILRB2 | Q8N423 | OID20301 | IS | Wald ratio | 1 | 0.96 (0.85, 1.08) | 0.51 | 0.93 |
| LILRB2 | Q8N423 | OID20301 | CES | Wald ratio | 1 | 1.00 (0.82, 1.21) | 0.97 | 0.97 |
| LILRB2 | Q8N423 | OID20301 | CAD | Wald ratio | 1 | 1.01 (0.89, 1.15) | 0.86 | 1.00 |
| LILRB5 | O75023 | OID20324 | SVS | Wald ratio | 1 | 1.16 (0.97, 1.38) | 0.10 | 0.29 |
| LILRB5 | O75023 | OID20324 | LAS | Wald ratio | 1 | 0.88 (0.74, 1.05) | 0.16 | 0.46 |
| LILRB5 | O75023 | OID20324 | CES | Wald ratio | 1 | 1.12 (0.96, 1.31) | 0.15 | 0.55 |
| LILRB5 | O75023 | OID20324 | stroke | Wald ratio | 1 | 1.02 (0.92, 1.13) | 0.71 | 0.97 |
| LILRB5 | O75023 | OID20324 | IS | Wald ratio | 1 | 1.02 (0.92, 1.13) | 0.71 | 0.97 |
| LILRB5 | O75023 | OID20324 | CAD | Wald ratio | 1 | 0.98 (0.89, 1.08) | 0.66 | 1.00 |
| LPA | P08519 | OID30747 | stroke | Wald ratio | 1 | 1.45 (1.10, 1.90) | 7.8E-03 | 0.07 |
| LPA | P08519 | OID30747 | IS | Wald ratio | 1 | 1.45 (1.10, 1.90) | 7.8E-03 | 0.07 |
| LPA | P08519 | OID30747 | SVS | Wald ratio | 1 | 1.70 (0.94, 3.08) | 0.08 | 0.29 |
| LPA | P08519 | OID30747 | LAS | Wald ratio | 1 | 1.62 (0.91, 2.90) | 0.10 | 0.46 |
| LPA | P08519 | OID30747 | CES | Wald ratio | 1 | 1.72 (1.00, 2.95) | 0.05 | 0.55 |
| LPA | P08519 | OID30747 | CAD | Wald ratio | 1 | 0.88 (0.64, 1.20) | 0.42 | 1.00 |
| PCSK7 | Q16549 | OID31410 | CES | Wald ratio | 1 | 1.03 (0.71, 1.51) | 0.86 | 0.90 |
| PCSK7 | Q16549 | OID31410 | stroke | Wald ratio | 1 | 1.05 (0.86, 1.28) | 0.63 | 0.93 |
| PCSK7 | Q16549 | OID31410 | IS | Wald ratio | 1 | 1.05 (0.86, 1.28) | 0.63 | 0.93 |
| PCSK7 | Q16549 | OID31410 | LAS | Wald ratio | 1 | 1.02 (0.68, 1.53) | 0.93 | 0.97 |
| PCSK7 | Q16549 | OID31410 | SVS | Wald ratio | 1 | 0.98 (0.64, 1.51) | 0.94 | 0.98 |
| PCSK7 | Q16549 | OID31410 | CAD | Wald ratio | 1 | 1.02 (0.80, 1.31) | 0.85 | 1.00 |
| PCSK9 | Q8NBP7 | OID20235 | LAS | Wald ratio | 1 | 0.63 (0.33, 1.18) | 0.15 | 0.46 |
| PCSK9 | Q8NBP7 | OID20235 | SVS | Wald ratio | 1 | 0.72 (0.37, 1.41) | 0.34 | 0.53 |
| PCSK9 | Q8NBP7 | OID20235 | CES | Wald ratio | 1 | 1.68 (0.93, 3.02) | 0.08 | 0.55 |
| PCSK9 | Q8NBP7 | OID20235 | stroke | Wald ratio | 1 | 1.01 (0.75, 1.36) | 0.96 | 1.00 |
| PCSK9 | Q8NBP7 | OID20235 | IS | Wald ratio | 1 | 1.01 (0.75, 1.36) | 0.96 | 1.00 |
| PCSK9 | Q8NBP7 | OID20235 | CAD | Wald ratio | 1 | 1.00 (0.70, 1.43) | 0.99 | 1.00 |
| PECR | Q9BY49 | OID30228 | CAD | Wald ratio | 1 | 1.21 (0.94, 1.54) | 0.14 | 0.55 |
| PECR | Q9BY49 | OID30228 | LAS | Wald ratio | 1 | 0.85 (0.57, 1.26) | 0.41 | 0.65 |
| PECR | Q9BY49 | OID30228 | CES | Wald ratio | 1 | 1.14 (0.79, 1.65) | 0.47 | 0.72 |
| PECR | Q9BY49 | OID30228 | stroke | Wald ratio | 1 | 1.05 (0.87, 1.26) | 0.63 | 0.93 |
| PECR | Q9BY49 | OID30228 | IS | Wald ratio | 1 | 1.05 (0.87, 1.26) | 0.63 | 0.93 |
| PECR | Q9BY49 | OID30228 | SVS | Wald ratio | 1 | 0.94 (0.62, 1.42) | 0.77 | 0.97 |
| PLA2G15 | Q8NCC3 | OID21473 | SVS | Wald ratio | 1 | 1.92 (1.01, 3.67) | 0.05 | 0.29 |
| PLA2G15 | Q8NCC3 | OID21473 | CES | Wald ratio | 1 | 1.50 (0.85, 2.66) | 0.16 | 0.55 |
| PLA2G15 | Q8NCC3 | OID21473 | LAS | Wald ratio | 1 | 1.31 (0.71, 2.42) | 0.39 | 0.65 |
| PLA2G15 | Q8NCC3 | OID21473 | CAD | Wald ratio | 1 | 0.98 (0.67, 1.44) | 0.91 | 1.00 |
| PLTP | P55058 | OID20238 | SVS | Wald ratio | 1 | 0.65 (0.41, 1.04) | 0.07 | 0.29 |
| PLTP | P55058 | OID20238 | LAS | Wald ratio | 1 | 1.29 (0.83, 2.01) | 0.25 | 0.54 |
| PLTP | P55058 | OID20238 | CES | Wald ratio | 1 | 0.92 (0.62, 1.39) | 0.70 | 0.85 |
| PLTP | P55058 | OID20238 | CAD | Wald ratio | 1 | 0.94 (0.74, 1.18) | 0.59 | 1.00 |
| THOP1 | P52888 | OID20194 | stroke | Wald ratio | 1 | 0.82 (0.62, 1.08) | 0.17 | 0.53 |
| THOP1 | P52888 | OID20194 | IS | Wald ratio | 1 | 0.82 (0.62, 1.08) | 0.17 | 0.53 |
| THOP1 | P52888 | OID20194 | CES | Wald ratio | 1 | 0.76 (0.46, 1.27) | 0.30 | 0.67 |
| THOP1 | P52888 | OID20194 | SVS | Wald ratio | 1 | 0.84 (0.47, 1.51) | 0.57 | 0.83 |
| THOP1 | P52888 | OID20194 | LAS | Wald ratio | 1 | 0.89 (0.50, 1.59) | 0.70 | 0.88 |
| THOP1 | P52888 | OID20194 | CAD | Wald ratio | 1 | 0.87 (0.62, 1.22) | 0.42 | 1.00 |
| TLR3 | O15455 | OID20612 | CES | Wald ratio | 1 | 0.96 (0.83, 1.11) | 0.57 | 0.73 |
| TLR3 | O15455 | OID20612 | LAS | Wald ratio | 1 | 0.96 (0.83, 1.13) | 0.65 | 0.87 |
| TLR3 | O15455 | OID20612 | stroke | IVW | 2 | 0.96 (0.88, 1.05) | 0.38 | 0.91 |
| TLR3 | O15455 | OID20612 | IS | IVW | 2 | 0.96 (0.88, 1.05) | 0.38 | 0.91 |
| TLR3 | O15455 | OID20612 | SVS | Wald ratio | 1 | 0.99 (0.84, 1.16) | 0.90 | 0.97 |
| TLR3 | O15455 | OID20612 | CAD | Wald ratio | 1 | 1.03 (0.94, 1.12) | 0.56 | 1.00 |
| TNFSF13 | O75888 | OID20733 | SVS | Wald ratio | 1 | 1.42 (0.77, 2.59) | 0.26 | 0.45 |
| TNFSF13 | O75888 | OID20733 | LAS | Wald ratio | 1 | 0.66 (0.38, 1.17) | 0.16 | 0.46 |
| TNFSF13 | O75888 | OID20733 | CES | Wald ratio | 1 | 1.27 (0.74, 2.19) | 0.38 | 0.72 |
| TNFSF13 | O75888 | OID20733 | stroke | Wald ratio | 1 | 1.12 (0.85, 1.49) | 0.42 | 0.91 |
| TNFSF13 | O75888 | OID20733 | IS | Wald ratio | 1 | 1.12 (0.85, 1.49) | 0.42 | 0.91 |
| TNFSF13 | O75888 | OID20733 | CAD | Wald ratio | 1 | 1.06 (0.74, 1.51) | 0.76 | 1.00 |

# **Supplementary Table S7. Colocalisation between plasma proteins and cardiovascular diseases**

| **Protein** | **UniProtID** | **ProbeID** | **Outcome** | **N SNPs** | **PPH0** | **PPH1** | **PPH2** | **PPH3** | **PPH4** |
| --- | --- | --- | --- | --- | --- | --- | --- | --- | --- |
| CELSR2 | Q9HCU4 | OID30593 | CAD | 1302 | 6.6E-12 | 0.04 | 3.7E-12 | 0.02 | 0.94 |

# **Supplementary Table S8. Generalised Mendelian randomisation result of CELSR2’s effect on CAD**

| **Protein** | **UniProtID** | **ProbeID** | **Outcome** | **Method** | **n SNPs** | **Odds ratio (95% Confidence Interval)** | **P** | **Intercept** | **P (Intercept)** |
| --- | --- | --- | --- | --- | --- | --- | --- | --- | --- |
| CELSR2 | Q9HCU4 | OID30593 | CAD | gIVW | 5 | 0.70 (0.50, 0.96) | 0.03 | NA | NA |
| CELSR2 | Q9HCU4 | OID30593 | CAD | gEgger | 5 | 0.59 (0.29, 1.21) | 0.15 | 0.07 | 0.61 |
| CELSR2 | Q9HCU4 | OID30593 | CAD | weighted median | 5 | 0.66 (0.52, 0.82) | 2.3E-4 | NA | NA |

# **Supplementary Table S9. Mendelian randomisation result of lipid fractions’ effect on cardiovascular diseases**

| **Outcome** | **Exposure** | **Method** | **n SNPs** | **Odds ratio (95% confidence interval)** | **P** | **FDR** | **Intercept** | **P (Intercept)** |
| --- | --- | --- | --- | --- | --- | --- | --- | --- |
| CAD | HDL-C | IVW | 19 | 0.9(0.65, 1.25) | 0.53 | 0.66 | NA | NA |
| CAD | LDL-C | IVW | 14 | 1.64(1.03, 2.62) | 0.04 | 0.09 | NA | NA |
| CAD | LDL-C | MR-Egger | 14 | 2.51(1.04, 6.04) | 0.06 | NA | -0.04 | 0.29 |
| CAD | LDL-C | Weighted median | 14 | 1.87(1.05, 3.33) | 0.03 | NA | NA | NA |
| CAD | Non-HDL-C | IVW | 13 | 1.73(1.1, 2.72) | 0.02 | 0.08 | NA | NA |
| CAD | Non-HDL-C | MR-Egger | 13 | 3.34(1.22, 9.11) | 0.04 | NA | -0.06 | 0.18 |
| CAD | Non-HDL-C | Weighted median | 13 | 2.08(1.19, 3.64) | 0.01 | NA | NA | NA |
| CAD | TC | IVW | 15 | 1.53(0.91, 2.58) | 0.11 | 0.18 | NA | NA |
| CAD | TG | IVW | 12 | 1.01(0.77, 1.32) | 0.96 | 0.96 | NA | NA |
| cardioembolic stroke | HDL-C | IVW | 18 | 0.72(0.45, 1.17) | 0.19 | 0.46 | NA | NA |
| cardioembolic stroke | LDL-C | IVW | 14 | 1.78(1.01, 3.15) | 0.05 | 0.24 | NA | NA |
| cardioembolic stroke | LDL-C | MR-Egger | 14 | 2.99(0.99, 9.03) | 0.08 | NA | -0.05 | 0.30 |
| cardioembolic stroke | LDL-C | Weighted median | 14 | 1.92(0.85, 4.34) | 0.11 | NA | NA | NA |
| cardioembolic stroke | nonHDL-C | IVW | 11 | 1.06(0.57, 1.96) | 0.86 | 0.96 | NA | NA |
| cardioembolic stroke | TC | IVW | 15 | 1.02(0.54, 1.91) | 0.96 | 0.96 | NA | NA |
| cardioembolic stroke | TG | IVW | 13 | 0.84(0.51, 1.36) | 0.47 | 0.78 | NA | NA |
| ischemic stroke | HDL-C | IVW | 18 | 1.05(0.79, 1.4) | 0.74 | 1.00 | NA | NA |
| ischemic stroke | LDL-C | IVW | 13 | 1.32(0.94, 1.85) | 0.11 | 0.34 | NA | NA |
| ischemic stroke | nonHDL-C | IVW | 13 | 1(0.69, 1.44) | 1.00 | 1.00 | NA | NA |
| ischemic stroke | TC | IVW | 16 | 0.97(0.66, 1.43) | 0.87 | 1.00 | NA | NA |
| ischemic stroke | TG | IVW | 15 | 0.78(0.56, 1.08) | 0.14 | 0.34 | NA | NA |
| large artery stroke | HDL-C | IVW | 17 | 1.11(0.67, 1.84) | 0.69 | 0.69 | NA | NA |
| large artery stroke | LDL-C | IVW | 12 | 1.4(0.74, 2.63) | 0.30 | 0.69 | NA | NA |
| large artery stroke | nonHDL-C | IVW | 11 | 1.29(0.65, 2.58) | 0.47 | 0.69 | NA | NA |
| large artery stroke | TC | IVW | 15 | 1.16(0.59, 2.29) | 0.67 | 0.69 | NA | NA |
| large artery stroke | TG | IVW | 16 | 0.59(0.36, 0.98) | 0.04 | 0.21 | NA | NA |
| large artery stroke | TG | MR-Egger | 16 | 1.03(0.31, 3.41) | 0.97 | NA | -0.06 | 0.34 |
| large artery stroke | TG | Weighted median | 16 | 0.43(0.22, 0.84) | 0.01 | NA | NA | NA |
| small vessel stroke | HDL-C | IVW | 13 | 1.2(0.69, 2.09) | 0.52 | 0.65 | NA | NA |
| small vessel stroke | LDL-C | IVW | 9 | 0.69(0.31, 1.53) | 0.36 | 0.60 | NA | NA |
| small vessel stroke | nonHDL-C | IVW | 10 | 0.71(0.35, 1.45) | 0.35 | 0.60 | NA | NA |
| small vessel stroke | TC | IVW | 11 | 0.69(0.32, 1.5) | 0.35 | 0.60 | NA | NA |
| small vessel stroke | TG | IVW | 12 | 0.96(0.53, 1.74) | 0.90 | 0.90 | NA | NA |
| stroke | HDL-C | IVW | 17 | 0.99(0.75, 1.3) | 0.93 | 1.00 | NA | NA |
| stroke | LDL-C | IVW | 13 | 1.32(0.94, 1.85) | 0.11 | 0.34 | NA | NA |
| stroke | nonHDL-C | IVW | 13 | 1(0.69, 1.44) | 1.00 | 1.00 | NA | NA |
| stroke | TC | IVW | 16 | 0.97(0.66, 1.43) | 0.87 | 1.00 | NA | NA |
| stroke | TG | IVW | 15 | 0.78(0.56, 1.08) | 0.14 | 0.34 | NA | NA |

# **Supplementary Table S10. Multi-trait colocalisation between CELSR2, LDL-C, and CAD**

| **Configuration** | **Posterior Probability** |
| --- | --- |
| A causal variant for CELSR2, no causal variant for LDL-C and CAD | 6.3E-93 |
| Distinct causal variants for CELSR2 and LDL-C, no causal variant for CAD | 5.8E-04 |
| Distinct causal variants for CELSR2, LDL-C, and CAD | 1.3E-04 |
| A distinct causal variant for CELSR2, a shared causal variant for LDL-C and CAD | 4.0E-03 |
| Distinct causal variants for CELSR2 and CAD, no causal variant for LDL-C | 9.6E-94 |
| A shared causal variant for CELSR2 and LDL-C, no causal variant for CAD | 0.25 |
| A shared causal variant for CELSR2 and LDL-C, a distinct causal variant for CAD | 0.04 |
| A shared causal variant for CELSR2, LDL-C, and CAD | 0.70 |
| A shared causal variant for CELSR2 and CAD, no causal variant for LDL-C | 2.0E-92 |
| A shared causal variant for CELSR2 and CAD, a distinct causal variant for LDL-C | 2.0E-03 |
| A causal variant for LDL-C, no causal variant for CELSR2 and CAD | 1.7E-14 |
| Distinct causal variants for LDL-C and CAD, no causal variant for CELSR2 | 2.6E-15 |
| A shared causal variant for LDL-C and CAD, a distinct causal variant for CELSR2 | 9.7E-14 |
| A causal variant for CAD, no causal variant for LDL-C and CELSR2 | 2.0E-104 |
| No causal variant for CELSR2, LDL-C, or CAD | 1.3E-103 |

# **Supplementary Table S11. Significant Mendelian randomisation result of lipid fractions’ effect on plasma protein**

| **Protein** | **UniProtID** | **ProbeID** | **Exposure** | **Method** | **n SNPs** | **Beta (95% Confidence Interval)** | **P** | **FDR** |
| --- | --- | --- | --- | --- | --- | --- | --- | --- |
| LDLR | P01130 | OID20240 | TG | IVW | 8 | 0.60 (0.34, 0.86) | 6.7E-06 | 0.02 |
| FGFBP2 | Q9BYJ0 | OID30395 | HDL-C | IVW | 14 | 0.61 (0.34, 0.87) | 8.4E-06 | 0.02 |
| APOA1 | P02647 | OID30769 | HDL-C | IVW | 12 | 0.55 (0.29, 0.82) | 4.5E-05 | 0.05 |
| MENT | Q9BUN1 | OID30595 | HDL-C | IVW | 16 | 0.56 (0.29, 0.83) | 5.0E-05 | 0.05 |

# **Supplementary Table S12. MR-Egger and weighted median result of lipid fractions’ effect on plasma protein**

| **Protein** | **UniProtID** | **ProbeID** | **Exposure** | **Method** | **n SNPs** | **Beta** | **Standard Error** | **P** | **Intercept** | **P (Intercept)** |
| --- | --- | --- | --- | --- | --- | --- | --- | --- | --- | --- |
| FGFBP2 | Q9BYJ0 | OID30395 | HDL | Weighted median | 14 | 0.76 | 0.17 | 9.5E-06 | NA | NA |
| APOA1 | P02647 | OID30769 | HDL | Weighted median | 12 | 0.69 | 0.16 | 2.2E-05 | NA | NA |
| LDLR | P01130 | OID20240 | TG | Weighted median | 8 | 0.66 | 0.16 | 4.2E-05 | NA | NA |
| MENT | Q9BUN1 | OID30595 | HDL | Weighted median | 16 | 0.60 | 0.18 | 7.4E-04 | NA | NA |
| APOA1 | P02647 | OID30769 | HDL | MR Egger | 12 | 0.95 | 0.28 | 6.6E-03 | -0.05 | 0.13 |
| FGFBP2 | Q9BYJ0 | OID30395 | HDL | MR Egger | 14 | 0.79 | 0.26 | 0.01 | -0.02 | 0.42 |
| LDLR | P01130 | OID20240 | TG | MR Egger | 8 | 0.83 | 0.28 | 0.02 | -0.04 | 0.39 |
| MENT | Q9BUN1 | OID30595 | HDL | MR Egger | 16 | 0.69 | 0.28 | 0.03 | -0.02 | 0.60 |

# **Supplementary Table S13. Mendelian randomisation result of plasma proteins’ effect on lipid fractions in European**

| Protein | UniProtID | ProbeID | Outcome | Colocalisation evidence in South Asian | GMR evidence_in South Asian | Beta (95% Confidence Interval) | P | FDR |
| --- | --- | --- | --- | --- | --- | --- | --- | --- |
| ADAMTSL5 | Q6ZMM2 | OID30355 | TC | FALSE | Unapplicable | 0 (-0.02, 0.01) | 0.88 | 0.88 |
| ANGPTL3 | Q9Y5C1 | OID20407 | HDL-C | Suggestive | TRUE | 0.04 (0.03, 0.05) | 4.2E-12 | 3.4E-11 |
| ANGPTL3 | Q9Y5C1 | OID20407 | LDL-C | Strong | TRUE | 0.14 (0.1, 0.18) | 6.6E-12 | 2.3E-11 |
| ANGPTL3 | Q9Y5C1 | OID20407 | Non-HDL-C | Strong | TRUE | 0.21 (0.17, 0.26) | 6.8E-18 | 2.0E-17 |
| ANGPTL3 | Q9Y5C1 | OID20407 | TC | Strong | TRUE | 0.22 (0.17, 0.27) | 5.0E-17 | 1.8E-16 |
| ANGPTL3 | Q9Y5C1 | OID20407 | TG | Strong | TRUE | 0.24 (0.21, 0.27) | 1.2E-41 | 4.0E-41 |
| ANGPTL4 | Q9BY76 | OID20703 | HDL-C | FALSE | FALSE | 0.1 (-0.01, 0.2) | 0.06 | 0.10 |
| APOC1 | P02654 | OID30749 | HDL-C | FALSE | FALSE | -0.07 (-0.16, 0.03) | 0.16 | 0.21 |
| APOC1 | P02654 | OID30749 | Non-HDL-C | FALSE | TRUE | 0.16 (-0.21, 0.53) | 0.41 | 0.41 |
| APOC1 | P02654 | OID30749 | TC | FALSE | TRUE | 0.13 (-0.16, 0.42) | 0.38 | 0.45 |
| APOC1 | P02654 | OID30749 | TG | FALSE | TRUE | 0.29 (0.21, 0.36) | 2.3E-13 | 3.9E-13 |
| APOE | P02649 | OID30727 | HDL-C | Strong | TRUE | 0.08 (0.03, 0.12) | 7.6E-04 | 2.0E-03 |
| APOE | P02649 | OID30727 | LDL-C | FALSE | FALSE | -0.52 (-0.7, -0.34) | 1.3E-08 | 3.1E-08 |
| APOE | P02649 | OID30727 | Non-HDL-C | Strong | TRUE | -0.46 (-0.61, -0.32) | 4.6E-10 | 1.0E-09 |
| APOE | P02649 | OID30727 | TC | Strong | TRUE | -0.37 (-0.48, -0.27) | 6.3E-12 | 1.7E-11 |
| APOE | P02649 | OID30727 | TG | FALSE | FALSE | 0.06 (-0.04, 0.15) | 0.22 | 0.28 |
| CELSR2 | Q9HCU4 | OID30593 | HDL-C | Strong | TRUE | 0.06 (0, 0.11) | 0.05 | 0.08 |
| CELSR2 | Q9HCU4 | OID30593 | LDL-C | Strong | TRUE | -0.29 (-0.41, -0.18) | 3.2E-07 | 5.7E-07 |
| CELSR2 | Q9HCU4 | OID30593 | Non-HDL-C | Strong | TRUE | -0.27 (-0.38, -0.15) | 3.9E-06 | 7.0E-06 |
| CELSR2 | Q9HCU4 | OID30593 | TC | Strong | TRUE | -0.25 (-0.34, -0.15) | 3.0E-07 | 6.5E-07 |
| CGREF1 | Q99674 | OID20152 | TG | FALSE | TRUE | -0.01 (-0.09, 0.07) | 0.74 | 0.74 |
| DPEP2 | Q9H4A9 | OID21305 | HDL-C | FALSE | TRUE | 0.35 (0.33, 0.37) | 7.0E-236 | 1.1E-234 |
| EPPK1 | P58107 | OID30250 | LDL-C | Strong | TRUE | -0.02 (-0.06, 0.02) | 0.34 | 0.40 |
| EPPK1 | P58107 | OID30250 | Non-HDL-C | Suggestive | TRUE | -0.03 (-0.07, 0.01) | 0.19 | 0.21 |
| EPPK1 | P58107 | OID30250 | TC | FALSE | FALSE | -0.02 (-0.06, 0.01) | 0.23 | 0.36 |
| GAS6 | Q14393 | OID20318 | Non-HDL-C | FALSE | TRUE | -0.08 (-0.1, -0.07) | 1.4E-37 | 6.2E-37 |
| GAS6 | Q14393 | OID20318 | TC | Strong | TRUE | -0.08 (-0.09, -0.07) | 4.0E-47 | 2.2E-46 |
| GAS6 | Q14393 | OID20318 | TG | Suggestive | TRUE | -0.07 (-0.09, -0.06) | 3.9E-45 | 2.0E-44 |
| GIPC3 | Q8TF64 | OID31507 | HDL-C | Suggestive | TRUE | -0.01 (-0.02, 0) | 0.17 | 0.21 |
| GSTA1 | P08263 | OID20166 | TG | Strong | TRUE | -0.06 (-0.07, -0.05) | 1.1E-26 | 2.2E-26 |
| GSTA3 | Q16772 | OID21242 | TG | Strong | TRUE | -0.06 (-0.07, -0.05) | 8.7E-27 | 2.2E-26 |
| GSTM4 | Q03013 | OID30164 | HDL-C | FALSE | FALSE | -0.09 (-0.2, 0.02) | 0.11 | 0.15 |
| HGFAC | Q04756 | OID30718 | LDL-C | Strong | TRUE | -0.01 (-0.04, 0.02) | 0.53 | 0.53 |
| HGFAC | Q04756 | OID30718 | Non-HDL-C | Strong | TRUE | -0.03 (-0.05, -0.01) | 5.2E-03 | 7.8E-03 |
| HGFAC | Q04756 | OID30718 | TC | Strong | TRUE | -0.02 (-0.06, 0.02) | 0.40 | 0.45 |
| KHK | P50053 | OID30241 | TG | FALSE | FALSE | 0.04 (-0.11, 0.2) | 0.58 | 0.64 |
| LILRA3 | Q8N6C8 | OID30376 | HDL-C | FALSE | TRUE | -0.04 (-0.05, -0.03) | 2.3E-11 | 1.2E-10 |
| LILRA5 | A6NI73 | OID20209 | HDL-C | FALSE | FALSE | -0.09 (-0.15, -0.03) | 2.5E-03 | 5.8E-03 |
| LILRB2 | Q8N423 | OID20301 | HDL-C | FALSE | TRUE | -0.03 (-0.07, 0.02) | 0.28 | 0.30 |
| LILRB5 | O75023 | OID20324 | HDL-C | FALSE | TRUE | 0.03 (0.02, 0.05) | 4.1E-06 | 1.3E-05 |
| LPA | P08519 | OID30747 | LDL-C | Strong | TRUE | 0.07 (0.03, 0.11) | 6.7E-04 | 9.3E-04 |
| LPA | P08519 | OID30747 | TC | Strong | TRUE | 0.05 (0.02, 0.09) | 3.2E-03 | 5.9E-03 |
| PCSK7 | Q16549 | OID31410 | TG | FALSE | FALSE | -0.18 (-0.36, 0.01) | 0.06 | 0.08 |
| PCSK9 | Q8NBP7 | OID20235 | LDL-C | Strong | TRUE | 0.37 (0.36, 0.39) | 0 | 0 |
| PCSK9 | Q8NBP7 | OID20235 | nonHDL-C | Strong | TRUE | 0.35 (0.34, 0.37) | 0 | 0 |
| PCSK9 | Q8NBP7 | OID20235 | TC | Strong | TRUE | 0.32 (0.31, 0.34) | 0 | 0 |
| PECR | Q9BY49 | OID30228 | HDL-C | Suggestive | TRUE | 0.01 (0, 0.02) | 0.21 | 0.24 |
| PLA2G15 | Q8NCC3 | OID21473 | HDL-C | Strong | TRUE | -0.06 (-0.11, 0) | 0.04 | 0.08 |
| PLTP | P55058 | OID20238 | TG | Strong | TRUE | -0.1 (-0.11, -0.09) | 3.6E-72 | 3.6E-71 |
| THOP1 | P52888 | OID20194 | HDL-C | FALSE | FALSE | 0.03 (0.02, 0.04) | 4.2E-09 | 1.7E-08 |
| TLR3 | O15455 | OID20612 | HDL-C | Suggestive | FALSE | 0 (-0.01, 0.01) | 0.93 | 0.93 |
| TNFSF13 | O75888 | OID20733 | Non-HDL-C | Suggestive | FALSE | 0.03 (-0.01, 0.06) | 0.12 | 0.16 |
| TNFSF13 | O75888 | OID20733 | TC | FALSE | FALSE | 0.02 (-0.02, 0.06) | 0.41 | 0.45 |

# **Supplementary Table S14. Observational associations of plasma protein levels with lipid fractions**

| Protein | Outcome | Beta (95% Confidence Interval) | P | FDR |
| --- | --- | --- | --- | --- |
| APOE | HDL | 0.08 (0.07,0.08) | 2.8E-98 | 1.2E-97 |
| APOE*south.asian | HDL | -0.01 (-0.05,0.02) | 7.0E-01 | 7.4E-01 |
| CELSR2 | HDL | -0.04 (-0.05,-0.04) | 3.6E-31 | 9.2E-31 |
| CELSR2*south.asian | HDL | 0.02 (-0.01,0.05) | 5.7E-01 | 6.5E-01 |
| PLA2G15 | HDL | -0.07 (-0.07,-0.06) | 1.4E-67 | 4.1E-67 |
| PLA2G15*south.asian | HDL | -0.04 (-0.07,-0.01) | 1.9E-01 | 2.6E-01 |
| ANGPTL3 | LDL | 0.18 (0.18,0.18) | 0.0E+00 | 0.0E+00 |
| ANGPTL3*south.asian | LDL | -0.07 (-0.1,-0.04) | 2.3E-02 | 3.5E-02 |
| CELSR2 | LDL | 0.03 (0.03,0.04) | 9.9E-17 | 2.0E-16 |
| CELSR2*south.asian | LDL | -0.02 (-0.05,0.01) | 5.7E-01 | 6.5E-01 |
| EPPK1 | LDL | 0.04 (0.03,0.04) | 4.9E-20 | 1.1E-19 |
| EPPK1*south.asian | LDL | 0 (-0.03,0.03) | 9.6E-01 | 9.6E-01 |
| HGFAC | LDL | 0.11 (0.1,0.11) | 1.2E-144 | 7.2E-144 |
| HGFAC*south.asian | LDL | -0.09 (-0.13,-0.06) | 7.5E-03 | 1.2E-02 |
| LPA | LDL | 0.08 (0.07,0.08) | 2.1E-75 | 7.5E-75 |
| LPA*south.asian | LDL | -0.02 (-0.06,0.01) | 4.9E-01 | 6.3E-01 |
| PCSK9 | LDL | 0.2 (0.19,0.2) | 0.0E+00 | 0.0E+00 |
| PCSK9*south.asian | LDL | -0.14 (-0.17,-0.11) | 2.2E-05 | 4.0E-05 |

# **Supplementary Table S15. Summary table of plasma proteins associated with lipid fractions**

| **Protein** | **Lipid fraction affected**  **(*indicates suggestive colocalisation)** | **Known functions/pathways**  **(* indicates pathways involving lipid metabolism)** | **Evidence** | **New Finding** |
| --- | --- | --- | --- | --- |
| PCSK9 | LDL-C, non-HDL-C, TC | (1) VLDLR internalisation and degradation*; (2) LDL clearance*; (3) Regulation of Insulin-like Growth Factor (IGF) transport and uptake by Insulin-like Growth Factor Binding Proteins (IGFBPs); (4) Post-translational protein phosphorylation | Experimental evidence ^8^ Population evidence in European ^8^ Population evidence in South Asian ^9^ | PCSK9‘s effect in South Asian is weaker |
| ANGPTL3 | LDL-C, HDL-C*, non-HDL-C, TC, TG | (1) NR1H2 & NR1H3 regulate gene expression linked to lipogenesis*; (2) Assembly of active LPL and LIPC lipase complexes* | Experimental evidence ^10^ Population evidence in European ^11^ | Validate in South Asian |
| LPA | LDL-C, TC | (1) LDL remodelling* | Experimental evidence ^12^ Population evidence in European ^13^ | Validate in South Asian |
| PLTP | TG | (1) HDL remodelling*; (2) NR1H3 & NR1H2 regulate gene expression linked to cholesterol transport and efflux* | Experimental evidence ^14^ Population evidence in European ^14^ | Validate in South Asian |
| APOE | HDL-C, TC, non-HDL-C | (1) Nuclear signalling by ERBB4; (2) Retinoid metabolism and transport; (3) Chylomicron clearance*; (4) Scavenging by Class A Receptors; (5) Chylomicron assembly*; (6) Regulation of Insulin-like Growth Factor (IGF) transport and uptake by Insulin-like Growth Factor Binding Proteins (IGFBPs); (7) Post-translational protein phosphorylation; (8) HDL remodelling*; (9) Amyloid fiber formation; (10) Transcriptional regulation by the AP-2 (TFAP2) family of transcription factors; (11) NR1H3 & NR1H2 regulate gene expression linked to cholesterol transport and efflux*; (12) Chylomicron remodelling* | Experimental evidence ^15^ | Validate in South Asian |
| CELSR2 | LDL-C, HDL-C, TC, non-HDL-C | / | Experimental evidence ^16^ | Discovered in South Asian |
| GAS6 | TC, TG* | (1) Platelet degranulation; (2) Gamma-carboxylation of protein precursors; (3) Transport of gamma-carboxylated protein precursors from the endoplasmic reticulum to the Golgi apparatus; (4) Removal of aminoterminal propeptides from gamma-carboxylated proteins; (5) Cell surface interactions at the vascular wall; (6) Regulation of Insulin-like Growth Factor (IGF) transport and uptake by Insulin-like Growth Factor Binding Proteins (IGFBPs); (7) Post-translational protein phosphorylation | Experimental evidence ^17^ Population evidence in European ^17^ | Validated in South Asian |
| HGFAC | LDL-C, TC, non-HDL-C | (1) MET Receptor Activation; | / | Discovered in South Asian |
| PECR | HDL-C | (1) Peroxisomal protein import; (2) Alpha-oxidation of phytanate | / | Discovered in South Asian |
| EPPK1 | LDL-C, non-HDL-C* | / | / | Discovered in South Asian |
| GSTA1 | TG | (1) Azathioprine ADME; (2) Haeme degradation; (3) Glutathione conjugation; (4) NFE2L2 regulating anti-oxidant/detoxification enzymes | / | Discovered in South Asian |
| GSTA3 | TG | (1) Glutathione conjugation; (2) NFE2L2 regulating anti-oxidant/detoxification enzymes | / | Discovered in South Asian |
| GIPC3 | HDL-C* | / | / | Discovered in South Asian |
| PECR | HDL-C* | (1) Peroxisomal protein import; (2) Alpha-oxidation of phytanate | / | Discovered in South Asian |
| PLA2G15 | HDL-C | (1) Hydrolysis of LPC | / | Discovered in South Asian |


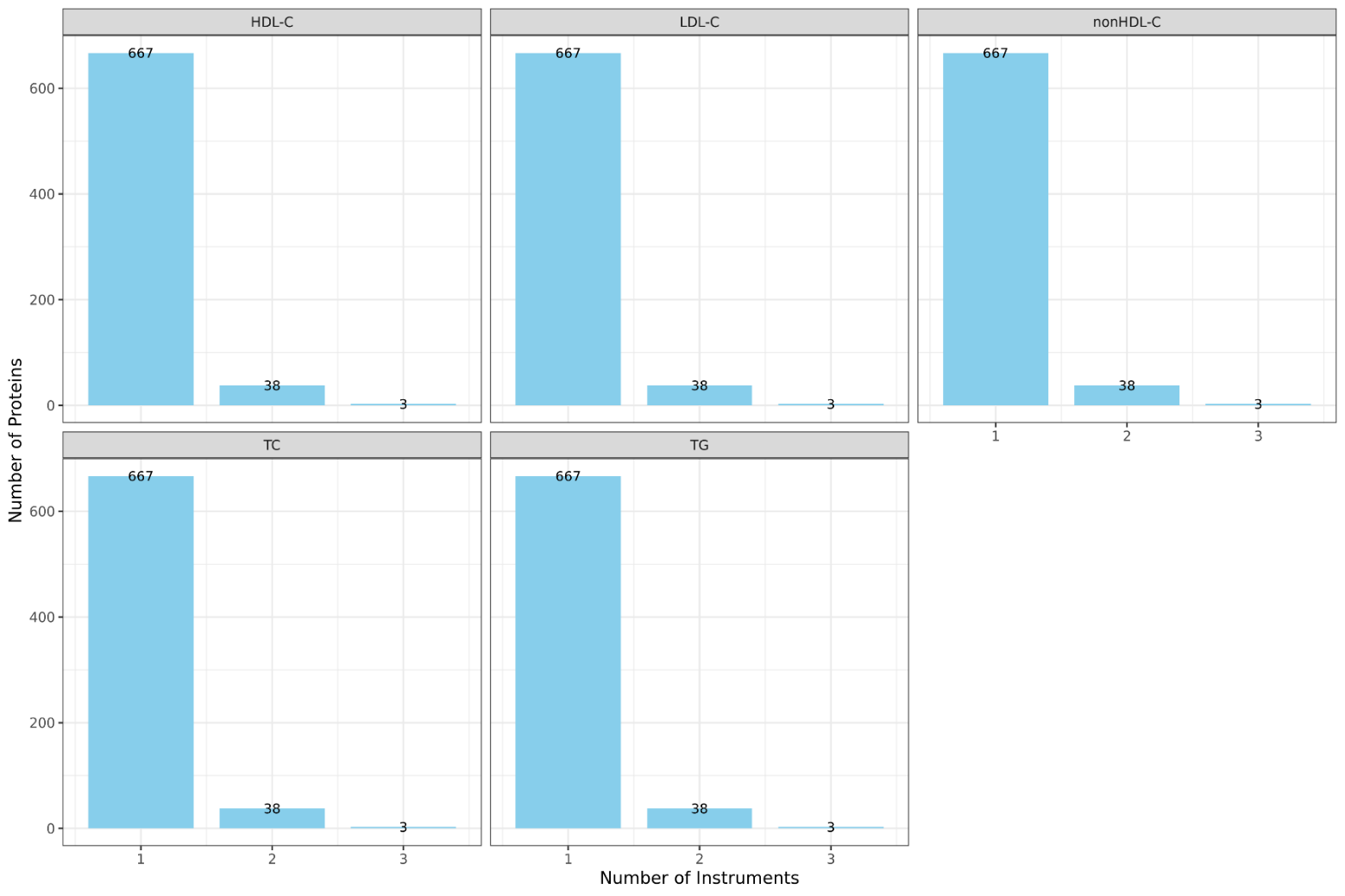


# **Supplementary Figure S1. Number of instruments for plasma proteins**

**
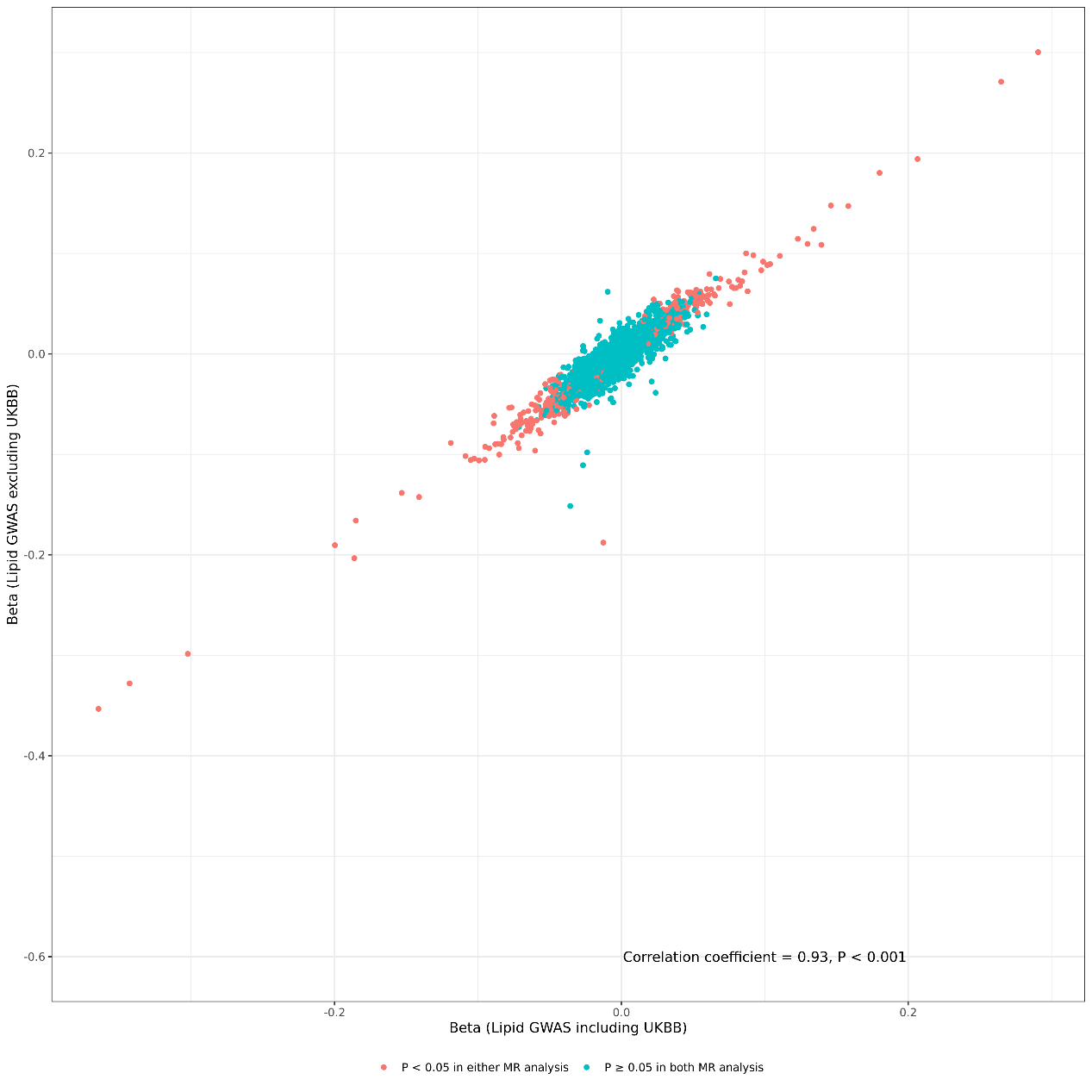
**

# **Supplementary Figure S2A. Scatter plot comparing proteome-wide MR result including or excluding UK Biobank data in GWAS on lipid fractions**

**
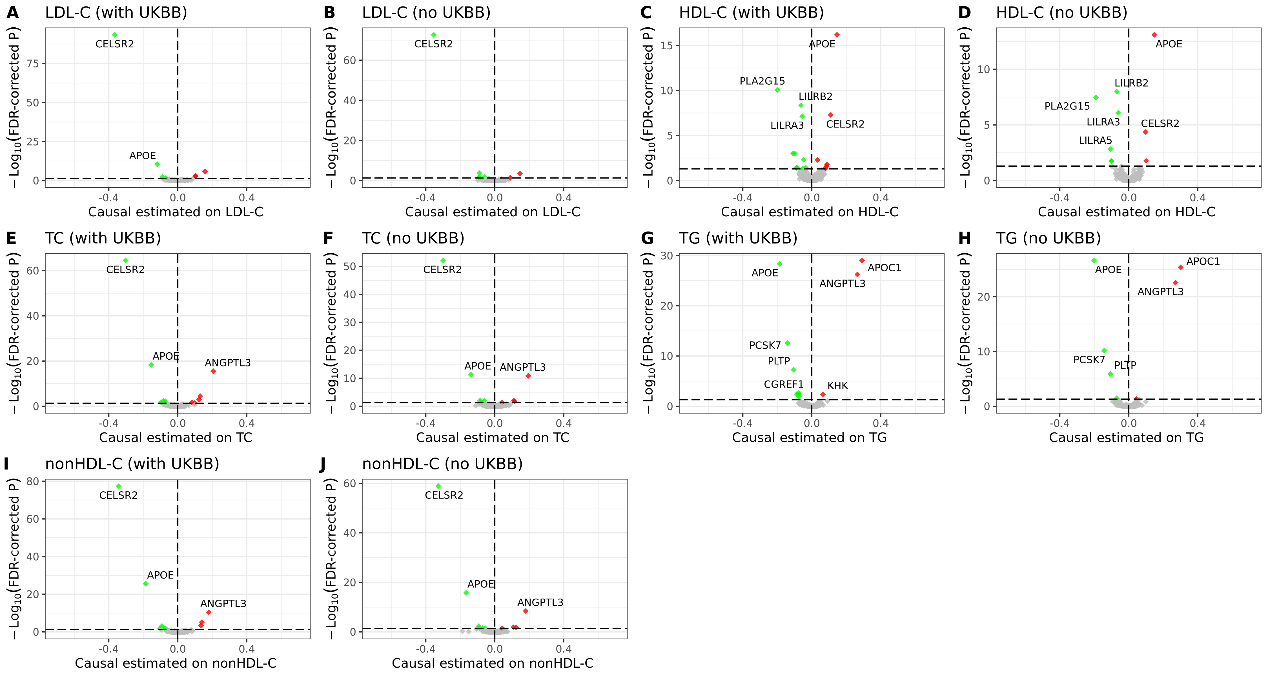
**

# **Supplementary Figure S2B. Volcano plot comparing proteome-wide MR result including or excluding UK Biobank data in GWAS on lipid fractions**

**
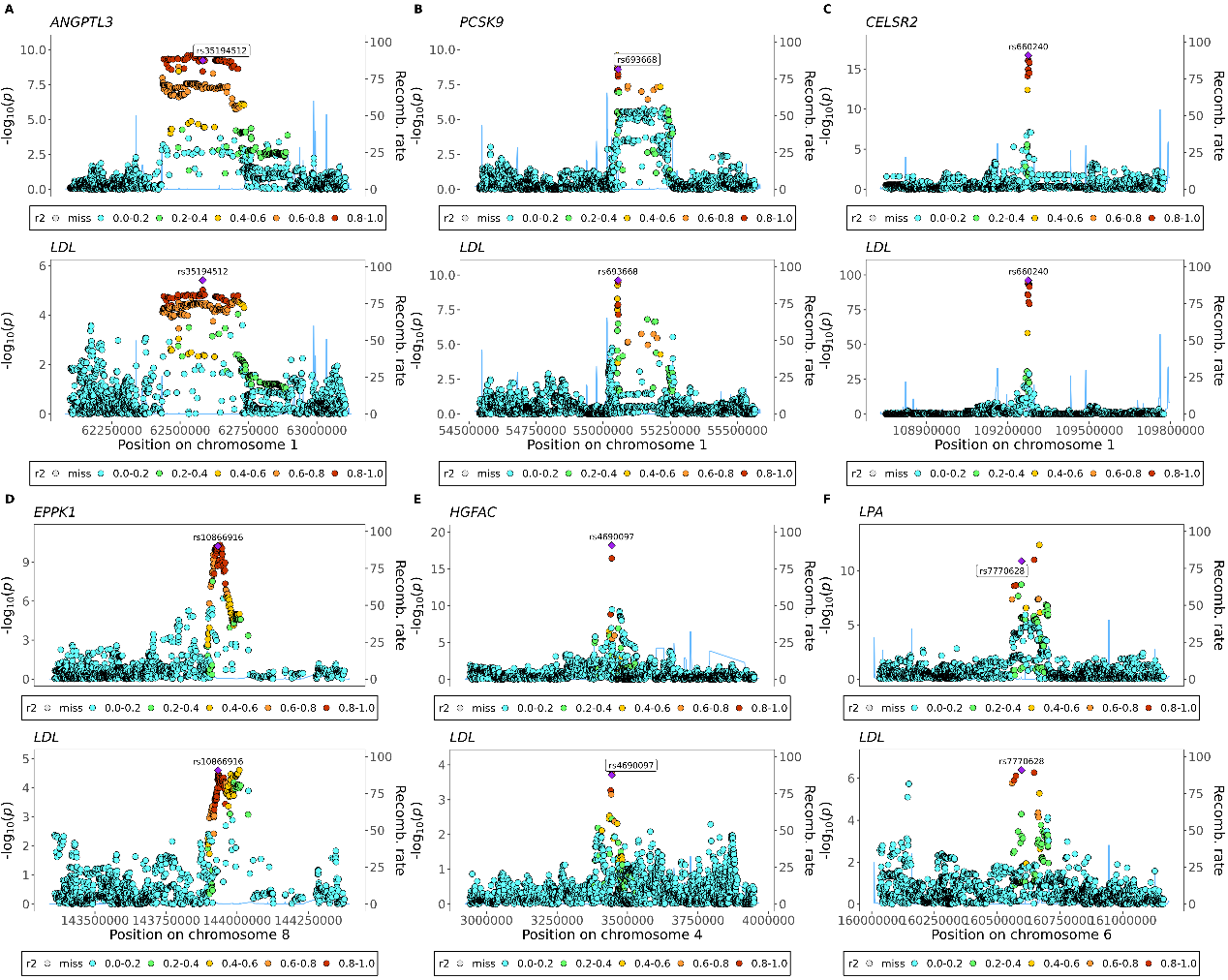
**

# **Supplementary Figure S3A. Regional genomic plot for colocalisation between plasma proteins and LDL-C**

**
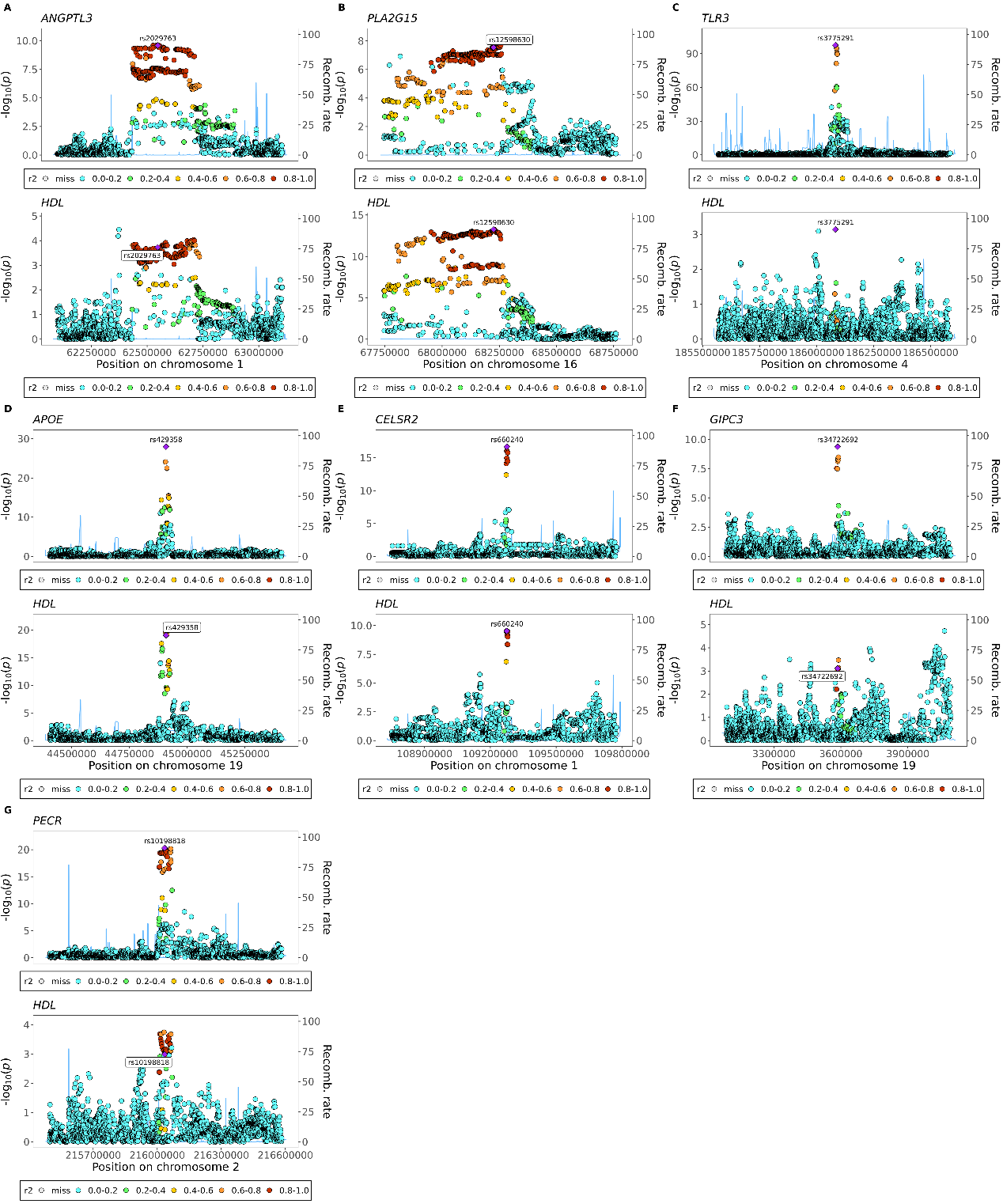
**

# **Supplementary Figure S3B. Regional genomic plot for colocalisation between plasma proteins and HDL-C**

**
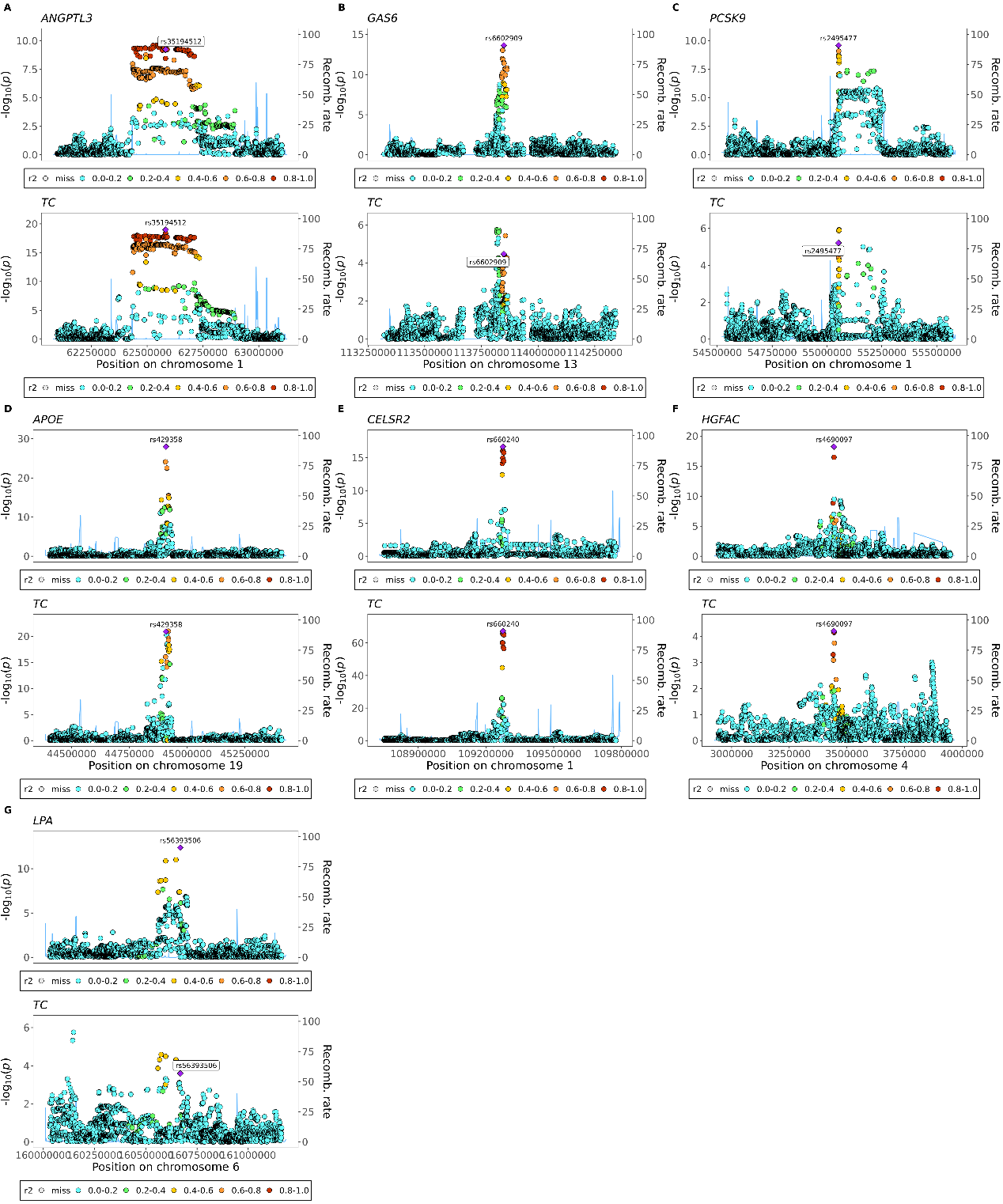
**

# **Supplementary Figure S3C. Regional genomic plot for colocalisation between plasma proteins and TC**

**
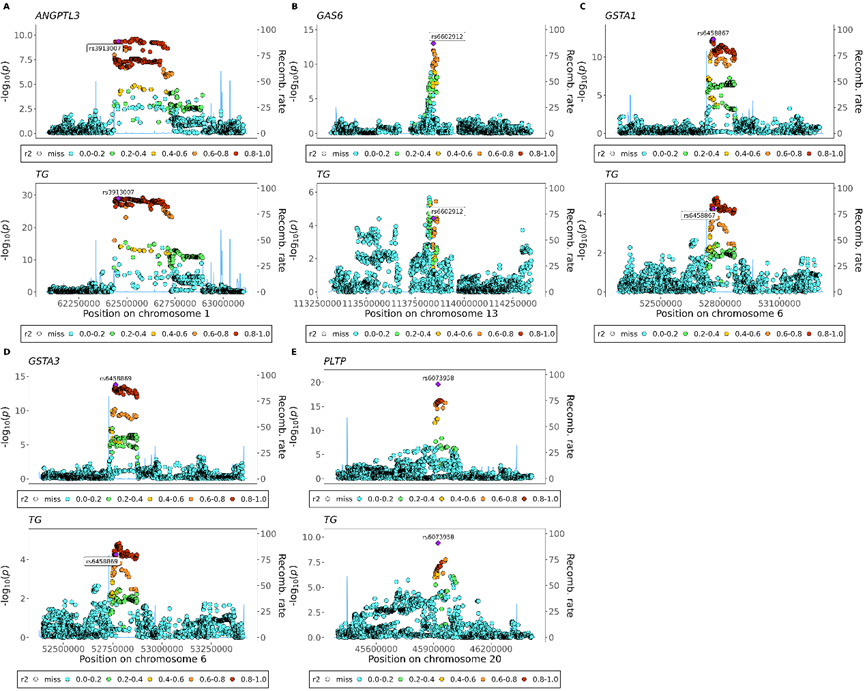
**

# **Supplementary Figure S3D. Regional genomic plot for colocalisation between plasma proteins and TG**

**
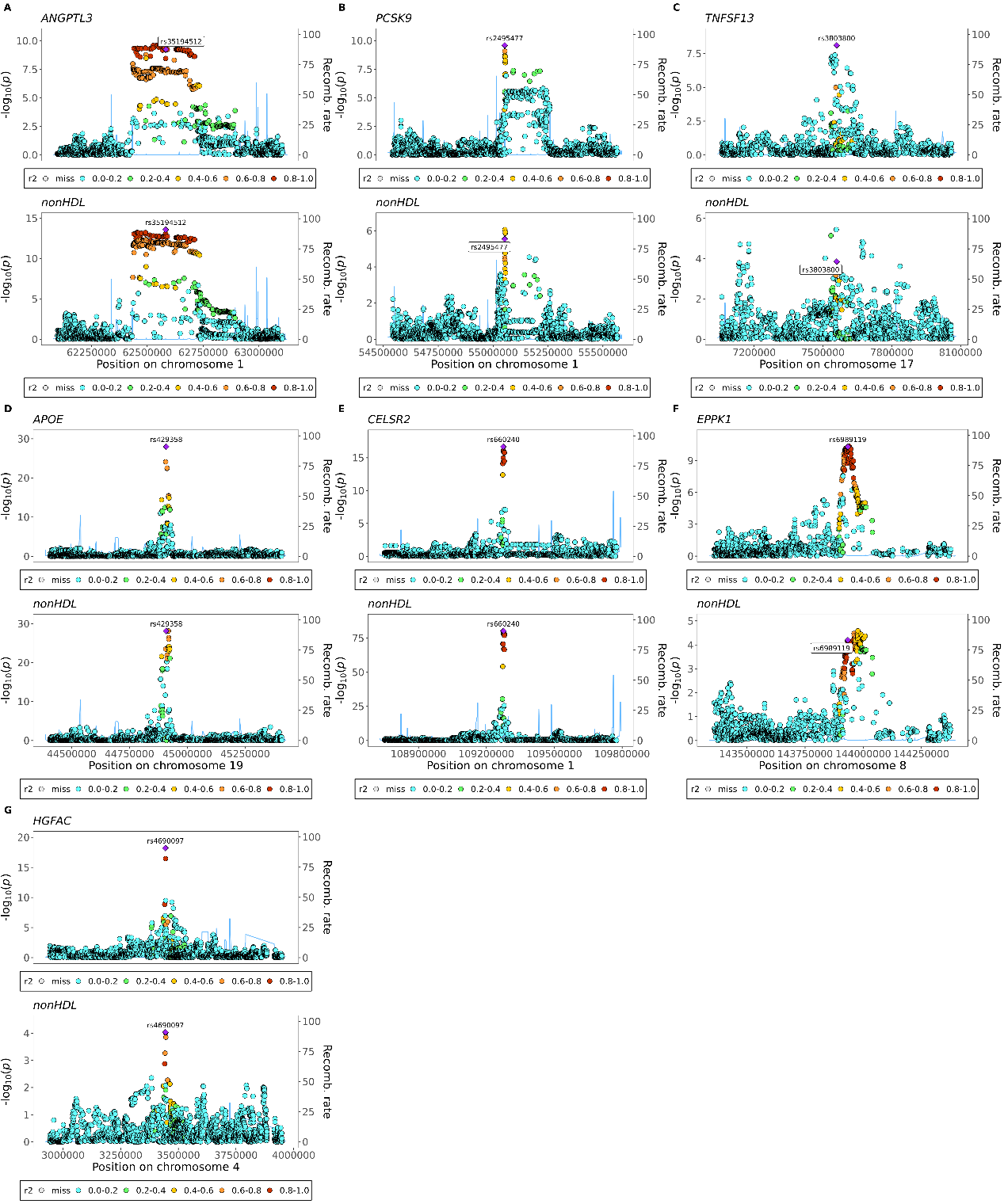
**

# **Supplementary Figure S3E. Regional genomic plot for colocalisation between plasma proteins and nonHDL-C**

**
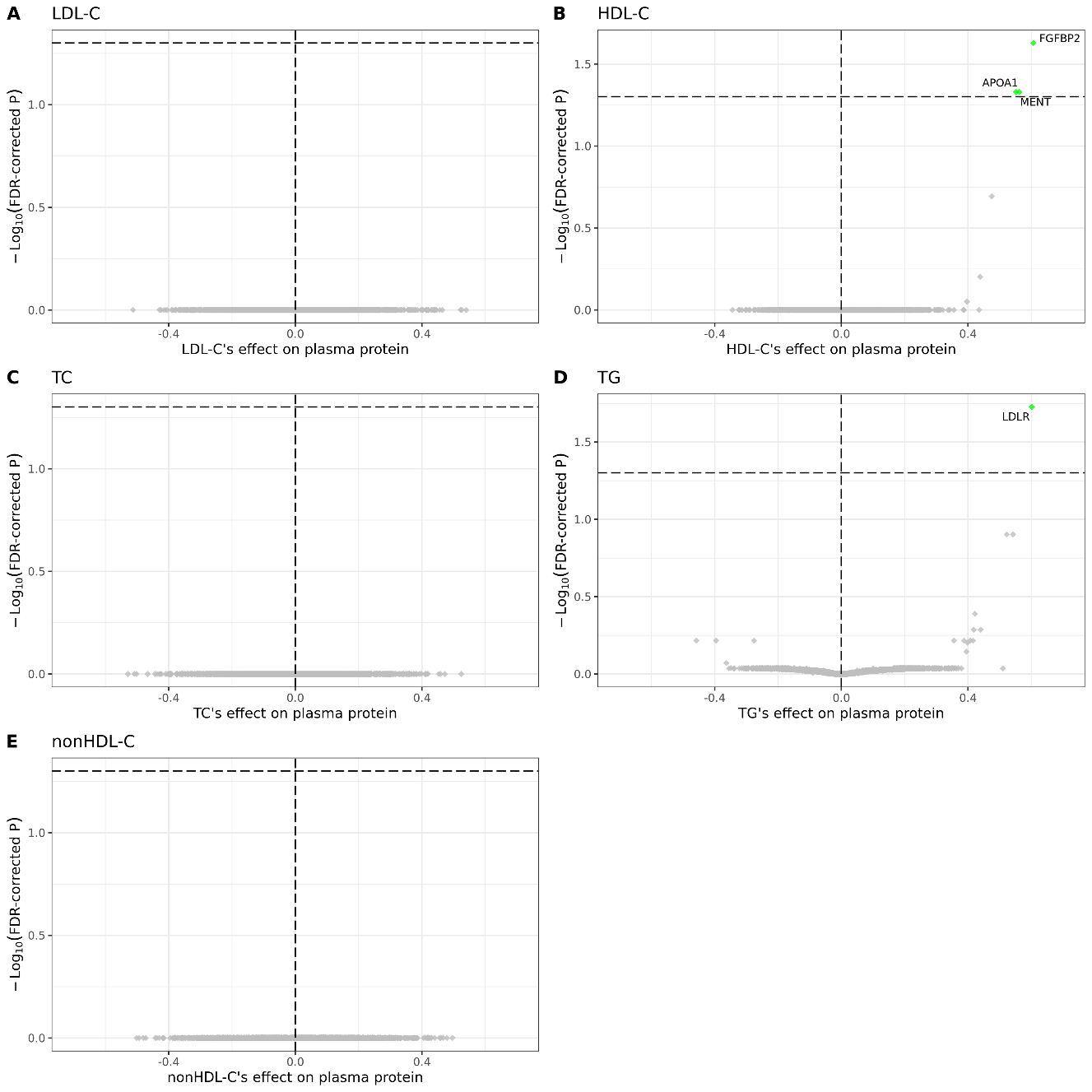
**

# **Supplementary Figure S4. Volocanoe plot of reverse MR evaluating lipid fractions’ effect on plasma protein**

**
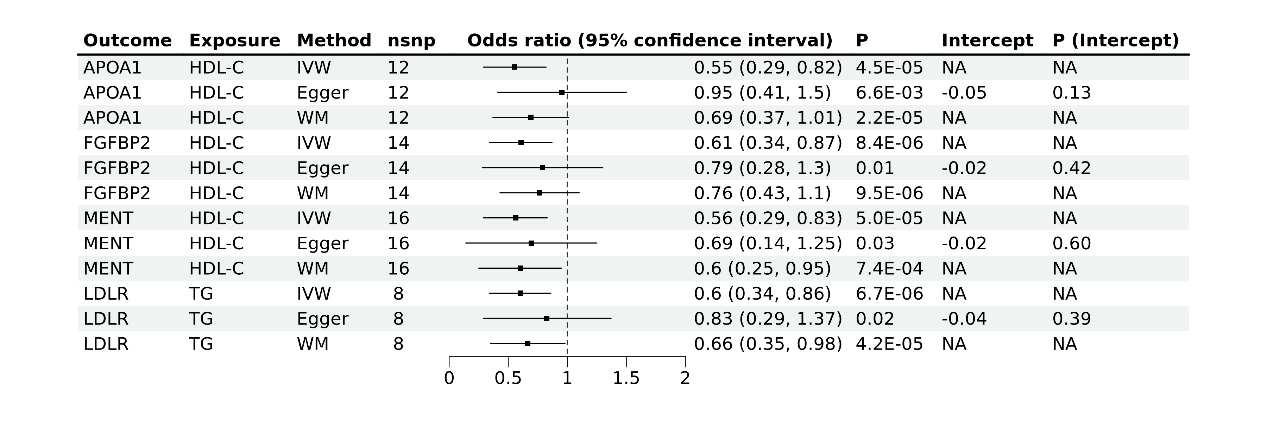
**

# **Supplementary Figure S5. Forest plot of reverse MR identified plasma proteins effected by lipid fractions**

**
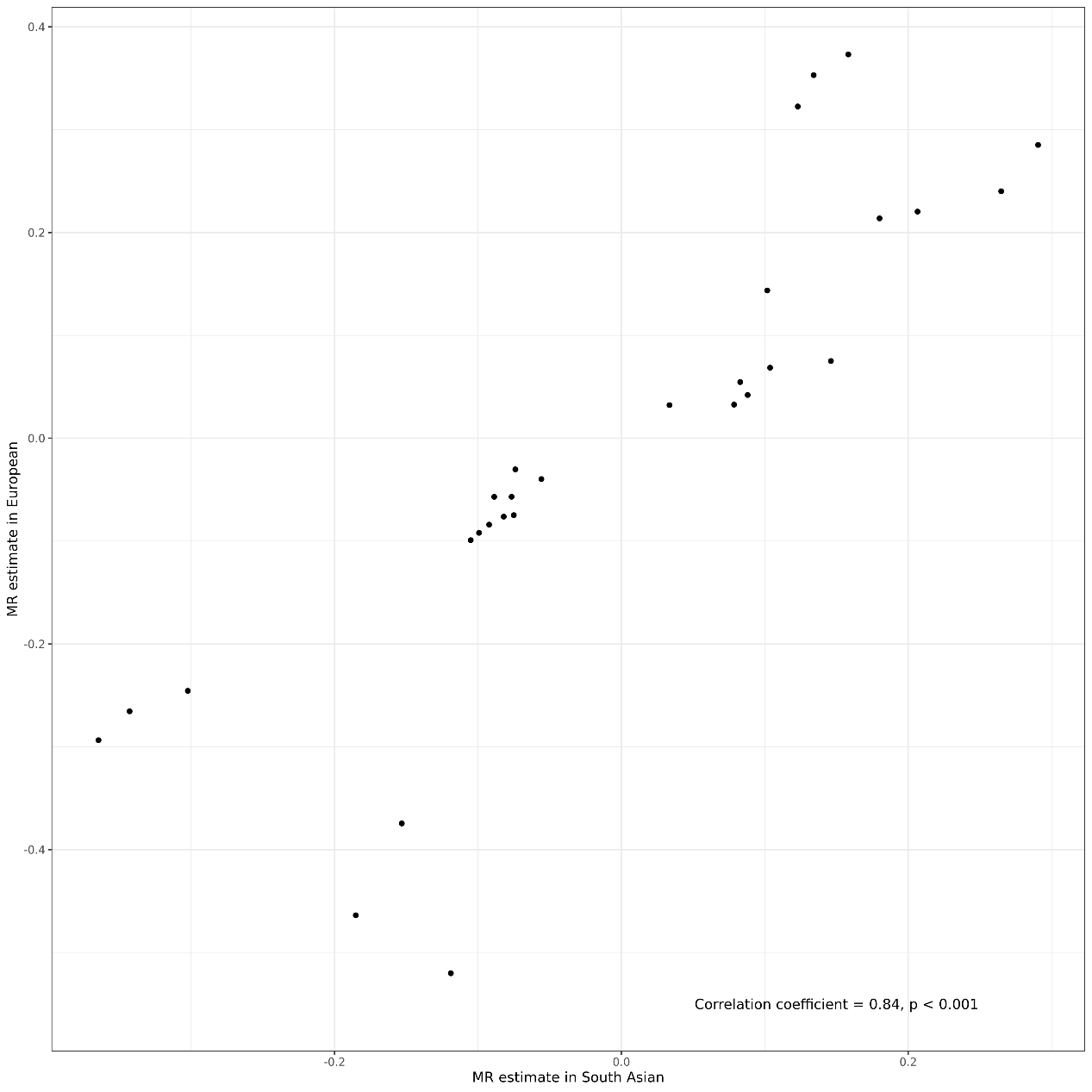
**

# **Supplementary Figure S6. Scatter plot comparing proteome-wide MR estimates in South Asian versus European**

# **Reference**

1. Siva N. 1000 Genomes project. *Nature Biotechnology* 2008; **26**(3): 256-.

2. Cavalli-Sforza LL. The Human Genome Diversity Project: past, present and future. *Nature Reviews Genetics* 2005; **6**(4): 333-40.

3. Finer S, Martin HC, Khan A, et al. Cohort Profile: East London Genes &amp; Health (ELGH), a community-based population genomics and health study in British Bangladeshi and British Pakistani people. *International Journal of Epidemiology* 2019; **49**(1): 20-1i.

4. Mishra A, Malik R, Hachiya T, et al. Stroke genetics informs drug discovery and risk prediction across ancestries. *Nature* 2022; **611**(7934): 115-23.

5. Nikpay M, Goel A, Won H-H, et al. A comprehensive 1000 Genomes–based genome-wide association meta-analysis of coronary artery disease. *Nature Genetics* 2015; **47**(10): 1121-30.

6. Pierce BL, Ahsan H, Vanderweele TJ. Power and instrument strength requirements for Mendelian randomization studies using multiple genetic variants. *Int J Epidemiol* 2011; **40**(3): 740-52.

7. Hemani G, Tilling K, Davey Smith G. Orienting the causal relationship between imprecisely measured traits using GWAS summary data. *PLoS Genet* 2017; **13**(11): e1007081.

8. Dadu RT, Ballantyne CM. Lipid lowering with PCSK9 inhibitors. *Nat Rev Cardiol* 2014; **11**(10): 563-75.

9. Bansal S, Ruzza A, Sawhney J, et al. Evolocumab in patients with homozygous familial hypercholesterolemia in India. *J Clin Lipidol* 2021; **15**(6): 814-21.

10. Koishi R, Ando Y, Ono M, et al. Angptl3 regulates lipid metabolism in mice. *Nat Genet* 2002; **30**(2): 151-7.

11. Raal FJ, Rosenson RS, Reeskamp LF, et al. Evinacumab for Homozygous Familial Hypercholesterolemia. *N Engl J Med* 2020; **383**(8): 711-20.

12. Lobentanz EM, Krasznai K, Gruber A, et al. Intracellular metabolism of human apolipoprotein(a) in stably transfected Hep G2 cells. *Biochemistry* 1998; **37**(16): 5417-25.

13. Viney NJ, Yeang C, Yang X, Xia S, Witztum JL, Tsimikas S. Relationship between "LDL-C", estimated true LDL-C, apolipoprotein B-100, and PCSK9 levels following lipoprotein(a) lowering with an antisense oligonucleotide. *J Clin Lipidol* 2018; **12**(3): 702-10.

14. Albers JJ, Vuletic S, Cheung MC. Role of plasma phospholipid transfer protein in lipid and lipoprotein metabolism. *Biochim Biophys Acta* 2012; **1821**(3): 345-57.

15. Huang Y, Mahley RW. Apolipoprotein E: structure and function in lipid metabolism, neurobiology, and Alzheimer's diseases. *Neurobiol Dis* 2014; **72 Pt A**: 3-12.

16. Tan J, Che Y, Liu Y, et al. CELSR2 deficiency suppresses lipid accumulation in hepatocyte by impairing the UPR and elevating ROS level. *Faseb j* 2021; **35**(10): e21908.

17. Bordoloi J, Ozah D, Bora T, Kalita J, Manna P. Gamma-glutamyl carboxylated Gas6 mediates the beneficial effect of vitamin K on lowering hyperlipidemia via regulating the AMPK/SREBP1/PPARα signaling cascade of lipid metabolism. *J Nutr Biochem* 2019; **70**: 174-84.
